# Native AMF Communities in an Italian Vineyard at Two Different Phenological Stages of *Vitis vinifera*

**DOI:** 10.3389/fmicb.2021.676610

**Published:** 2021-07-19

**Authors:** Patrizia Cesaro, Nadia Massa, Elisa Bona, Giorgia Novello, Valeria Todeschini, Lara Boatti, Flavio Mignone, Elisa Gamalero, Graziella Berta, Guido Lingua

**Affiliations:** ^1^Dipartimento di Scienze e Innovazione Tecnologica, Università del Piemonte Orientale, Alessandria, Italy; ^2^Dipartimento di Scienze e Innovazione Tecnologica, Università del Piemonte Orientale, Vercelli, Italy; ^3^SmartSeq s.r.l., spin-off of the Università del Piemonte Orientale, Alessandria, Italy

**Keywords:** *Vitis vinifera*, arbuscular mycorrhizal fungi, biodiversity, conventional management, soil, grapevine roots

## Abstract

Arbuscular mycorrhizal fungi (AMF) are beneficial soil microorganisms that can establish symbiotic associations with *Vitis vinifera* roots, resulting in positive effects on grapevine performance, both in terms of water use efficiency, nutrient uptake, and replant success. Grapevine is an important perennial crop cultivated worldwide, especially in Mediterranean countries. In Italy, Piedmont is one of the regions with the longest winemaking tradition. In the present study, we characterized the AMF communities of the soil associated or not with the roots of *V. vinifera* cv. Pinot Noir cultivated in a vineyard subjected to conventional management using 454 Roche sequencing technology. Samplings were performed at two plant phenological stages (flowering and early fruit development). The AMF community was dominated by members of the family Glomeraceae, with a prevalence of the genus *Glomus* and the species *Rhizophagus intraradices* and *Rhizophagus irregularis*. On the contrary, the genus *Archaeospora* was the only one belonging to the family Archaeosporaceae. Since different AMF communities occur in the two considered soils, independently from the plant phenological stage, a probable role of *V. vinifera* in determining the AMF populations associated to its roots has been highlighted.

## Introduction

Arbuscular mycorrhizal fungi (AMF) are beneficial symbiotic soil microorganisms that improve the plant nutritional state by increasing the interface area between roots and soil (Hodge et al., 2010). Moreover, the arbuscular mycorrhizal symbiosis provides other advantages for plants, such as better tolerance versus biotic or abiotic stresses (Hodge et al., 2010; Bona et al., 2011; Lingua et al., 2012; Degola et al., 2015) and improved fruit yield and quality (Baslam et al., 2013; Berta et al., 2014; Bona et al., 2015, 2017, 2018; Todeschini et al., 2018).

Grapevine (*Vitis vinifera* L.) is an important perennial crop cultivated in all continents where the climatic conditions are permissive. Italy is one of the five major grape producers in the world, with about 8,600,000 tons which represent 11% of the production in the world (IOV, 2019). It has been widely demonstrated that the inclusion of fresh grape and its derivates in the diet (Vislocky and Fernandez, 2010) and also the reasonable consumption of wine (Georgiev et al., 2014; Artero et al., 2015) can give beneficial effects for human health, decreasing the risk factors associated with cancer and age-related cognitive decline as well as cardiovascular and neurodegenerative diseases (Torres et al., 2018). Pinot Noir is a grapevine cultivar from which both white and red fine wines, with typical organoleptic characteristics, are produced all over the world. In particular, the surface area dedicated to this cultivation corresponds to 112,000 ha. Germany, Italy, and Switzerland in Europe and United States, New Zealand, and Australia in non-European countries are the main producers (IOV, 2017). Grapevines, during their life cycle, are subjected to various cultivation practices which can interfere with the native microbiota and also the fungal soil population. It is well documented that practices like tillage, as well as the use of fertilizers and/or pesticides, can reduce soil microbial biodiversity (Berruti et al., 2014; Trouvelot et al., 2015; Zaller et al., 2018; Nogales et al., 2019). The intensity and frequency of these practices vary according to the type of vineyard management, which can be classified into conventional, organic, and/or integrated (Likar et al., 2017; Zaller et al., 2018). The growth and development of grapevines are dependent on AMF (Linderman and Davis, 2001; Schreiner, 2005), and the occurrence of species specificity between *V. vinifera* and AMF has been observed (Holland et al., 2014). In addition, if compared to non-native ones, AMF native of a certain area are often reported to be more effective in plant growth promotion (Schreiner et al., 2006). In order to realize and manage a sustainable agricultural ecosystem, the study of AMF communities associated with grapevines, in the context of conventional management, becomes of great importance (Likar et al., 2013). Several works described the biodiversity of AMF in vineyards subjected to conventional management. The AMF community of two differently managed vineyards (tilled and covered) in Sardinia was characterized by Lumini et al. (2010). Conventional management of the vineyard leads to the development of different fungal and bacterial microbial communities according to specific local biogeographic factors (Likar et al., 2017). The differences between AMF communities in a vineyard and in nearby unmanaged areas were analyzed in order to highlight the impact of viticulture on AMF community diversity and composition (Holland et al., 2016). Finally, AMF biodiversity was studied in the roots of *V. vinifera* cv. Pinot Noir and Chardonnay in Burgundy (France) and Oregon (United States) (Bouffaud et al., 2016; Schreiner, 2020). In the past, the study of AMF communities was exclusively based on the morphological identification of isolated spores. More recently, this methodology has been complemented and/or replaced by molecular techniques, applied both to roots and soils as, for example, cloning followed by Sanger sequencing (Vasar et al., 2017). Then, since the early 2000s, the use of next-generation sequencing, including Roche 454 platform, allowed the analysis of a huge number of sequences (hundreds of thousands) per sample, enormously increasing the depth of investigation. Molecular approaches are based on nuclear ribosomal markers such as the small subunit (SSU) rRNA gene, the internal transcribed spacer region (ITS), and the large subunit (LSU) rRNA gene (Öpik and Davison, 2016). Based on the idea that Piedmont has winemaking tradition that we can define as historic, it becomes of extreme ecological and applicative importance to get information on the AMF communities associated with the vines. In this geographical zone, we therefore identified a vineyard cultivated with grapevine cv. Pinot Noir and subjected to conventional management. A detailed description of the native AMF communities of the soils associated or not with the grapevine roots at two plant phenological stages (flowering and early fruit development) was obtained.

## Materials and Methods

### Soil Sampling

The experimental vineyard is located in Mantovana (Predosa municipality, Alessandria, Southern Piedmont, Italy – altitude: 215 m a.s.l., latitude: 44.730294° N, and longitude: 8.6226556°E), and it is subjected to conventional management. Glyphosate treatment was performed in the vineyard in June. Trifloxistrobin and Fosetyl-Al + copper were employed as fungicides against *Oidium* spp. and *Peronospora* spp., respectively, and were distributed in June and July, coupled with one insecticide (thiamethoxan) and two sulfur treatments in July.

**FIGURE 1:**
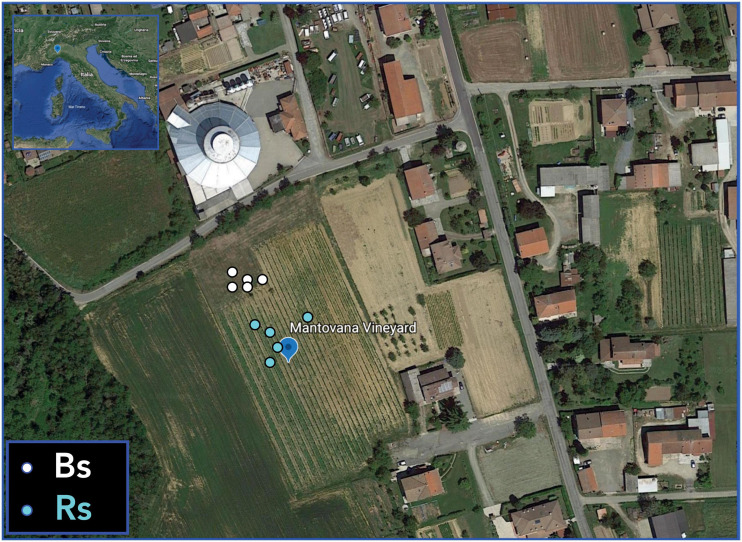
Vineyard aerial view showing the sampling points. The vineyard is located in Mantovana (Predosa municipality, Alessandria, Italy). The two soil sampling sites included one in an area just outside the borders of the vineyard in the absence of grapevines (Bs, white dots) and one inside the vineyard corresponding to the grapevine plants (Rs, cyan dots). Google Earth online version was used to produce this image (https://earth.google.com/web/).

The soil, hereafter indicated as Bs, was sampled close to the vineyard, in a not cultivated area covered in part with grasses (Figure 1). The soil associated to the roots (Rs) of *V. vinifera* cv. Pinot Noir, grafted onto SO4 rootstock, was collected from grapevine roots entrapped in the soil cores taken near the plant. Samplings were carried out in May 2014 (Bs1S and Rs1S, flowering) and July 2014 (Bs2S and Rs2S, early fruit development). For each soil (Bs or Rs) and time point (1S or 2S), five samples were collected from the topsoil (5–30 cm). According to the Italian guide for soil analysis (GU 179/2002), for each plant, three soil cores were taken, pooled, and mixed to prepare one sample. The soil samples were stored at −20°C until DNA extraction.

The soil of the vineyard was clay-loam and acidic, as reported in Gamalero et al. (2020); moreover, the climatic conditions of the area, such as temperature, humidity, and rainfall, are detailed in Bona et al. (2019).

### AMF Root Colonization

Mycorrhizal colonization was evaluated microscopically following the method of Trouvelot et al. (1986) as mycorrhizal frequency, degree of AMF root colonization, and arbuscule and vesicle abundance. Briefly, from grapevine roots, 30 randomly chosen 1-cm-long pieces were cut, cleared at 80°C for 90 min in 10% KOH, stained with 1% methyl blue in lactic acid, and mounted onto slides. The results were analyzed by ANOVA; differences were considered statistically significant for *p*-values less than 0.05.

### DNA Extraction and Amplification

Power Soil R DNA Isolation Kit (MO BIO Laboratories, Inc., Carlsbad, CA, United States) was used, following the manufacturer’s instructions, to extract DNA from five samples, both of Bs and of the soil associated with the roots of *V. vinifera* (Rs), collected at flowering (1S) or at fruiting (2S) times. A hemi-nested PCR, employing as template the previously extracted DNA, was performed using LR1 and FLR2 (Cesaro et al., 2008) primers for the first amplification and LR1 and FLR4 (Cesaro et al., 2008) primers tagged with Multiplex Identifier sequences for 454 Pyrosequencing (Roche) for the second one. In particular, the FLR2 and FLR4 primers are specific for fungi and for Glomeromycota, respectively (Farmer et al., 2007). The reactions were performed at the conditions described in Massa et al. (2020).

Pyrosequencing employing 454 technology was performed on the products of the second PCR (size, 700 bp). DNA-carrying beads were loaded on a PicoTiterTM plate and surrounded by enzyme beads (sulfurylase and luciferase). The light signals were represented in flow grams and analyzed; a nucleotide sequence was determined for each read with the GS Amplicon Variant Analyzer software.

### Bioinformatic Analysis

Data were analyzed using a custom bioinformatic pipeline as fully described in Massa et al. (2020). Raw sequence reads were demultiplexed. The reads with the following characteristics were discarded: (1) read length less than 200 nucleotides, (2) average Phred quality score less than 25 (Ewing et al., 1998), and (3) presence of at least one ambiguous base inside the read. Then, an alignment of each sequence was performed against our AMF LSU rDNA database, consisting of 3.803 univocal sequences downloaded from online sources: EBI and SILVA databases, and from the web site^1^ (Krüger et al., 2011). Our database was prepared as described in Massa et al. (2020). The alignment of each sequence was performed using BLASTN (Altschul et al., 1997). Two criteria were applied in order to identify the taxa at species level (named “known”): coverage ≥80% and similarity of sequences ≥97% according to Lindahl et al. (2013) and Hart et al. (2015). Following these criteria, chimeras were also removed. All the sequences that did not satisfy both afore-mentioned criteria were then aligned against themselves. After comparing one sequence to each other, all those with coverage ≥80% and similarity of sequences ≥97% were grouped together, and each group was named *de novo* (Massa et al., 2020).

Bioinformatic analysis was performed on a database containing the results normalized at 8,000 sequences. The rarefaction curves were plotted with the RAM package of R (R Core Team., 2018; Supplementary Figure 1).

### Taxa Abundance and Biodiversity Analysis

In order to describe the distribution of “known” and *de novo* taxa in the different samples, the number of taxa with at least 10 sequences in one replicate was calculated, considering altogether the replicates for each sample (if the same taxon was present more than one time in the different replicates of the sample, it was counted as one). The freely available Venny version 2.1 software^2^ was used to construct the Venn diagrams of AMF taxa in Bs and Rs soils.

For abundance analysis, only taxa present with at least 10 sequences in one replicate were used (“known” reported in Supplementary Table 1 and *de novo* in Supplementary Table 2). Then, the *de novo* taxa were BLASTed against the NCBI database to give them a name (Supplementary Table 2) as fully described in Massa et al. (2020).

Using these data, the four AMF communities were compared also by analysis with MicrobiomeAnalyst, a freely available online software^3^, according to Berlanas et al. (2019) and to Sergaki et al. (2018), which allow community description by alpha diversity, heat trees, beta diversity, and linear discriminant analysis effect size (LDA-LEfSe).

In particular, alpha diversity analysis was performed using the phyloseq package (McMurdie and Holmes, 2013). The results were represented as box plots for each sample. The statistical significance was also estimated using either parametric or non-parametric tests.

Heat tree method was used to compare abundance at the species taxonomic level for space and time factors. Heat tree uses a hierarchical structure of taxonomic classifications to quantitatively (median abundance) and statistically (non-parametric Wilcoxon rank-sum test) describe taxon differences among communities. The resulting differential heat tree shows the relative abundance of each taxon in two different samples. Heat tree analysis was performed using R metacoder package (Foster et al., 2017).

Beta diversity was analyzed using the phyloseq package (McMurdie and Holmes, 2013). Principal coordinate analysis (PCoA) was applied using Bray–Curtis distance-based method. Permutational ANOVA (PERMANOVA) was employed for the evaluation of the statistical significance of the clustering pattern in ordination plots.

Moreover, LDA-LEfSe analysis using the non-parametric factorial Kruskal–Wallis sum-rank test was applied. Features were considered significant for adjusted *p*-value cutoff at 0.05 and LDA score at 1.0.

Finally, for each sample, considering the sequence abundance in the single replicates, the median value of the number of sequences was calculated, and only the taxa with a median higher than 0 (yellow lines in Supplementary Tables 1, 2) were considered to be assigned to the different taxonomic groups.

### Data Availability

The genomic datasets are available in NCBI using BioProject ID PRJNA613620 containing the following BioSamples: SAMN14411203, SAMN14411449, SAMN14411451, and SAMN14411452 (project name: *Vitis vinifera* association with local AMF communities in an Italian vineyard at two different phenological stages).

## Results

### AMF Root Colonization

Arbuscular mycorrhizal fungi root colonization was checked in grapevine plants in both sampling times. The frequencies of colonization were 92.0 ± 2.5% at the first sampling and 94.2 ± 3.3% at the second sampling (*p*-value = 0.3658). The degrees of mycorrhizal colonization were 35.4 ± 5.5 and 44.5 ± 8.0% (*p*-value = 0.3379), the arbuscule abundances were 13.9 ± 3.6 and 16.8 ± 5.3% (*p* = 0.6547), and the vesicle abundances were 11.3 ± 3.0 and 17.6 ± 7.7% (*p* = 0.9491) in the first and the second sampling, respectively. No significant differences between the two sampling times were detected in all the considered parameters.

### Taxa Abundance and Analysis of Biodiversity

**FIGURE 2:**
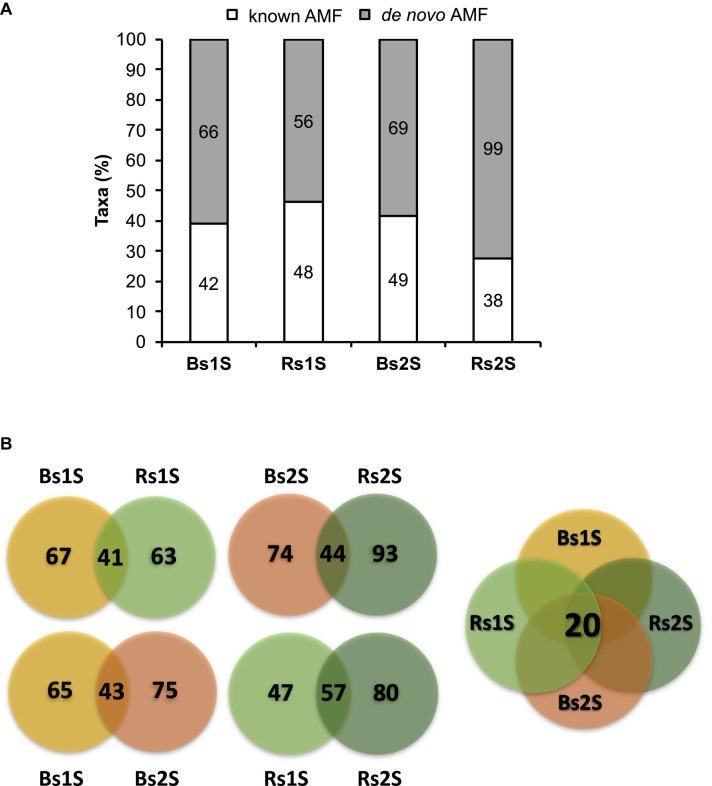
**(A)** Abundance of the taxa obtained from the soils associated (Rs) or not (Bs) with the roots of *Vitis vinifera* cv. Pinot Noir during the two sampling times (1S = flowering and 2S = fruit development). Bars represent the percentage of known (white) or *de novo* (gray) arbuscular mycorrhizal fungi taxa, while labels inside the bars indicate the actual number of taxa. **(B)** Venn diagrams showing the number of taxa that were exclusive or common to (a) Bs and Rs soils in the first (1S) or in the second (2S) sampling time (upper part on the left of the figure), (b) the first (1S) and the second (2S) sampling times in Bs or in Rs (lower part on the left of the figure), and (c) the four soil samples (Bs1S, Rs1S, Bs2S, and Rs2S—on the right of the figure). The Venn diagrams were calculated by the freely available Venny version 2.1 software (http://bioinfogp.cnb.csic.es/tools/venny).

Table 1 shows the real number of sequences for each replicate of Bs and Rs soils. On average, the number of obtained sequences was about 9,000. As the rarefaction curves reached a plateau (Supplementary Figure 1), the number of obtained sequences was adequate to properly describe the biodiversity of the AMF community in the samples. A total of 467 taxa (305 univocal taxa) were obtained from the two soils at the two sampling times, including 177 (87 univocal taxa) “known AMF” and 290 (218 univocal taxa) *de novo* taxa (Supplementary Tables 1, 2). Figure 2A shows the distribution of taxa in the different samples. In Bs soil, 108 and 118 taxa were obtained in the first and the second sampling time, respectively. In particular, 42 “known AMF” taxa occurred in Bs1S sample and 49 in Bs2S (factor time), while the number of *de novo* AMF taxa at the two sampling times was 66 and 69, respectively. In Rs soil, a total of 104 and 137 taxa was found in the first and the second sampling time, respectively. In particular, in this soil, 48 “known AMF” taxa were observed at the first sampling time while 38 taxa in the second one; on the contrary, the number of *de novo* AMF taxa was 56 and 99, respectively (factor time).

**TABLE 1:** Number of sequences obtained from the different replicates of the soils associated (Rs) or not (Bs) with the roots of *Vitis vinifera* cv. Pinot Noir at the two sampling times (1S = flowering; 2S = fruit development).

Soil sample	Replicate	Number of sequences		Average number of sequences
		1S	2S	
Bs	1	2,492	9,670	
	2	10,949	9,730	
	3	6,252	11,639	9,133
	4	9,685	12,169	
	5	11,600	7,145	
Rs	1	6,286	8,153	
	2	13,603	6,405	
	3	9,910	6,623	9,224
	4	10,229	12,570	
	5	9,641	8,816	

Bs1S and Rs1S or Bs2S and Rs2S (factor space) shared 41 and 44 taxa, respectively (Figure 2B and Supplementary Table 3). The AMF communities in Bs soils at the two sampling times showed 43 taxa in common. On the other hand, the number of taxa shared between the two sampling times in Rs soil was 57. Finally, all samples had 20 taxa in common (Figure 2B and Supplementary Table 3).

**FIGURE 3:**
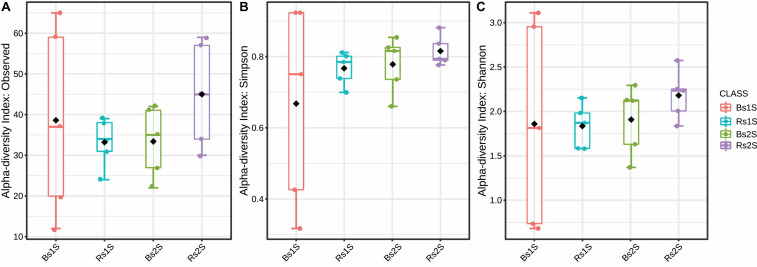
Alpha diversity indices. **(A)** Number of observed arbuscular mycorrhizal fungi species (*p*-value, 0.5393), **(B)** Simpson index (*p*-value, 0.4700), and **(C)** Shannon’s index (*p*-value, 0.8177) of biodiversity detected in the soils associated (Rs) or not (Bs) with the roots of *Vitis vinifera* cv. Pinot Noir at the two sampling times (1S = flowering and 2S = fruit development). Alpha diversity analysis was performed using the phyloseq package of MicrobiomeAnalyst, a freely available online software (https://www.microbiomeanalyst.ca).

To compare AMF alpha diversity, the number of observed species, Simpson and Shannon indices were calculated (Figure 3). For all these indices, differences were not significant, even if an increased number of observed species occurred in Rs2S compared to the other samples.

**FIGURE 4:**
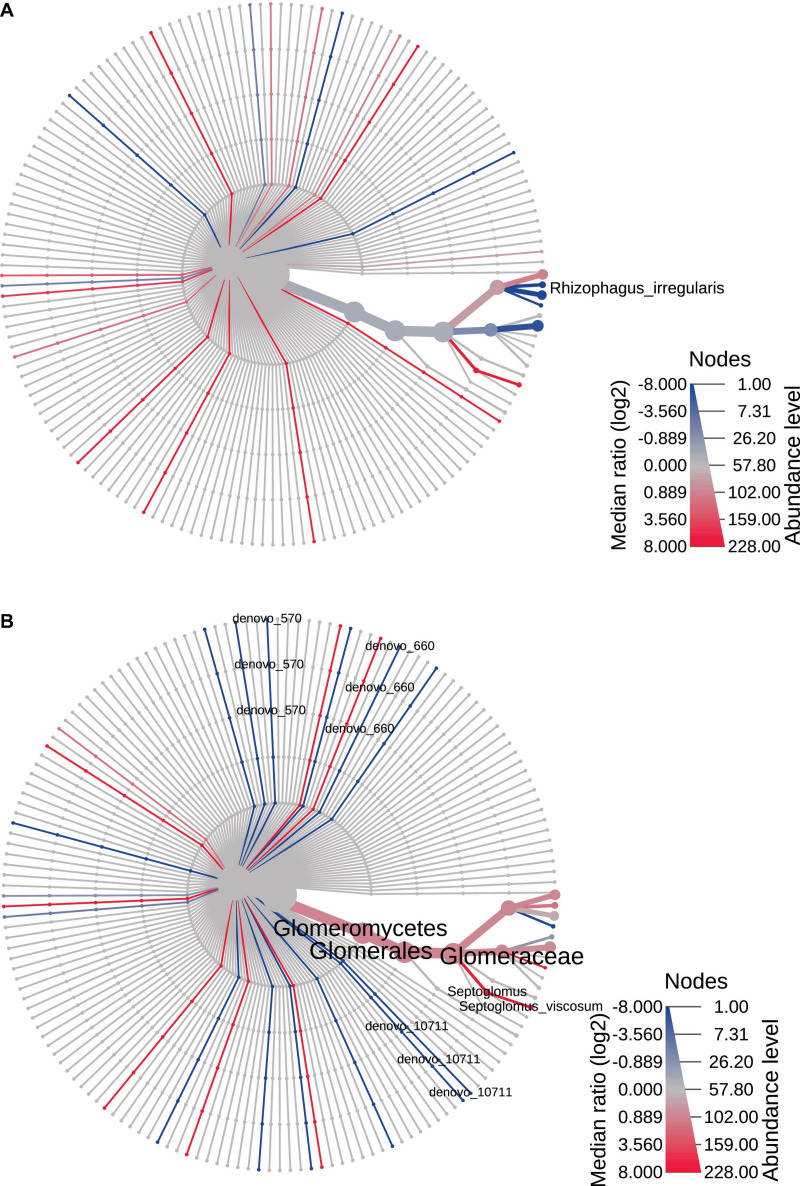
Heat tree based on the factor “time”, reporting the effect of sampling time on the hierarchical structure of taxonomic classifications (median abundance, non-parametric Wilcoxon rank–sum test). **(A)** Bs1S vs. Bs2S. **(B)** Rs1S vs. Rs2S. Heat tree analysis was performed using the R metacoder package of MicrobiomeAnalyst, a free available on-line software (https://www.microbiomeanalyst.ca).

**FIGURE 5:**
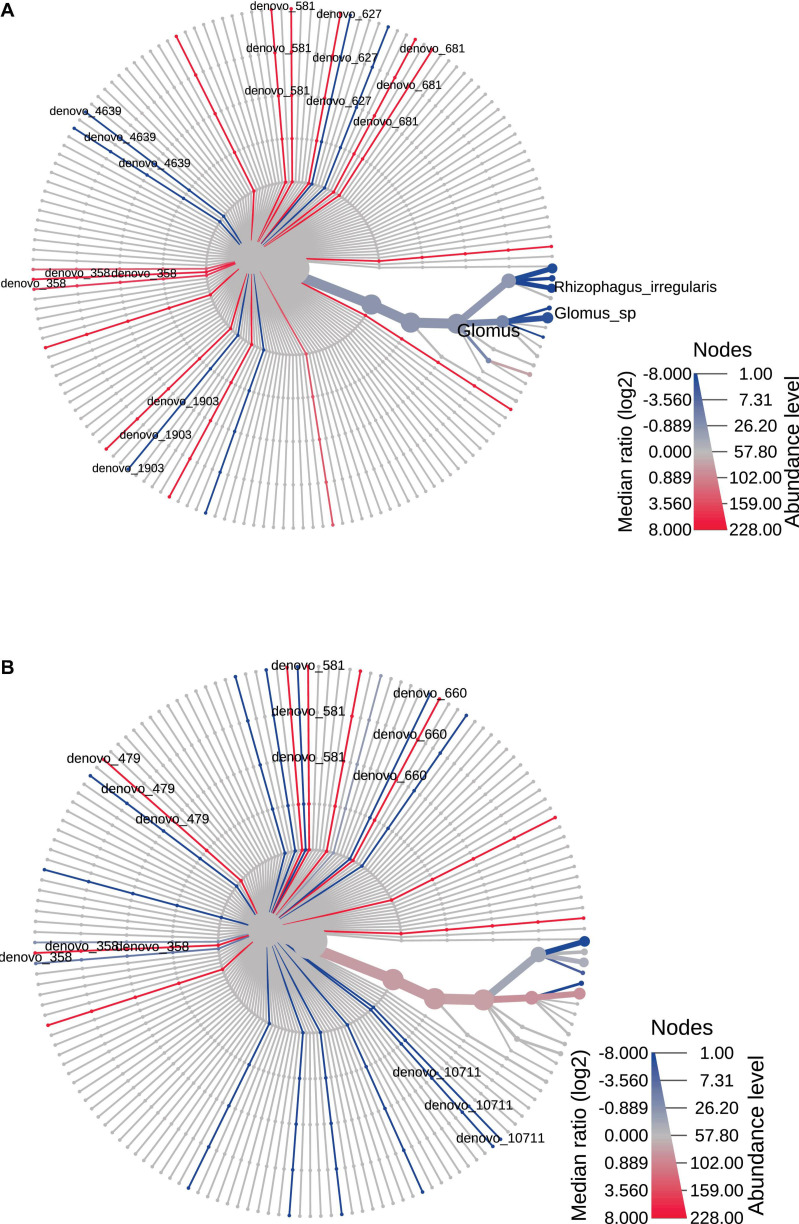
Heat tree based on the factor “space” reporting the effect of the presence of *Vitis vinifera* roots on the hierarchical structure of taxonomic classifications (median abundance, non-parametric Wilcoxon rank–sum test). **(A)** Bs1S vs. Rs1S. **(B)** Bs2S vs. Rs2S. Heat tree analysis was performed using the R metacoder package of MicrobiomeAnalyst, a freely available online software (https://www.microbiomeanalyst.ca).

The heat trees reported in Figures 4, 5 represented time and space effect on the AMF community. In particular, Figure 4A is relative to time effect in Bs and displays the increased (blue line) abundance of *Rhizophagus irregularis* in Bs2S compared to Bs1S (Supplementary Table 4). Figure 4B reports the time effect in the soil associated to *V. vinifera* roots and shows the increase (blue lines) of *de novo*_570 (uncultured *Glomus*), *de novo*_660 (*R. irregularis*), and *de novo*_10711 (uncultured *Glomus*) and the decrease (red line) of *Septoglomus viscosum* in the Rs2S compared to Rs1S samples (Supplementary Table 4). In Figure 5A, is represented the heat tree related to the space effect in the first sampling time: while *Glomus* sp., *Rhizophagus irregularis*, *de novo*_1903 (*R. irregularis*), and *de novo*_4639 (*Glomus* sp.) increased (blue lines) in Rs1S compared to Bs1S soil, *de novo*_358 (uncultured *Archaeospora*), *de novo*_581 (uncultured *Archaeospora*), *de novo*_627 (uncultured *Archaeospora*), and *de novo*_681 (uncultured *Archaeospora*) decreased (red lines) (Supplementary Table 4). Finally, Figure 5B shows the heat tree related to the space effect in the second sampling time. Although *de novo*_660 (*R. irregularis*) and *de novo*_10711 (uncultured *Glomus*) increased (blue lines) in Rs2S compared to Bs2S soil, *de novo*_358 (uncultured *Archaeospora*), *de novo*_479 (uncultured *Glomerales*), and *de novo*_581 (uncultured *Archaeospora*) decreased (red lines) (Supplementary Table 4).

**FIGURE 6:**
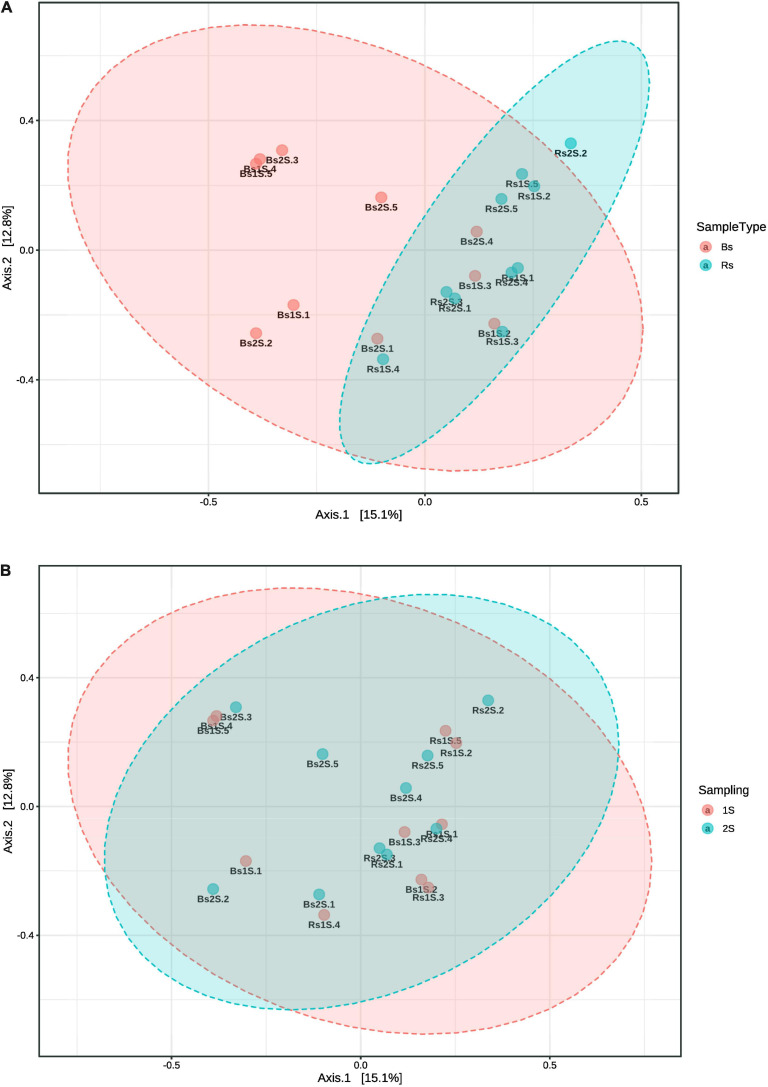
Comparison by principal coordinate analysis of the ecological distance (based on Bray–Curtis distance method) of the different compartments. **(A)** Soil effect (Bs = soil not associated to grapevine roots, Rs = soil associated with the roots of *Vitis vinifera* cv. Pinot Noir); PERMANOVA *F*-value: 1.787, *R*-square: 0.090312, and *p*-value < 0.005. **(B)** Sampling time effect (1S = flowering and 2S = fruit development); PERMANOVA *F*-value: 0.48119, *R*-square: 0.026037, and *p*-value < 0.994. Beta diversity analysis was performed using the phyloseq package of MicrobiomeAnalyst, a freely available online software (https://www.microbiomeanalyst.ca).

Data analyzed by PCoA underlined a different community composition between the two soils (PERMANOVA *p*-value, <0.005), with Rs samples distributed more homogeneously than Bs ones at both sampling times (Figure 6A), but no difference occurred between the two sampling times (PERMANOVA *p*-value, 0.994) (Figure 6B).

**FIGURE 7:**
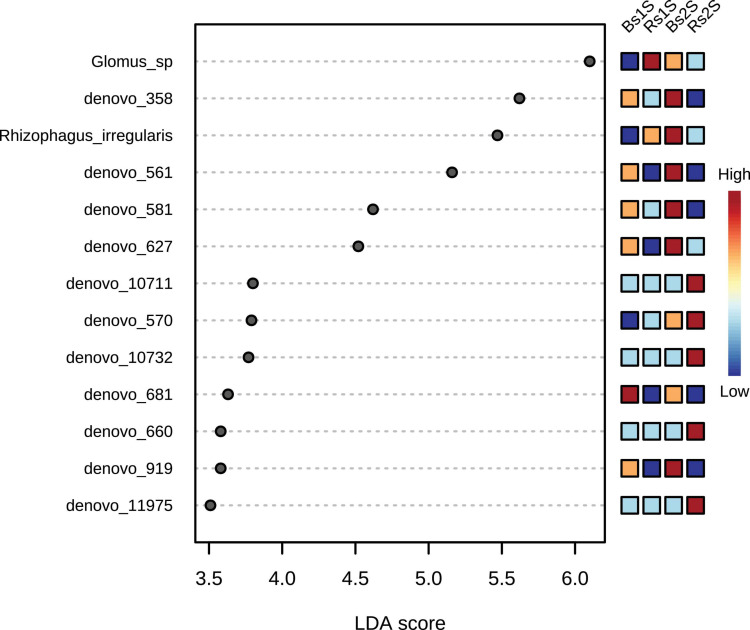
Linear discriminant analysis–effect size (LDA–LEfSe) results using non-parametric factorial Kruskal–Wallis sum–rank test. Adjusted *p*-value cutoff = 0.05 and LDA score = 1.0; *Glomus* sp. (LDA score 6.1); *de novo_*358 – uncultured *Archaeospora* (LDA score 5.62), *Rhizophagus irregularis* (LDA score 5.47), *de novo_*561 – uncultured *Archaeospora* (LDA score 5.16), *de novo_*581 – uncultured *Archaeospora* (LDA score 4.62), *de novo_*627 – uncultured *Archaeospora* (LDA score 4.52), *de novo_*10711 – uncultured *Glomus* (LDA score 3.8), *de novo_*570 – uncultured *Glomus* (LDA score 3.79), *de novo_*10732 – *Rhizophagus intraradices* (LDA score 3.77), *de novo_*681 – uncultured *Archaeospora* (LDA score 3.63), *de novo_*660 – *Rhizophagus irregularis* (LDA score 3.58), *de novo_*919 – uncultured *Archaeospora* (LDA score 3.58), and *de novo_*11975 – uncultured *Rhizophagus* (LDA score 3.51). LEfSe analysis was performed with MicrobiomeAnalyst, a freely available online software (https://www.microbiomeanalyst.ca).

The LEfSe results, presented in Figure 7 and Supplementary Table 5, showed the 13 taxa that better explained the differences in the AMF community analyzed. In particular, *Glomus* sp. showed a LDA score of 6.1 (*p*-value, 0.033341), the highest values in Rs1S followed by Bs2S. Other important taxa present in the soil associated to *V. vinifera* roots (Rs) were *de novo_*10711 that was uncultured *Glomus* (LDA score, 3.8), *de novo_*570 that was uncultured *Glomus* (LDA score, 3.79), *de novo_*10732 that was *Rhizophagus intraradices* (LDA score, 3.77), *de novo_*660 that was *R. irregularis* (LDA score, 3.58), and *de novo_*11975 that was uncultured *Rhizophagus* (LDA score, 3.51). On the contrary, the seven *de novo* taxa – *de novo_*358 that was uncultured *Archaeospora* (LDA score, 5.62), *R. irregularis* (LDA score, 5.47), *de novo_*561 that was uncultured *Archaeospora* (LDA score, 5.16), *de novo_*581 that was uncultured *Archaeospora* (LDA score, 4.62), *de novo_*627 that was uncultured *Archaeospora* (LDA score, 4.52), *de novo_*681 that was uncultured *Archaeospora* (LDA score, 3.63), and *de novo_*919 that was uncultured *Archaeospora* (LDA score, 3.58) – mostly explained the differences in Bs soil.

**FIGURE 8:**
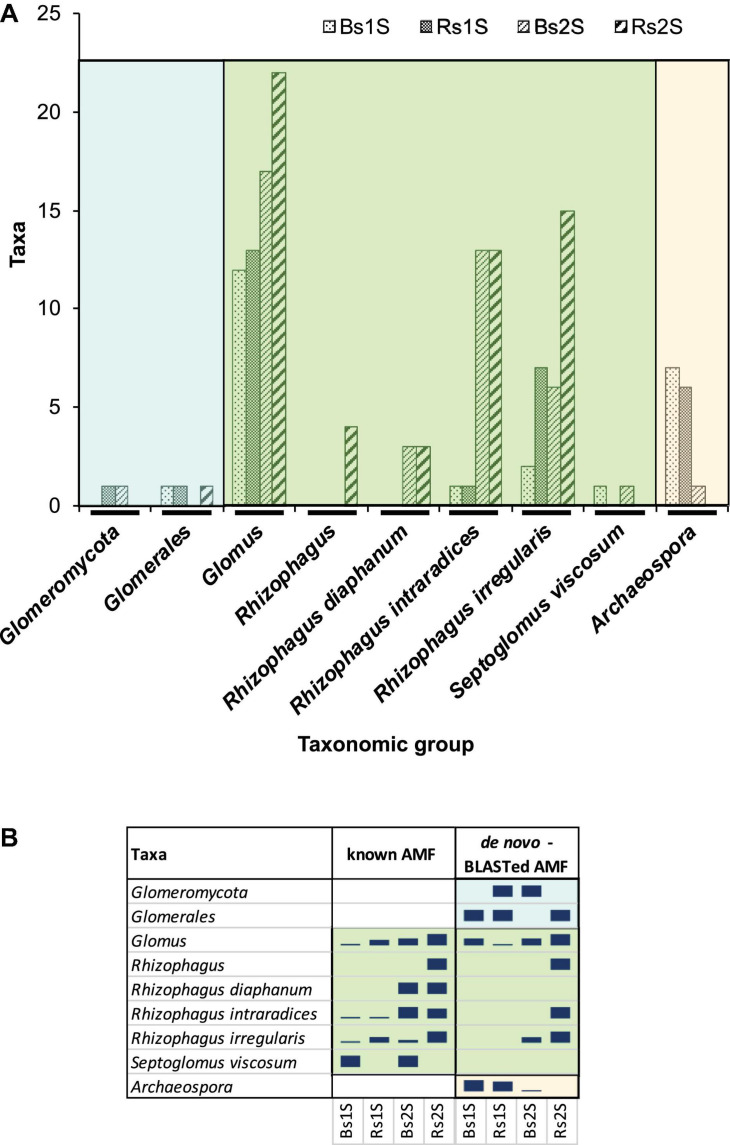
**(A)** Number of taxa belonging to the different taxonomic groups – obtained by adding the “*de novo*-BLASTed AMF” taxa with the “known AMF” ones on the basis of the group to which they belonged – in the different samples (Bs = soil not associated to grapevine roots, Rs = soil associated with the roots of *Vitis vinifera* cv. Pinot Noir) at the two sampling times (1S = flowering and 2S = fruit development). **(B)** Relative abundance of taxa obtained considering separately “known AMF” and “*de novo*-BLASTed AMF”, belonging to each taxonomic group, in Bs and Rs soils at the two sampling times (1S = flowering and 2S = fruit development).

The *de novo* AMF taxa were named after being BLASTed against NCBI database (Supplementary Table 2). Then, for each sample, all the “known AMF” (yellow lines in Supplementary Table 1) and the *de novo*-BLASTed (yellow lines in Supplementary Table 2) taxa belonging to the same taxonomic group were added (Figure 8A). Two taxa corresponded to higher AMF classification levels (subphylum and order; Figure 8A – cyan area on the left). Many taxa were included in the family Glomeraceae (Figure 8A – central green area); the genus *Glomus* was the most abundant group (22 taxa in Rs2S). All the other remaining taxa of the family Glomeraceae belonged to the genus *Rhizophagus*, with the exception of *S. viscosum*. All the taxa belonging to the family Archaeosporaceae were included in only one genus (*Archaeospora*; Figure 8A – orange area on the right).

Most of the taxonomic groups were detected in both soils, with the exception of unidentified species of the genus *Rhizophagus* that were observed only in Rs2S and *S. viscosum* that was detected only in Bs soils at both sampling times (Figure 8A). Moreover, the species *Rhizophagus diaphanum* was present in both soils, but only at the second sampling time (Figure 8A).

The *de novo* AMF taxon identification by NCBI permitted to highlight the presence of the genus *Archaeospora* that did not appear among the “known AMF” taxa (Figure 8B). In fact, the “known AMF” taxa belonged only to the family Glomeraceae (Figure 8B).

All the identified *de novo* AMF taxa corresponded to uncultured AMF, with the exception of four taxa: one belonging to *Glomeromycota*, five to the genus *Glomus*, three to the species *R. irregularis*, and one to the species *R. intraradices* (Supplementary Table 2).

## Discussion

Arbuscular mycorrhizal fungi are widespread symbionts able to colonize the roots of a lot of terrestrial plant species, including *V. vinifera* (Trouvelot et al., 2015). The characterization of AMF communities associated to grapevines is of interest by both economic and historical viewpoints. In fact, Piedmont is one the most important Italian regions for vineyard cultivation and wine production (ISTAT, 2018), and since 2014, the UNESCO World Heritage list has included the hills of the Piedmont area covering the Langhe, Roero, and Monferrato^4^. Therefore, we focused our attention on the AMF community associated to the Pinot Noir grapevine cultivar, which is largely cultivated in Piedmont.

At the beginning, the mycorrhizal colonization of grapevine roots was evaluated to assess the actual interaction between the plant and the AM fungi present in the soil. The plant roots were colonized by AMF at levels that were similar to those reported in the literature (Schreiner, 2005, 2020; Likar et al., 2013; Bouffaud et al., 2016; Massa et al., 2020), and no significant differences between the two sampling times were observed. This is in contrast with the findings of Schreiner (2005) who reported that the mycorrhizal colonization of *V. vinifera* cv. Pinot Noir roots increased before bud break in the spring, reaching values of about 50–60% of root length in early summer, that remained constant until leaf senescence in late fall.

Fragments resulting from pyrosequencing analysis were 700 bp in length, so they have a larger size if compared to those obtained in previously published works (Lumini et al., 2010; Holland et al., 2014). A total of 467 taxa have been found. This result is consistent with that reported by Holland et al. (2016), in which 816 taxa were obtained using the SSU rDNA marker for studying the AMF biodiversity in different vineyards in Canada. Similarly, Massa et al. (2020) found 528 taxa of AMF in an integrated pest-managed (IPM) vineyard in Piedmont (Italy). Considering the distribution of taxa among the different samples, the highest number of taxa was observed in Rs2S. The two soils showed different taxa: in the second sampling, only 44 taxa were shared by both soils, while 74 were present only in Bs2S and 93 only in Rs2S. Moreover, only 20 taxa were common to the two soils at the two sampling times. Compared to the AMF community characterization performed in the IPM vineyard considered in Massa et al. (2020), in the present work, focused on a conventionally managed vineyard, a higher number of taxa shared between the two soils and a lower number of taxa exclusive of each soil were observed at the two sampling times. On the contrary, considering the sampling time, both Bs and Rs soils shared numbers of taxa similar to those reported in Massa et al. (2020). However, a lower number of exclusive taxa for each sampling time was observed. The differences between the AMF community described in these two studies could be due to the different vineyard management (IPM vs. conventional), but the impact of chemical–physical soil parameters cannot be ruled out.

The biodiversity indices (number of observed species – Shannon and Simpson’s indices) did not show significant differences according to soil type (Bs vs. Rs) and sampling time (1S vs. 2S). Our results confirmed what was previously reported by Schreiner and Mihara (2009), demonstrating that AMF vineyard communities did not change with season succession, but differed according to the vineyard age and type of soil. Consistently, the impact of seasonality on the biodiversity of AMF has been described in crops other than grapevines (Dumbrell et al., 2011; Bouamri et al., 2014; Vargas-Gastélum et al., 2015).

Going into deeper detail and considering time and space as separate factors, some significant differences have been highlighted. In particular, an increase of *R. irregularis* in Bs and an increase of *R. irregularis* and uncultured *Glomus*, combined with a reduction of *S. viscosum* in Rs, were observed in the second sampling time compared to the first one. Moreover, in both sampling times, an increase of *Glomus* and *R. irregularis* and a decrease in uncultured *Archaeospora* were recorded in Rs compared to Bs.

Finally, the phylogenetic distance obtained by PCoA supported the difference between Bs and Rs soils, confirming what was previously discussed. These data are in accordance with what was reported in other scientific works, which demonstrated the effect of the plant in selecting the associated AM fungal population (Cesaro et al., 2008; Holland et al., 2014).

Consistently with Massa et al. (2020), no fungal sequences belonging to Claroideoglomeraceae, Acaulosporaceae, Gigasporaceae, and Diversisporaceae were detected even if the primers used were specific for all AMF. Many fungal taxa corresponded to Glomeraceae, which is one of the most represented family in agricultural lands (Cesaro et al., 2008; Berruti et al., 2014) and also in vineyards (Balestrini et al., 2010; Lumini et al., 2010; Holland et al., 2014; Trouvelot et al., 2015; Bouffaud et al., 2016; Massa et al., 2020). This abundance could be explained by the high growth rate and the fast recovery of the hyphal network following the disturbance caused by agricultural practices (Berruti et al., 2014; Trouvelot et al., 2015) that are typical of the fungi belonging to this family. Although the genus *Glomus* was the most abundant, all the other remaining taxa of the family Glomeraceae, with the exception of *S. viscosum*, belonged to the genus *Rhizophagus*.

In agreement with Schreiner and Mihara (2009); Balestrini et al. (2010), Oehl and Koch (2018); Massa et al. (2020), and Schreiner (2020), we found AMF members of the family Archaeosporaceae represented only by one genus, *Archaeospora*.

The AMF community of a conventionally managed vineyard (with a prevalence of Glomeraceae and Archaeosporaceae) described in this work partly overlapped with those characterized in other vineyards subjected to a different management (Schreiner and Mihara, 2009; Balestrini et al., 2010; Holland et al., 2016; Massa et al., 2020). However, a lot of uncontrolled variables such as soil tillage, cover crops, manure application, and quality and amount of herbicides, fertilizers, and pesticides can influence the AMF diversity or community composition (Turrini et al., 2017).

In conclusion, in this work, a difference in AMF communities was observed between the two considered soils (Bs and Rs) independently from the plant phenological stage, suggesting a possible role of *V. vinifera* in modulating the AMF populations associated to its roots.

Overall, looking at the improvement in the global sustainability of viticulture practices, this study broadens the knowledge already gained by other works (Novello et al., 2017; Bona et al., 2019; Gamalero et al., 2020; Massa et al., 2020) regarding the microbiota associated with Pinot Noir grapevines cultivated in a geographic region historically dedicated to viticulture.

## Data Availability Statement

The datasets presented in this study can be found in online repositories. The names of the repository/repositories and accession number(s) can be found below: https://www.ncbi.nlm.nih.gov/, PRJNA613620.

## Author Contributions

PC cooperated in biological experiments, performed sample preparation for pyrosequencing, prepared the figures, analyzed data, and wrote the manuscript. NM organized the sampling, cooperated to data elaboration, prepared the figures, and wrote the manuscript. EB wrote the manuscript. GN participated to the sampling and performed DNA extraction. VT, EG, and GB cooperated in manuscript writing. LB performed pyrosequencing and bioinformatic analyses. FM cooperated in bioinformatic analyses. GL coordinated biological experiments, bioinformatic analyses, and manuscript writing. All authors revised the manuscript.

## Conflict of Interest

LB and FM were employed by the company SmartSeq s.r.l. The remaining authors declare that the research was conducted in the absence of any commercial or financial relationships that could be construed as a potential conflict of interest.

## Footnotes

1 http://www.amf-phylogeny.com/amphylo_species.html

2 www://bioinfogp.cnb.csic.es/tools/venny

3 https://www.microbiomeanalyst.ca

4 http://whc.unesco.org/en/list/1390

## Supplementary Material

Supplementary Figure 1 Rarefaction curves.

Supplementary Table 1 List of IDs for “known” taxa identified by large subunit rDNA database.

Supplementary Table 2 Identification of *de novo* taxa by BLAST analysis against the NCBI database.

Supplementary Table 3 List of the IDs of “known” taxa (identified by large subunit rDNA database) and *de novo* taxa that were common between different soils (Bs or Rs) and in different sampling times (1S or 2S).

Supplementary Table 4 Heat tree difference table reporting the results (*p*-values) of the non-parametric Wilcoxon rank–sum test used to depict the taxonomic differences between AMF communities.

Supplementary Table 5 Linear discriminant analysis effect size results using non-parametric factorial Kruskal–Wallis sum–rank test.
