# Supplementary material for: Native AMF Communities in an Italian Vineyard at Two Different Phenological Stages of Vitis vinifera
Source: Front Microbiol. 2021 Jul 19;12:676610. doi: 10.3389/fmicb.2021.676610 (PMC8326575; doi:10.3389/fmicb.2021.676610)
Supplement: Supplementary Figure 1 — Rarefaction curves. [file Data_Sheet_1.PDF]

Native AMF communities in an Italian vineyard at two different phenological stages of *Vitis vinifera*

Cesaro P.[1], Massa N.[1*], Bona E.[2], Novello G.[1], Todeschini V.[2], Boatti L.[3], Mignone F.[1,3], Gamalero E.[1], Berta G.[1], Lingua G.[1]

[1] Università del Piemonte Orientale, Dipartimento di Scienze e Innovazione Tecnologica, Viale T. Michel 11, Alessandria 15121, Italy
[2] Università del Piemonte Orientale, Dipartimento di Scienze e Innovazione Tecnologica, Piazza San Eusebio 5, 13100 Vercelli, Italy
[3] SmartSeq s.r.l., spin-off of the Università del Piemonte Orientale, Viale T. Michel 11, Alessandria 15121, Italy

*** Corresponding author:**
Dipartimento di Scienze e Innovazione Tecnologica, Università del Piemonte Orientale,
Viale T. Michel 11, Alessandria 15121, Italy
Tel: 0131 360 231
Fax: 0131 360 243
E-mail: nadia.massa@uniupo.it

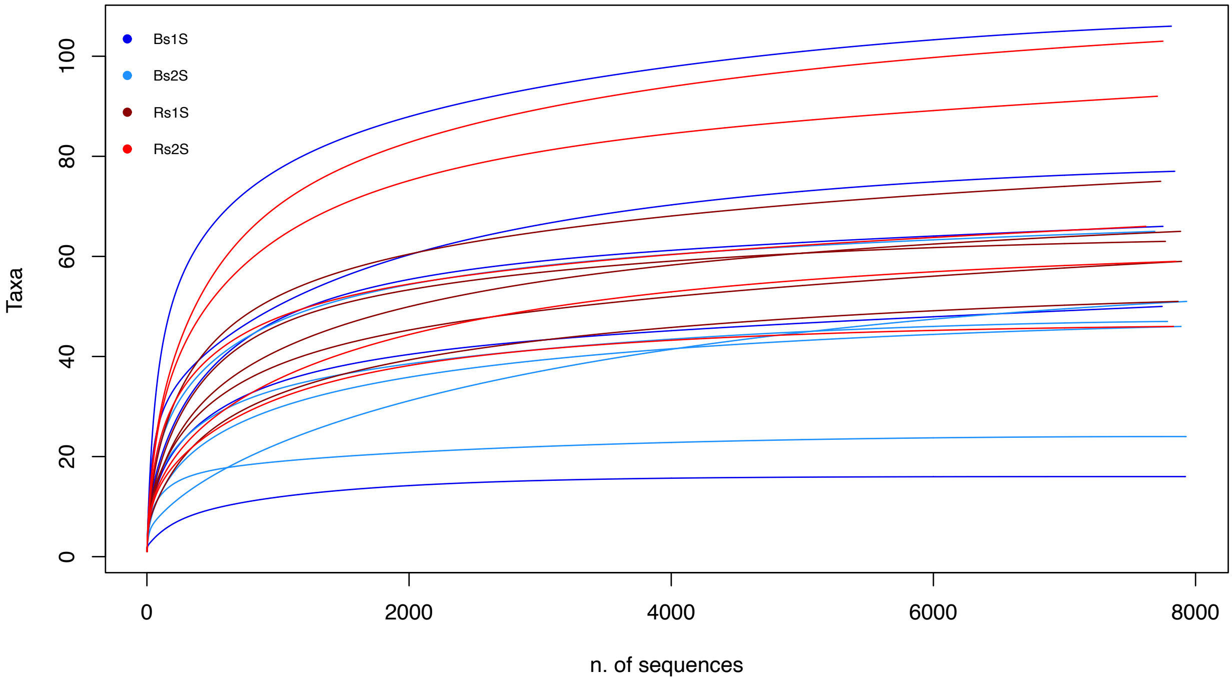

Figure S1. Rarefaction curves.

Table S1. List of IDs for "known" taxa identified by LSU rDNA database.
Highlighted in yellow the taxa with median abundance higher than 0.

Taxa ID	Identification by LSU rDNA database	Description
AF145743	*Glomus geosporum* strain BEG 106 large subunit ribosomal RNA gene, partial sequence	*Funneliformis geosporum*
AJ854588	*Glomus* sp. MUCL 43203 28S rRNA gene, strain MUCL 43203, clone 5	*Rhizophagus* sp.
AJ854594	*Glomus* sp. MUCL 43204 28S rRNA gene, strain MUCL 43204, clone 1	*Glomus* sp.
AJ854601	*Glomus* sp. MUCL 43205 28S rRNA gene, strain MUCL 43205, clone 1	*Glomus* sp.
AJ854602	*Glomus* sp. MUCL 43205 28S rRNA gene, strain MUCL 43205, clone 2	*Glomus* sp.
AJ854606	*Glomus* sp. MUCL 43205 28S rRNA gene, strain MUCL 43205, clone 6	*Glomus* sp.
AJ854609	*Glomus* sp. MUCL 43205 28S rRNA gene, strain MUCL 43205, clone 9	*Glomus* sp.
AJ854611	*Glomus* sp. MUCL 43205 28S rRNA gene, strain MUCL 43205, clone 11	*Glomus* sp.
AJ854614	*Glomus* sp. MUCL 43206 28S rRNA gene, strain MUCL 43206, clone 2	*Glomus* sp.
AJ854615	*Glomus* sp. MUCL 43206 28S rRNA gene, strain MUCL 43206, clone 3	*Glomus* sp.
AJ854616	*Glomus* sp. MUCL 43206 28S rRNA gene, strain MUCL 43206, clone 4	*Glomus* sp.
AJ854618	*Glomus* sp. MUCL 43206 28S rRNA gene, strain MUCL 43206, clone 6	*Glomus* sp.
AJ854619	*Glomus* sp. MUCL 43206 28S rRNA gene, strain MUCL 43206, clone 7	*Glomus* sp.
AJ854620	*Glomus* sp. MUCL 43206 28S rRNA gene, strain MUCL 43206, clone 8	*Glomus* sp.
AJ854621	*Glomus* sp. MUCL 43206 28S rRNA gene, strain MUCL 43206, clone 9	*Glomus* sp.
AJ854622	*Glomus* sp. MUCL 43206 28S rRNA gene, strain MUCL 43206, clone 10	*Glomus* sp.
AJ854623	*Glomus* sp. MUCL 43207 28S rRNA gene, strain MUCL 43207, clone 1	*Glomus* sp.
AJ854625	*Glomus* sp. MUCL 43207 28S rRNA gene, strain MUCL 43207, clone 3	*Glomus* sp.
AJ854626	*Glomus* sp. MUCL 43207 28S rRNA gene, strain MUCL 43207, clone 4	*Glomus* sp.
AJ854629	*Glomus* sp. MUCL 43207 28S rRNA gene, strain MUCL 43207, clone 7	*Glomus* sp.
AY639205	*Rhizophagus* cf. *intraradices* HG-2010 clone 34.1 28S large subunit ribosomal RNA gene, partial sequence	*Rhizophagus intraradices*
AY639208	*Rhizophagus* cf. *intraradices* HG-2010 clone 42.2 28S large subunit ribosomal RNA gene, partial sequence	*Rhizophagus intraradices*
AY639214	*Rhizophagus* cf. *intraradices* HG-2010 isolate 57 clone 57.7.1 28S large subunit ribosomal RNA gene, partial sequence	*Rhizophagus intraradices*
AY639216	*Rhizophagus* cf. *intraradices* HG-2010 isolate 58 clone 58.1.4 28S large subunit ribosomal RNA gene, partial sequence	*Rhizophagus intraradices*
AY639218	*Rhizophagus* cf. *intraradices* HG-2010 isolate 107 clone 107.3.1 28S large subunit ribosomal RNA gene, partial sequence	*Rhizophagus intraradices*
AY639219	*Rhizophagus* cf. *intraradices* HG-2010 isolate 107 clone 107.3.3 28S large subunit ribosomal RNA gene, partial sequence	*Rhizophagus intraradices*
AY639221	*Rhizophagus* cf. *intraradices* HG-2010 isolate 107 clone 107.6.2 28S large subunit ribosomal RNA gene, partial sequence	*Rhizophagus intraradices*
AY639294	*Rhizophagus* cf. *intraradices* HG-2010 clone 28.1 28S large subunit ribosomal RNA gene, partial sequence	*Rhizophagus intraradices*
AY639298	*Rhizophagus* cf. *intraradices* HG-2010 clone 35.2 28S large subunit ribosomal RNA gene, partial sequence	*Rhizophagus intraradices*
AY639299	*Rhizophagus* cf. *intraradices* HG-2010 isolate 57 clone 57.6.3 28S large subunit ribosomal RNA gene, partial sequence	*Rhizophagus intraradices*
AY639300	*Rhizophagus* cf. *intraradices* HG-2010 isolate 57 clone 57.7.2 28S large subunit ribosomal RNA gene, partial sequence	*Rhizophagus intraradices*
AY639301	*Rhizophagus* cf. *intraradices* HG-2010 isolate 57 clone 57.7.3 28S large subunit ribosomal RNA gene, partial sequence	*Rhizophagus intraradices*
AY639303	*Rhizophagus* cf. *intraradices* HG-2010 isolate 58 clone 58.1.3 28S large subunit ribosomal RNA gene, partial sequence	*Rhizophagus intraradices*
DQ469113	*Glomus* cf. *diaphanum* 589 clone 589.1 28S large subunit ribosomal RNA gene, partial sequence	*Glomus diaphanum*
DQ469114	*Glomus* cf. *diaphanum* 589 clone 589.2 28S large subunit ribosomal RNA gene, partial sequence	*Glomus diaphanum*
DQ469115	*Glomus* cf. *diaphanum* 589 clone 589.3 28S large subunit ribosomal RNA gene, partial sequence	*Glomus diaphanum*
DQ469120	*Rhizophagus* cf. *intraradices* HG-2010 isolate iv1 clone iv1.5 28S large subunit ribosomal RNA gene, partial sequence	*Rhizophagus intraradices*
Filcons#33	Uncultured *Rhizophagus* partial 28S RRNA gene , OTU AMF_C019Rhirr	*Rhizophagus* sp.
FM992382	*Glomus* sp. Att690-23 18S rRNA gene (partial), ITS1, 5.8S rRNA gene, ITS2 and 28S rRNA gene (partial), isolate Att690-23 (DAOM197198), clone pHS111-6	*Glomus* sp.
FR750081	*Rhizophagus irregularis* 18S rRNA gene (partial), ITS1, 5.8S rRNA gene, ITS2 and 28S rRNA gene (partial), isolate MUCL43195, clone pHS037-5	*Rhizophagus irregularis*
FR750083	*Paraglomus laccatum* 18S rRNA gene (partial), ITS1, 5.8S rRNA gene, ITS2 and 28S rRNA gene (partial), clone pHS038-1	*Paraglomus laccatum*
FR750091	*Rhizophagus* cf. *irregularis* MUCL 46240 18S rRNA gene (partial), ITS1, 5.8S rRNA gene, ITS2 and 28S rRNA gene (partial), isolate MUCL46240, clone pHS050-44	*Rhizophagus irregularis*
FR750095	*Rhizophagus* sp. MUCL 43208 18S rRNA gene (partial), ITS1, 5.8S rRNA gene, ITS2 and 28S rRNA gene (partial), isolate MUCL43208, clone pHS055-47	*Rhizophagus* sp.
FR750116	*Rhizophagus* cf. *irregularis* MUCL 43205 18S rRNA gene (partial), ITS1, 5.8S rRNA gene, ITS2 and 28S rRNA gene (partial), isolate MUCL43205, clone pHS058-6	*Rhizophagus irregularis*

Taxa ID	Identification by LSU rDNA database	Description
FR750117	*Rhizophagus* cf. *irregularis* MUCL 43205 18S rRNA gene (partial), ITS1, 5.8S rRNA gene, ITS2 and 28S rRNA gene (partial), isolate MUCL43205, clone pHS058-7	*Rhizophagus irregularis*
FR750188	*Rhizophagus irregularis* 18S rRNA gene (partial), ITS1, 5.8S rRNA gene, ITS2 and 28S rRNA gene (partial), isolate Att857-12, clone pMK100-4	*Rhizophagus irregularis*
FR750190	*Rhizophagus irregularis* 18S rRNA gene (partial), ITS1, 5.8S rRNA gene, ITS2 and 28S rRNA gene (partial), isolate Att857-12, clone pMK100-7	*Rhizophagus irregularis*
Gintra691BEG144	*Glomus _intraradices* _691_BEG144	*Glomus intraradices*
Gintx99640BEG141	*Glomus _intraradices* _691_BEG141	*Glomus intraradices*
HE794038	*Glomus* sp. PB genomic DNA containing 18S rRNA gene, ITS1, 5.8S rRNA gene, ITS2 and 28S rRNA gene, strain PB, clone pb1	*Glomus* sp.
HF548860	*Septoglomus viscosum* genomic DNA containing 18S rRNA gene, ITS1, 5.8S rRNA gene, ITS2, 28S rRNA gene, strain BEG27, isolate Att179-15, clone pDS001-32	*Septoglomus viscosum*
HF968916	*Rhizophagus irregularis* genomic DNA containing 18S rRNA gene, ITS1, 5.8S rRNA gene, ITS2 and 28S rRNA gene, strain DAOM181602, isolate spore 1, clone EI2_3_7	*Rhizophagus irregularis*
HF968919	*Rhizophagus irregularis* genomic DNA containing 18S rRNA gene, ITS1, 5.8S rRNA gene, ITS2 and 28S rRNA gene, strain DAOM181602, isolate spore 1, clone EI2_3_8	*Rhizophagus irregularis*
HF968920	*Rhizophagus irregularis* genomic DNA containing 18S rRNA gene, ITS1, 5.8S rRNA gene, ITS2 and 28S rRNA gene, strain DAOM181602, isolate spore 1, clone EI2_3_9	*Rhizophagus irregularis*
HF968926	*Rhizophagus irregularis* genomic DNA containing 18S rRNA gene, ITS1, 5.8S rRNA gene, ITS2 and 28S rRNA gene, strain DAOM181602, isolate spore 2, clone EI2_5_5	*Rhizophagus irregularis*
HF968983	*Rhizophagus irregularis* partial 28S rRNA gene, strain DAOM181602, isolate spore 3, clone EL2_7_75	*Rhizophagus irregularis*
HF968985	*Rhizophagus irregularis* partial 28S rRNA gene, strain DAOM229456, isolate spore 2, clone 03L2_7_3	*Rhizophagus irregularis*
HF968987	*Rhizophagus irregularis* partial 28S rRNA gene, strain DAOM181602, isolate spore 1, clone EL2_3_29	*Rhizophagus irregularis*
HF968991	*Rhizophagus irregularis* partial 28S rRNA gene, strain DAOM181602, isolate spore 2, clone EL2_5_24	*Rhizophagus irregularis*
HF968992	*Rhizophagus irregularis* partial 28S rRNA gene, strain DAOM181602, isolate spore 2, clone EL2_5_59	*Rhizophagus irregularis*
HG969382	*Glomus invermaium* genomic DNA containing 18S rRNA gene, ITS1, 5.8S rRNA gene, ITS2 and 28S rRNA gene, isolate Att1646, clone ECU102P12	*Rhizophagus invermaium*
HG969387	*Glomus invermaium* genomic DNA containing 18S rRNA gene, ITS1, 5.8S rRNA gene, ITS2 and 28S rRNA gene, isolate Att1646, clone ECU102P20	*Rhizophagus invermaium*
HG969388	*Glomus invermaium* genomic DNA containing 18S rRNA gene, ITS1, 5.8S rRNA gene, ITS2 and 28S rRNA gene, isolate Att1646, clone ECU102P30	*Rhizophagus invermaium*
HG969390	*Glomus invermaium* genomic DNA containing 18S rRNA gene, ITS1, 5.8S rRNA gene, ITS2 and 28S rRNA gene, isolate Att1646, clone ECU102P13	*Rhizophagus invermaium*
HM625892	*Glomus intraradices* isolate intra6 28S ribosomal RNA gene, partial sequence	*Rhizophagus intraradices*
HM625896	*Glomus intraradices* isolate intra10 28S ribosomal RNA gene, partial sequence	*Rhizophagus intraradices*
JF439109	*Glomus intraradices* isolate B22-11 18S ribosomal RNA gene, partial sequence; internal transcribed spacer 1, 5.8S ribosomal RNA gene, and internal transcribed spacer 2, complete sequence; and 28S ribosomal RNA gene, partial sequence	*Rhizophagus intraradices*
JF439138	*Glomus intraradices* isolate B22-11 18S ribosomal RNA gene, partial sequence; internal transcribed spacer 1, 5.8S ribosomal RNA gene, and internal transcribed spacer 2, complete sequence; and 28S ribosomal RNA gene, partial sequence	*Rhizophagus intraradices*
JF439161	*Glomus* sp. 9 SUN-2011 isolate 08_48_1 18S ribosomal RNA gene, partial sequence; internal transcribed spacer 1, 5.8S ribosomal RNA gene, and internal transcribed spacer 2, complete sequence; and 28S ribosomal RNA gene, partial sequence	*Glomus* sp.
JF439167	*Glomus constrictum* isolate 08_48_12 18S ribosomal RNA gene, partial sequence; internal transcribed spacer 1, 5.8S ribosomal RNA gene, and internal transcribed spacer 2, complete sequence; and 28S ribosomal RNA gene, partial sequence	*Septoglomus constrictum*
JF439169	*Glomus intraradices* isolate 08_62_4 18S ribosomal RNA gene, partial sequence; internal transcribed spacer 1, 5.8S ribosomal RNA gene, and internal transcribed spacer 2, complete sequence; and 28S ribosomal RNA gene, partial sequence	*Rhizophagus intraradices*
JF439175	UNVERIFIED: *Glomus* sp. 20 SUN-2011 isolate 08_48_10 18S ribosomal RNA gene, partial sequence; internal transcribed spacer 1, 5.8S ribosomal RNA gene, and internal transcribed spacer 2, complete sequence; and 28S ribosomal RNA gene, partial sequence	*Glomus* sp.
JF439190	*Glomus aggregatum* isolate 08_6_1 18S ribosomal RNA gene, partial sequence; internal transcribed spacer 1, 5.8S ribosomal RNA gene, and internal transcribed spacer 2, complete sequence; and 28S ribosomal RNA gene, partial sequence	*Glomus aggregatum*
JF439202	*Glomus* sp. 11 SUN-2011 isolate 07_10_1 18S ribosomal RNA gene, partial sequence; internal transcribed spacer 1, 5.8S ribosomal RNA gene, and internal transcribed spacer 2, complete sequence; and 28S ribosomal RNA gene, partial sequence	*Glomus* sp.
JN417518	*Rhizophagus irregularis* clone 2.4 18S ribosomal RNA gene, partial sequence; internal transcribed spacer 1, 5.8S ribosomal RNA gene, and internal transcribed spacer 2, complete sequence; and 28S ribosomal RNA gene, partial sequence	*Rhizophagus irregularis*

Taxa ID	Identification by LSU rDNA database	Description
JN417519	*Rhizophagus irregularis* clone 2.18 18S ribosomal RNA gene, partial sequence; internal transcribed spacer 1, 5.8S ribosomal RNA gene, and internal transcribed spacer 2, complete sequence; and 28S ribosomal RNA gene, partial sequence	*Rhizophagus irregularis*
JQ048895	*Funneliformis geosporum* strain BEG11 clone Hsp40-13b 28S ribosomal RNA (LSU) gene, partial sequence	*Funneliformis geosporum*
KC182036	*Septoglomus viscosum* clone HSp82-2 18S ribosomal RNA gene, partial sequence; internal transcribed spacer 1, 5.8S ribosomal RNA gene, and internal transcribed spacer 2, complete sequence; and 28S ribosomal RNA gene, partial sequence	*Septoglomus viscosum*
KC182037	*Septoglomus viscosum* clone HSp82-9 18S ribosomal RNA gene, partial sequence; internal transcribed spacer 1, 5.8S ribosomal RNA gene, and internal transcribed spacer 2, complete sequence; and 28S ribosomal RNA gene, partial sequence	*Septoglomus viscosum*
KF060322	*Septoglomus jasnowskae* isolate 206-5 18S ribosomal RNA gene, partial sequence; internal transcribed spacer 1, 5.8S ribosomal RNA gene, and internal transcribed spacer 2, complete sequence; and 28S ribosomal RNA gene, partial sequence	Septoglomus jasnowskae
KF836904	*Glomus* sp. 7 ZHNL-2013a isolate D1_23 18S ribosomal RNA gene, partial sequence; internal transcribed spacer 1, 5.8S ribosomal RNA gene, and internal transcribed spacer 2, complete sequence; and 28S ribosomal RNA gene, partial sequence	*Glomus* sp.
KF836920	*Glomus* sp. 11 ZHNL-2013s isolate D4_45 18S ribosomal RNA gene, partial sequence; internal transcribed spacer 1, 5.8S ribosomal RNA gene, and internal transcribed spacer 2, complete sequence; and 28S ribosomal RNA gene, partial sequence	*Glomus* sp.
KF836959	*Rhizophagus intraradices* isolate E5_11 18S ribosomal RNA gene, partial sequence; internal transcribed spacer 1, 5.8S ribosomal RNA gene, and internal transcribed spacer 2, complete sequence; and 28S ribosomal RNA gene, partial sequence	*Rhizophagus intraradices*
KF836962	*Rhizophagus intraradices* isolate E5_18 18S ribosomal RNA gene, partial sequence; internal transcribed spacer 1, 5.8S ribosomal RNA gene, and internal transcribed spacer 2, complete sequence; and 28S ribosomal RNA gene, partial sequence	*Rhizophagus intraradices*
KP191483	*Claroideoglomus drummondii* isolate 120-13 18S ribosomal RNA gene, partial sequence; internal transcribed spacer 1, 5.8S ribosomal RNA gene, and internal transcribed spacer 2, complete sequence; and 28S ribosomal RNA gene, partial sequence	*Claroideoglomus drummondii*
KP191484	*Claroideoglomus drummondii* isolate 120-21 18S ribosomal RNA gene, partial sequence; internal transcribed spacer 1, 5.8S ribosomal RNA gene, and internal transcribed spacer 2, complete sequence; and 28S ribosomal RNA gene, partial sequence	*Claroideoglomus drummondii*
MTTD1seq	*Rhizophagus _irregularis*	*Rhizophagus irregularis*

Table S2. Identification of *de novo* taxa by BLAST analysis against NCBI database. Highlighted in yellow the taxa with median abundance higher than 0. In the last column, culturable taxa are highlighted in green.

query	subject	Bs1S		Bs2S		Rs1S		Rs2S		Description
		% coverage	bp query	% coverage	bp query	% coverage	bp query	% coverage	bp query	
denovo_1	KF849683.1	94.77	708	95.32	727	95.35	709	94.82	714	Uncultured *Glomus* clone AM203 18S ribosomal RNA gene, partial sequence; internal transcribed spacer 1, 5.8S ribosomal RNA gene, and internal transcribed spacer 2, complete sequence; and 28S ribosomal RNA gene, partial sequence
denovo_10042	JQ029749.1	-	-	-	-	97.68	690	97.26	693	Uncultured *Glomus* clone 44 large subunit ribosomal RNA gene, partial sequence
denovo_10180	JQ029749.1	-	-	-	-	97.53	648	97.62	671	Uncultured *Glomus* clone 44 large subunit ribosomal RNA gene, partial sequence
denovo_1035	KC411147.1	96.47	481	93.90	656	94.23	658	94.23	676	Uncultured Glomerales clone H19_29 large subunit ribosomal RNA gene, partial sequence
denovo_10703	DQ469113.1	-	-	-	-	-	-	93.59	671	*Glomus* cf. *diaphanum* 589 clone 589.1 28S large subunit ribosomal RNA gene, partial sequence
denovo_10707	AB643635.1	-	-	-	-	-	-	96.98	695	Uncultured *Glomus* gene for 28S ribosomal RNA, partial sequence, clone: KBM6-8
denovo_10711	AB643635.1	-	-	-	-	-	-	92.69	711	Uncultured *Glomus* gene for 28S ribosomal RNA, partial sequence, clone: KBM6-8
denovo_10732	AY639299.1	-	-	-	-	-	-	95.52	715	*Rhizophagus* cf. *intraradices* HG-2010 isolate 57 clone 57.6.3 28S large subunit ribosomal RNA gene, partial sequence
denovo_10746	AB643635.1	-	-	-	-	97.92	721	97.34	714	Uncultured *Glomus* gene for 28S ribosomal RNA, partial sequence, clone: KBM6-8
denovo_10791	KF849683.1	-	-	-	-	-	-	96.32	707	Uncultured *Glomus* clone AM203 18S ribosomal RNA gene, partial sequence; internal transcribed spacer 1, 5.8S ribosomal RNA gene, and internal transcribed spacer 2, complete sequence; and 28S ribosomal RNA gene, partial sequence
denovo_10871	AB643635.1	-	-	-	-	-	-	95.46	705	Uncultured *Glomus* gene for 28S ribosomal RNA, partial sequence, clone: KBM6-8
denovo_11223	JX683749.1	-	-	-	-	96.73	733	-	-	Uncultured Glomeraceae clone Agro-18-S 28S ribosomal RNA gene, partial sequence
denovo_11235	KC411094.1	-	-	-	-	96.94	718	-	-	Uncultured Glomerales clone H17_24 large subunit ribosomal RNA gene, partial sequence
denovo_11255	JX683749.1	-	-	-	-	95.98	722	-	-	Uncultured Glomeraceae clone Agro-18-S 28S ribosomal RNA gene, partial sequence
denovo_11260	KC182037.1	-	-	-	-	95.99	723	-	-	*Septoglomus viscosum* clone HSp82-9 18S ribosomal RNA gene, partial sequence; internal transcribed spacer 1, 5.8S ribosomal RNA gene, and internal transcribed spacer 2, complete sequence; and 28S ribosomal RNA gene, partial sequence
denovo_11492	KC410882.1	-	-	-	-	-	-	93.08	679	Uncultured Glomerales clone H1_14 large subunit ribosomal RNA gene, partial sequence
denovo_11548	AB369760.1	-	-	-	-	97.02	638	98.32	477	Uncultured *Glomus* gene for 28S ribosomal RNA, partial sequence, clone: H5-2
denovo_11599	KC411135.1	-	-	-	-	-	-	88.05	611	Uncultured Glomerales clone H19_14 large subunit ribosomal RNA gene, partial sequence
denovo_11634	KC411217.1	-	-	-	-	-	-	88.19	720	Uncultured Glomerales clone B04_50 large subunit ribosomal RNA gene, partial sequence
denovo_11692	AB369760.1	-	-	-	-	98.03	608	97.12	486	Uncultured *Glomus* gene for 28S ribosomal RNA, partial sequence, clone: H5-2
denovo_11699	KF849683.1	-	-	-	-	-	-	92.16	689	Uncultured *Glomus* clone AM203 18S ribosomal RNA gene, partial sequence; internal transcribed spacer 1, 5.8S ribosomal RNA gene, and internal transcribed spacer 2, complete sequence; and 28S ribosomal RNA gene, partial sequence
denovo_11975	HG425877.1	-	-	-	-	-	-	95.42	655	Uncultured *Rhizophagus* genomic DNA containing 18S rRNA gene, ITS1, 5.8S rRNA gene, ITS2 and 28S rRNA gene, clone JerabK4
denovo_12631	HG425883.1	-	-	-	-	97.34	715	96.52	689	Uncultured *Rhizophagus* genomic DNA containing 18S rRNA gene, ITS1, 5.8S rRNA gene, ITS2 and 28S rRNA gene, clone Jasan 8
denovo_13023	HG425888.1	-	-	-	-	-	-	97.19	676	Uncultured *Rhizophagus* genomic DNA containing 18S rRNA gene, ITS1, 5.8S rRNA gene, ITS2 and 28S rRNA gene, clone JavorIX1
denovo_13068	HQ243398.1	-	-	-	-	-	-	96.92	714	Uncultured *Glomus* clone ZHwq2-606 18S ribosomal RNA gene, partial sequence; internal transcribed spacer 1, 5.8S ribosomal RNA gene, and internal transcribed spacer 2, complete sequence; and 28S ribosomal RNA gene, partial sequence
denovo_13084	HG425888.1	-	-	-	-	-	-	98.73	472	Uncultured *Rhizophagus* genomic DNA containing 18S rRNA gene, ITS1, 5.8S rRNA gene, ITS2 and 28S rRNA gene, clone JavorIX1
denovo_13096	HQ243398.1	-	-	-	-	-	-	96.63	564	Uncultured *Glomus* clone ZHwq2-606 18S ribosomal RNA gene, partial sequence; internal transcribed spacer 1, 5.8S ribosomal RNA gene, and internal transcribed spacer 2, complete sequence; and 28S ribosomal RNA gene, partial sequence
denovo_13158	HG425888.1	-	-	-	-	94.13	681	94.77	708	Uncultured *Rhizophagus* genomic DNA containing 18S rRNA gene, ITS1, 5.8S rRNA gene, ITS2 and 28S rRNA gene, clone JavorIX1
denovo_13289	AB369732.1	-	-	-	-	-	-	96.74	705	Uncultured *Glomus* gene for 28S ribosomal RNA, partial sequence, clone: R7-16
denovo_13347	AB369732.1	-	-	-	-	-	-	94.00	583	Uncultured *Glomus* gene for 28S ribosomal RNA, partial sequence, clone: R7-16
denovo_1351	KC411147.1	-	-	95.63	503	-	-	93.70	651	Uncultured Glomerales clone H19_29 large subunit ribosomal RNA gene, partial sequence
denovo_137	KF849683.1	94.78	709	95.20	687	94.78	651	92.17	702	Uncultured *Glomus* clone AM203 18S ribosomal RNA gene, partial sequence; internal transcribed spacer 1, 5.8S ribosomal RNA gene, and internal transcribed spacer 2, complete sequence; and 28S ribosomal RNA gene, partial sequence
denovo_13921	AB643635.1	-	-	-	-	97.95	730	-	-	Uncultured *Glomus* gene for 28S ribosomal RNA, partial sequence, clone: KBM6-8
denovo_13922	JQ029749.1	-	-	-	-	97.72	703	96.91	712	Uncultured *Glomus* clone 44 large subunit ribosomal RNA gene, partial sequence
denovo_13923	FR871389.1	-	-	-	-	97.01	703	-	-	Uncultured *Glomus* partial 28S rRNA gene, clone BRAS3-30
denovo_13931	AB643635.1	-	-	-	-	96.62	680	-	-	Uncultured *Glomus* gene for 28S ribosomal RNA, partial sequence, clone: KBM6-8
denovo_13937	KC411217.1	-	-	-	-	93.16	716	-	-	Uncultured Glomerales clone B04_50 large subunit ribosomal RNA gene, partial sequence
denovo_13955	HG425889.1	-	-	-	-	93.89	704	94.62	725	Uncultured *Rhizophagus* genomic DNA containing 18S rRNA gene, ITS1, 5.8S rRNA gene, ITS2 and 28S rRNA gene, clone Olse K-X19
denovo_13960	AJ854588.1	-	-	-	-	96.94	653	-	-	*Glomus* sp. MUCL 43203 28S rRNA gene, strain MUCL 43203, clone 5
denovo_13961	HG425888.1	-	-	-	-	97.71	656	-	-	Uncultured *Rhizophagus* genomic DNA containing 18S rRNA gene, ITS1, 5.8S rRNA gene, ITS2 and 28S rRNA gene, clone JavorIX1
denovo_14137	FR871363.1	-	-	-	-	92.85	713	-	-	Uncultured *Glomus* partial 28S rRNA gene, clone BROS3-12

query	subject	Bs1S		Bs2S		Rs1S		Rs2S		Description
		% coverage	bp query	% coverage	bp query	% coverage	bp query	% coverage	bp query	
denovo_14323	HG425888.1	-	-	-	-	95.20	688	95.84	721	Uncultured *Rhizophagus* genomic DNA containing 18S rRNA gene, ITS1, 5.8S rRNA gene, ITS2 and 28S rRNA gene, clone JavorIX1
denovo_1437	HE775338.1	99.01	709	-	-	-	-	-	-	Uncultured Glomeraceae 18S rRNA gene (partial), ITS1, 5.8S rRNA gene, ITS2 and 28S rRNA gene (partial), tissue library 6/2-7/2-11/2, clone library KRU, clone 37
denovo_14387	HQ243030.1	-	-	-	-	95.84	673	95.76	707	Uncultured *Glomus* clone ZHwq2-238 18S ribosomal RNA gene, partial sequence; internal transcribed spacer 1, 5.8S ribosomal RNA gene, and internal transcribed spacer 2, complete sequence; and 28S ribosomal RNA gene, partial sequence
denovo_1439	KJ564151.1	98.45	710	-	-	-	-	-	-	*Dominikia iranica* strain 187-2-7 clone 71 18S ribosomal RNA gene, partial sequence; internal transcribed spacer 1, 5.8S ribosomal RNA gene, and internal transcribed spacer 2, complete sequence; and 28S ribosomal RNA gene, partial sequence
denovo_1440	KF849683.1	95.06	709	-	-	95.04	705	94.71	681	Uncultured *Glomus* clone AM203 18S ribosomal RNA gene, partial sequence; internal transcribed spacer 1, 5.8S ribosomal RNA gene, and internal transcribed spacer 2, complete sequence; and 28S ribosomal RNA gene, partial sequence
denovo_1442	KF849683.1	94.64	690	94.17	703	93.92	691	94.77	708	Uncultured *Glomus* clone AM203 18S ribosomal RNA gene, partial sequence; internal transcribed spacer 1, 5.8S ribosomal RNA gene, and internal transcribed spacer 2, complete sequence; and 28S ribosomal RNA gene, partial sequence
denovo_1447	HG425888.1	99.15	704	98.82	680	97.59	705	98.68	681	Uncultured *Rhizophagus* genomic DNA containing 18S rRNA gene, ITS1, 5.8S rRNA gene, ITS2 and 28S rRNA gene, clone JavorIX1
denovo_1448	HQ243030.1	97.98	694	97.27	695	97.61	711	98.17	709	Uncultured *Glomus* clone ZHwq2-238 18S ribosomal RNA gene, partial sequence; internal transcribed spacer 1, 5.8S ribosomal RNA gene, and internal transcribed spacer 2, complete sequence; and 28S ribosomal RNA gene, partial sequence
denovo_1452	KF849683.1	99.01	708	-	-	99.01	710	99.29	707	Uncultured *Glomus* clone AM203 18S ribosomal RNA gene, partial sequence; internal transcribed spacer 1, 5.8S ribosomal RNA gene, and internal transcribed spacer 2, complete sequence; and 28S ribosomal RNA gene, partial sequence
denovo_1453	JX096617.1	97.48	714	-	-	97.16	739	-	-	Uncultured Glomeromycota clone 15_18 18S ribosomal RNA gene, partial sequence; internal transcribed spacer 1, 5.8S ribosomal RNA gene, and internal transcribed spacer 2, complete sequence; and 28S ribosomal RNA gene, partial sequence
denovo_1456	KF849683.1	98.54	687	-	-	99.13	690	99.56	686	Uncultured *Glomus* clone AM203 18S ribosomal RNA gene, partial sequence; internal transcribed spacer 1, 5.8S ribosomal RNA gene, and internal transcribed spacer 2, complete sequence; and 28S ribosomal RNA gene, partial sequence
denovo_1460	KP756522.1	98.73	708	-	-	97.70	651	97.17	707	*Glomus* sp. 2 SL-2017 isolate SR6 clone 1 18S ribosomal RNA gene, partial sequence; internal transcribed spacer 1, 5.8S ribosomal RNA gene, and internal transcribed spacer 2, complete sequence; and 28S ribosomal RNA gene, partial sequence
denovo_14764	JQ029749.1	-	-	-	-	-	-	96.90	709	Uncultured *Glomus* clone 44 large subunit ribosomal RNA gene, partial sequence
denovo_1482	HE858409.1	97.14	594	-	-	96.53	721	-	-	Uncultured *Glomus* partial 28S rRNA gene, clone FW5-11
denovo_14862	HE858378.1	-	-	-	-	-	-	94.52	711	Uncultured *Glomus* partial 28S rRNA gene, clone WW2-5
denovo_1489	KF849683.1	98.73	707	-	-	98.59	708	99.11	677	Uncultured *Glomus* clone AM203 18S ribosomal RNA gene, partial sequence; internal transcribed spacer 1, 5.8S ribosomal RNA gene, and internal transcribed spacer 2, complete sequence; and 28S ribosomal RNA gene, partial sequence
denovo_1493	HE775295.1	91.73	713	-	-	90.96	708	-	-	Uncultured *Rhizophagus* 18S rRNA gene (partial), ITS1, 5.8S rRNA gene, ITS2 and 28S rRNA gene (partial), tissue library 32e, clone library KRU, clone 17
denovo_1503	KC410882.1	95.95	568	-	-	-	-	95.56	586	Uncultured Glomerales clone H1_14 large subunit ribosomal RNA gene, partial sequence
denovo_1511	HE858409.1	96.94	719	-	-	96.84	664	-	-	Uncultured *Glomus* partial 28S rRNA gene, clone FW5-11
denovo_1517	KF849683.1	94.45	613	94.64	671	93.92	674	96.16	443	Uncultured *Glomus* clone AM203 18S ribosomal RNA gene, partial sequence; internal transcribed spacer 1, 5.8S ribosomal RNA gene, and internal transcribed spacer 2, complete sequence; and 28S ribosomal RNA gene, partial sequence
denovo_1621	FR871386.1	88.74	684	88.28	725	96.68	723	87.63	663	Uncultured *Glomus* partial 28S rRNA gene, clone BROS3-13
denovo_1627	KF849683.1	98.62	651	-	-	98.40	688	99.10	666	Uncultured *Glomus* clone AM203 18S ribosomal RNA gene, partial sequence; internal transcribed spacer 1, 5.8S ribosomal RNA gene, and internal transcribed spacer 2, complete sequence; and 28S ribosomal RNA gene, partial sequence
denovo_1630	FR871325.1	-	-	-	-	97.94	630	-	-	Uncultured *Glomus* partial 28S rRNA gene, clone BRAR3-7
denovo_1633	HG425888.1	99.40	666	-	-	98.14	699	96.47	737	Uncultured *Rhizophagus* genomic DNA containing 18S rRNA gene, ITS1, 5.8S rRNA gene, ITS2 and 28S rRNA gene, clone JavorIX1
denovo_1647	HG425890.1	98.59	639	-	-	97.90	665	98.53	681	Uncultured *Rhizophagus* genomic DNA containing 18S rRNA gene, ITS1, 5.8S rRNA gene, ITS2 and 28S rRNA gene, clone HlohK18
denovo_1726	KF849683.1	94.15	650	94.11	713	-	-	95.45	505	Uncultured *Glomus* clone AM203 18S ribosomal RNA gene, partial sequence; internal transcribed spacer 1, 5.8S ribosomal RNA gene, and internal transcribed spacer 2, complete sequence; and 28S ribosomal RNA gene, partial sequence
denovo_1827	KF849683.1	98.02	656	-	-	98.99	691	99.42	691	Uncultured *Glomus* clone AM203 18S ribosomal RNA gene, partial sequence; internal transcribed spacer 1, 5.8S ribosomal RNA gene, and internal transcribed spacer 2, complete sequence; and 28S ribosomal RNA gene, partial sequence
denovo_1835	KF849683.1	94.82	676	-	-	94.85	680	94.29	666	Uncultured *Glomus* clone AM203 18S ribosomal RNA gene, partial sequence; internal transcribed spacer 1, 5.8S ribosomal RNA gene, and internal transcribed spacer 2, complete sequence; and 28S ribosomal RNA gene, partial sequence
denovo_1841	KF849683.1	94.60	556	-	-	93.89	671	95.56	585	Uncultured *Glomus* clone AM203 18S ribosomal RNA gene, partial sequence; internal transcribed spacer 1, 5.8S ribosomal RNA gene, and internal transcribed spacer 2, complete sequence; and 28S ribosomal RNA gene, partial sequence
denovo_1882	HG425890.1	98.65	592	-	-	99.36	624	95.71	699	Uncultured *Rhizophagus* genomic DNA containing 18S rRNA gene, ITS1, 5.8S rRNA gene, ITS2 and 28S rRNA gene, clone HlohK18
denovo_1903	FR750106.1	-	-	89.93	725	90.20	714	89.52	725	*Rhizophagus* cf. *irregularis* MUCL 43205 18S rRNA gene (partial), ITS1, 5.8S rRNA gene, ITS2 and 28S rRNA gene (partial), isolate MUCL43205, clone pHS058-1
denovo_1988	DQ469113.1	88.95	697	90.59	712	90.31	712	89.12	717	*Glomus* cf. *diaphanum* 589 clone 589.1 28S large subunit ribosomal RNA gene, partial sequence

query	subject	Bs1S		Bs2S		Rs1S		Rs2S		Description
		% coverage	bp query	% coverage	bp query	% coverage	bp query	% coverage	bp query	
denovo_1994	JQ029748.1	98.97	583	-	-	97.66	641	97.45	706	Uncultured *Glomus* clone 18 large subunit ribosomal RNA gene, partial sequence
denovo_2	KF849683.1	94.09	711	94.79	710	95.34	708	95.60	705	Uncultured *Glomus* clone AM203 18S ribosomal RNA gene, partial sequence; internal transcribed spacer 1, 5.8S ribosomal RNA gene, and internal transcribed spacer 2, complete sequence; and 28S ribosomal RNA gene, partial sequence
denovo_2019	KF849683.1	-	-	-	-	-	-	93.08	708	Uncultured *Glomus* clone AM203 18S ribosomal RNA gene, partial sequence; internal transcribed spacer 1, 5.8S ribosomal RNA gene, and internal transcribed spacer 2, complete sequence; and 28S ribosomal RNA gene, partial sequence
denovo_2031	HF970215.1	97.83	506	-	-	97.89	710	-	-	Uncultured *Rhizophagus* genomic DNA containing 18S rRNA gene, ITS1, 5.8S rRNA gene, ITS2 and 28S rRNA gene, clone CS029-05
denovo_212	KF849683.1	93.54	712	-	-	95.08	712	94.80	711	Uncultured *Glomus* clone AM203 18S ribosomal RNA gene, partial sequence; internal transcribed spacer 1, 5.8S ribosomal RNA gene, and internal transcribed spacer 2, complete sequence; and 28S ribosomal RNA gene, partial sequence
denovo_2141	HG425888.1	99.35	612	-	-	98.40	626	99.40	497	Uncultured *Rhizophagus* genomic DNA containing 18S rRNA gene, ITS1, 5.8S rRNA gene, ITS2 and 28S rRNA gene, clone JavorIX1
denovo_2348	KF849683.1	-	-	-	-	99.22	640	99.28	691	Uncultured *Glomus* clone AM203 18S ribosomal RNA gene, partial sequence; internal transcribed spacer 1, 5.8S ribosomal RNA gene, and internal transcribed spacer 2, complete sequence; and 28S ribosomal RNA gene, partial sequence
denovo_2351	KC411452.1	87.59	709	87.12	714	87.80	713	87.18	733	Uncultured Glomerales clone B18_36 large subunit ribosomal RNA gene, partial sequence
denovo_2381	HG425889.1	90.09	706	-	-	88.90	721	-	-	Uncultured *Rhizophagus* genomic DNA containing 18S rRNA gene, ITS1, 5.8S rRNA gene, ITS2 and 28S rRNA gene, clone Olse K-X19
denovo_2396	KF849683.1	98.39	685	-	-	98.71	621	99.18	611	Uncultured *Glomus* clone AM203 18S ribosomal RNA gene, partial sequence; internal transcribed spacer 1, 5.8S ribosomal RNA gene, and internal transcribed spacer 2, complete sequence; and 28S ribosomal RNA gene, partial sequence
denovo_2489	KC410882.1	87.86	667	-	-	88.25	732	87.20	719	Uncultured Glomerales clone H1_14 large subunit ribosomal RNA gene, partial sequence
denovo_2505	KF849683.1	94.55	440	94.77	708	94.40	714	94.75	609	Uncultured *Glomus* clone AM203 18S ribosomal RNA gene, partial sequence; internal transcribed spacer 1, 5.8S ribosomal RNA gene, and internal transcribed spacer 2, complete sequence; and 28S ribosomal RNA gene, partial sequence
denovo_2674	KC411100.1	-	-	95.48	708	94.94	711	-	-	Uncultured Glomerales clone H17_30 large subunit ribosomal RNA gene, partial sequence
denovo_27	KF849683.1	95.14	679	95.14	679	95.62	708	95.01	681	Uncultured *Glomus* clone AM203 18S ribosomal RNA gene, partial sequence; internal transcribed spacer 1, 5.8S ribosomal RNA gene, and internal transcribed spacer 2, complete sequence; and 28S ribosomal RNA gene, partial sequence
denovo_2709	HQ242943.1	-	-	95.28	699	-	-	-	-	Uncultured *Glomus* clone ZHwq2-151 18S ribosomal RNA gene, partial sequence; internal transcribed spacer 1, 5.8S ribosomal RNA gene, and internal transcribed spacer 2, complete sequence; and 28S ribosomal RNA gene, partial sequence
denovo_2749	FJ461841.1	97.44	507	97.08	719	-	-	-	-	*Glomus macrocarpum* isolate MD124 25S ribosomal RNA gene, partial sequence
denovo_2799	HQ243164.1	-	-	95.02	643	-	-	-	-	Uncultured *Glomus* clone ZHwq2-372 18S ribosomal RNA gene, partial sequence; internal transcribed spacer 1, 5.8S ribosomal RNA gene, and internal transcribed spacer 2, complete sequence; and 28S ribosomal RNA gene, partial sequence
denovo_2860	HQ242981.1	-	-	94.08	709	-	-	-	-	Uncultured *Glomus* clone ZHwq2-189 18S ribosomal RNA gene, partial sequence; internal transcribed spacer 1, 5.8S ribosomal RNA gene, and internal transcribed spacer 2, complete sequence; and 28S ribosomal RNA gene, partial sequence
denovo_2869	KC411147.1	-	-	95.46	507	94.13	647	95.52	446	Uncultured Glomerales clone H19_29 large subunit ribosomal RNA gene, partial sequence
denovo_2958	KF849683.1	-	-	93.79	709	-	-	93.22	708	Uncultured *Glomus* clone AM203 18S ribosomal RNA gene, partial sequence; internal transcribed spacer 1, 5.8S ribosomal RNA gene, and internal transcribed spacer 2, complete sequence; and 28S ribosomal RNA gene, partial sequence
denovo_3107	KC410882.1	-	-	94.63	652	-	-	-	-	Uncultured Glomerales clone H1_14 large subunit ribosomal RNA gene, partial sequence
denovo_3132	KC411147.1	-	-	95.09	550	-	-	-	-	Uncultured Glomerales clone H19_29 large subunit ribosomal RNA gene, partial sequence
denovo_3167	HF970216.1	98.55	622	98.15	593	-	-	-	-	Uncultured *Archaeospora* genomic DNA containing 18S rRNA gene, ITS1, 5.8S rRNA gene, ITS2 and 28S rRNA gene, clone CS030-02
denovo_3223	HF970216.1	98.85	607	99.14	467	-	-	-	-	Uncultured *Archaeospora* genomic DNA containing 18S rRNA gene, ITS1, 5.8S rRNA gene, ITS2 and 28S rRNA gene, clone CS030-02
denovo_3232	HQ243034.1	-	-	96.18	497	-	-	-	-	Uncultured *Glomus* clone ZHwq2-242 18S ribosomal RNA gene, partial sequence; internal transcribed spacer 1, 5.8S ribosomal RNA gene, and internal transcribed spacer 2, complete sequence; and 28S ribosomal RNA gene, partial sequence
denovo_344	KF849683.1	94.41	680	95.61	706	95.22	711	96.48	710	Uncultured *Glomus* clone AM203 18S ribosomal RNA gene, partial sequence; internal transcribed spacer 1, 5.8S ribosomal RNA gene, and internal transcribed spacer 2, complete sequence; and 28S ribosomal RNA gene, partial sequence
denovo_358	HF970216.1	98.45	643	98.76	643	98.14	644	-	-	Uncultured *Archaeospora* genomic DNA containing 18S rRNA gene, ITS1, 5.8S rRNA gene, ITS2 and 28S rRNA gene, clone CS030-02
denovo_381	HE858395.1	98.16	708	97.14	735	98.87	709	98.73	708	Uncultured *Glomus* partial 28S rRNA gene, clone FW4-2
denovo_4117	HE775313.1	96.19	682	-	-	-	-	-	-	Uncultured *Rhizophagus* 18S rRNA gene (partial), ITS1, 5.8S rRNA gene, ITS2 and 28S rRNA gene (partial), tissue library 2/1R-2/1-4/1, clone library KRU, clone 19
denovo_4119	FJ461841.1	98.11	689	96.81	721	-	-	-	-	*Glomus macrocarpum* isolate MD124 25S ribosomal RNA gene, partial sequence
denovo_4120	FR871386.1	95.09	509	-	-	-	-	-	-	Uncultured *Glomus* partial 28S rRNA gene, clone BROS3-13
denovo_4121	HE858375.1	98.24	510	-	-	-	-	98.28	466	Uncultured *Glomus* partial 28S rRNA gene, clone WW1-6
denovo_4124	HG425888.1	96.76	709	-	-	-	-	97.72	703	Uncultured *Rhizophagus* genomic DNA containing 18S rRNA gene, ITS1, 5.8S rRNA gene, ITS2 and 28S rRNA gene, clone JavorIX1
denovo_4125	HG969387.1	96.96	691	-	-	-	-	-	-	*Glomus invermaium* genomic DNA containing 18S rRNA gene, ITS1, 5.8S rRNA gene, ITS2 and 28S rRNA gene, isolate Att1646, clone ECU102P20
denovo_4138	KC411147.1	94.13	699	94.71	699	-	-	94.64	690	Uncultured Glomerales clone H19_29 large subunit ribosomal RNA gene, partial sequence

query	subject	Bs1S		Bs2S		Rs1S		Rs2S		Description
		% coverage	bp query	% coverage	bp query	% coverage	bp query	% coverage	bp query	
denovo_4139	KC411147.1	94.55	624	94.23	624	-	-	93.25	726	Uncultured Glomerales clone H19_29 large subunit ribosomal RNA gene, partial sequence
denovo_4147	FR750106.1	88.73	683	-	-	89.48	713	-	-	*Rhizophagus* cf. *irregularis* MUCL 43205 18S rRNA gene (partial), ITS1, 5.8S rRNA gene, ITS2 and 28S rRNA gene (partial), isolate MUCL43205, clone pHS058-1
denovo_4157	AJ854641.1	92.22	450	-	-	-	-	-	-	*Glomus* sp. MUCL 41835 28S rRNA gene, strain MUCL 41835, clone 2
denovo_4161	KC410882.1	86.91	718	-	-	-	-	-	-	Uncultured Glomerales clone H1_14 large subunit ribosomal RNA gene, partial sequence
denovo_4162	AB369731.1	95.60	681	93.67	663	-	-	94.33	706	Uncultured *Glomus* gene for 28S ribosomal RNA, partial sequence, clone: R8-52-2
denovo_4178	HE858409.1	96.53	634	-	-	-	-	-	-	Uncultured *Glomus* partial 28S rRNA gene, clone FW5-11
denovo_4184	FR871386.1	90.73	712	90.31	702	90.48	714	90.63	555	Uncultured *Glomus* partial 28S rRNA gene, clone BROS3-13
denovo_4199	HF970216.1	98.56	623	98.55	622	-	-	-	-	Uncultured *Archaeospora* genomic DNA containing 18S rRNA gene, ITS1, 5.8S rRNA gene, ITS2 and 28S rRNA gene, clone CS030-02
denovo_4210	HF970216.1	98.39	621	98.55	622	-	-	-	-	Uncultured *Archaeospora* genomic DNA containing 18S rRNA gene, ITS1, 5.8S rRNA gene, ITS2 and 28S rRNA gene, clone CS030-02
denovo_4228	HF970216.1	90.34	652	88.76	623	-	-	-	-	Uncultured *Archaeospora* genomic DNA containing 18S rRNA gene, ITS1, 5.8S rRNA gene, ITS2 and 28S rRNA gene, clone CS030-02
denovo_4298	AY639360.1	95.60	659	99.19	620	-	-	99.54	653	Uncultured Glomeromycota clone 6.11 28S large subunit ribosomal RNA gene, partial sequence
denovo_4418	AB369735.1	95.47	706	94.19	706	-	-	95.33	706	Uncultured *Glomus* gene for 28S ribosomal RNA, partial sequence, clone: A11-58
denovo_4474	KC411147.1	95.94	517	96.27	509	-	-	94.34	548	Uncultured Glomerales clone H19_29 large subunit ribosomal RNA gene, partial sequence
denovo_4480	AB643635.1	96.11	694	96.47	708	97.49	717	97.31	707	Uncultured *Glomus* gene for 28S ribosomal RNA, partial sequence, clone: KBM6-8
denovo_4499	KM208495.1	95.25	463	-	-	-	-	94.43	574	Uncultured Glomeromycota clone SLT2_34 18S ribosomal RNA gene, partial sequence; internal transcribed spacer 1, 5.8S ribosomal RNA gene, and internal transcribed spacer 2, complete sequence; and 28S ribosomal RNA gene, partial sequence
denovo_4639	AJ854628.1	88.94	714	90.07	735	89.96	707	89.55	670	*Glomus* sp. MUCL 43207 28S rRNA gene, strain MUCL 43207, clone 6
denovo_4642	KF849683.1	93.80	581	-	-	93.40	697	93.99	582	Uncultured *Glomus* clone AM203 18S ribosomal RNA gene, partial sequence; internal transcribed spacer 1, 5.8S ribosomal RNA gene, and internal transcribed spacer 2, complete sequence; and 28S ribosomal RNA gene, partial sequence
denovo_4676	HF970216.1	-	-	97.60	624	-	-	-	-	Uncultured *Archaeospora* genomic DNA containing 18S rRNA gene, ITS1, 5.8S rRNA gene, ITS2 and 28S rRNA gene, clone CS030-02
denovo_479	KC411147.1	94.22	709	94.09	710	94.35	708	-	-	Uncultured Glomerales clone H19_29 large subunit ribosomal RNA gene, partial sequence
denovo_4823	AB643635.1	-	-	96.50	714	97.08	685	97.46	668	Uncultured *Glomus* gene for 28S ribosomal RNA, partial sequence, clone: KBM6-8
denovo_4847	KM208490.1	97.28	441	-	-	-	-	95.18	706	Uncultured Glomeromycota clone SHN2_137 18S ribosomal RNA gene, partial sequence; internal transcribed spacer 1, 5.8S ribosomal RNA gene, and internal transcribed spacer 2, complete sequence; and 28S ribosomal RNA gene, partial sequence
denovo_49	KF849683.1	93.20	706	94.93	710	-	-	94.92	708	Uncultured *Glomus* clone AM203 18S ribosomal RNA gene, partial sequence; internal transcribed spacer 1, 5.8S ribosomal RNA gene, and internal transcribed spacer 2, complete sequence; and 28S ribosomal RNA gene, partial sequence
denovo_493	KC411208.1	98.73	550	96.56	669	-	-	-	-	Uncultured Glomerales clone B04_41 large subunit ribosomal RNA gene, partial sequence
denovo_4932	HF970216.1	97.74	530	97.77	628	-	-	-	-	Uncultured *Archaeospora* genomic DNA containing 18S rRNA gene, ITS1, 5.8S rRNA gene, ITS2 and 28S rRNA gene, clone CS030-02
denovo_4941	JN937295.1	-	-	96.47	594	-	-	-	-	Glomeromycota sp. OTU27 DJMC-2012 isolate spore 2_6_1 28S ribosomal RNA gene, partial sequence
denovo_4961	HF970216.1	97.95	488	98.20	445	-	-	-	-	Uncultured *Archaeospora* genomic DNA containing 18S rRNA gene, ITS1, 5.8S rRNA gene, ITS2 and 28S rRNA gene, clone CS030-02
denovo_4972	AB369921.1	-	-	97.06	713	98.59	711	-	-	Uncultured *Glomus* gene for 28S ribosomal RNA, partial sequence, clone: N11-86
denovo_498	KF849683.1	-	-	92.53	709	-	-	91.34	716	Uncultured *Glomus* clone AM203 18S ribosomal RNA gene, partial sequence; internal transcribed spacer 1, 5.8S ribosomal RNA gene, and internal transcribed spacer 2, complete sequence; and 28S ribosomal RNA gene, partial sequence
denovo_500	KC411147.1	93.94	709	93.67	695	93.80	710	92.96	724	Uncultured Glomerales clone H19_29 large subunit ribosomal RNA gene, partial sequence
denovo_5047	HF970216.1	-	-	89.57	633	-	-	-	-	Uncultured *Archaeospora* genomic DNA containing 18S rRNA gene, ITS1, 5.8S rRNA gene, ITS2 and 28S rRNA gene, clone CS030-02
denovo_5054	KC411028.1	-	-	91.15	712	-	-	-	-	Uncultured Glomerales clone H11_41 large subunit ribosomal RNA gene, partial sequence
denovo_506	FR871374.1	96.29	673	96.93	684	-	-	98.07	622	Uncultured *Glomus* partial 28S rRNA gene, clone BROS1-19
denovo_507	FR750080.1	-	-	96.21	712	95.93	712	95.44	680	*Rhizophagus irregularis* 18S rRNA gene (partial), ITS1, 5.8S rRNA gene, ITS2 and 28S rRNA gene (partial), isolate MUCL43195, clone pHS037-4
denovo_508	KC410882.1	-	-	94.14	682	93.51	709	94.39	677	Uncultured Glomerales clone H1_14 large subunit ribosomal RNA gene, partial sequence
denovo_5122	JQ029749.1	-	-	97.76	715	97.90	715	97.47	712	Uncultured *Glomus* clone 44 large subunit ribosomal RNA gene, partial sequence
denovo_525	KC411147.1	94.07	708	94.22	692	94.08	709	-	-	Uncultured Glomerales clone H19_29 large subunit ribosomal RNA gene, partial sequence
denovo_5259	HF970216.1	-	-	89.63	646	-	-	-	-	Uncultured *Archaeospora* genomic DNA containing 18S rRNA gene, ITS1, 5.8S rRNA gene, ITS2 and 28S rRNA gene, clone CS030-02
denovo_5291	HF970216.1	-	-	97.69	433	-	-	-	-	Uncultured *Archaeospora* genomic DNA containing 18S rRNA gene, ITS1, 5.8S rRNA gene, ITS2 and 28S rRNA gene, clone CS030-02
denovo_531	KC410885.1	-	-	95.46	705	-	-	94.34	707	Uncultured Glomerales clone H1_19 large subunit ribosomal RNA gene, partial sequence
denovo_5374	FR871386.1	-	-	95.58	385	96.08	383	96.11	386	Uncultured *Glomus* partial 28S rRNA gene, clone BROS3-13
denovo_540	KF849683.1	-	-	95.07	588	-	-	92.45	715	Uncultured *Glomus* clone AM203 18S ribosomal RNA gene, partial sequence; internal transcribed spacer 1, 5.8S ribosomal RNA gene, and internal transcribed spacer 2, complete sequence; and 28S ribosomal RNA gene, partial sequence
denovo_552	KC411147.1	93.95	711	93.96	679	93.86	700	93.79	676	Uncultured Glomerales clone H19_29 large subunit ribosomal RNA gene, partial sequence
denovo_553	AJ854628.1	95.17	662	96.33	708	95.82	669	96.03	706	*Glomus* sp. MUCL 43207 28S rRNA gene, strain MUCL 43207, clone 6

query	subject	Bs1S		Bs2S		Rs1S		Rs2S		Description
		% coverage	bp query	% coverage	bp query	% coverage	bp query	% coverage	bp query	
denovo_558	AJ854615.1	-	-	92.66	708	-	-	91.96	684	*Glomus* sp. MUCL 43206 28S rRNA gene, strain MUCL 43206, clone 3
denovo_5599	HF970216.1	-	-	91.36	648	-	-	-	-	Uncultured *Archaeospora* genomic DNA containing 18S rRNA gene, ITS1, 5.8S rRNA gene, ITS2 and 28S rRNA gene, clone CS030-02
denovo_561	HF970216.1	98.71	621	99.03	620	-	-	-	-	Uncultured *Archaeospora* genomic DNA containing 18S rRNA gene, ITS1, 5.8S rRNA gene, ITS2 and 28S rRNA gene, clone CS030-02
denovo_570	HM570004.1	-	-	96.55	725	96.75	708	96.60	705	Uncultured *Glomus* clone ZHwq1-2 18S ribosomal RNA gene, partial sequence; internal transcribed spacer 1, 5.8S ribosomal RNA gene, and internal transcribed spacer 2, complete sequence; and 28S ribosomal RNA gene, partial sequence
denovo_5742	KC410946.1	-	-	89.99	679	-	-	90.69	730	Uncultured Glomerales clone H3_24 large subunit ribosomal RNA gene, partial sequence
denovo_581	HF970216.1	98.58	635	98.40	626	98.23	622	-	-	Uncultured *Archaeospora* genomic DNA containing 18S rRNA gene, ITS1, 5.8S rRNA gene, ITS2 and 28S rRNA gene, clone CS030-02
denovo_593	KC411147.1	-	-	94.29	683	-	-	-	-	Uncultured Glomerales clone H19_29 large subunit ribosomal RNA gene, partial sequence
denovo_606	KF849683.1	-	-	94.38	694	-	-	94.09	711	Uncultured *Glomus* clone AM203 18S ribosomal RNA gene, partial sequence; internal transcribed spacer 1, 5.8S ribosomal RNA gene, and internal transcribed spacer 2, complete sequence; and 28S ribosomal RNA gene, partial sequence
denovo_6076	JF439109.1	96.71	639	-	-	-	-	-	-	*Glomus intraradices* isolate B22-11 18S ribosomal RNA gene, partial sequence; internal transcribed spacer 1, 5.8S ribosomal RNA gene, and internal transcribed spacer 2, complete sequence; and 28S ribosomal RNA gene, partial sequence
denovo_6080	KF060321.1	96.41	696	-	-	96.96	560	-	-	*Septoglomus jasnowskae* isolate 206-4 18S ribosomal RNA gene, partial sequence; internal transcribed spacer 1, 5.8S ribosomal RNA gene, and internal transcribed spacer 2, complete sequence; and 28S ribosomal RNA gene, partial sequence
denovo_6085	AB369760.1	98.73	707	-	-	97.74	707	98.31	710	Uncultured *Glomus* gene for 28S ribosomal RNA, partial sequence, clone: H5-2
denovo_6151	JQ029749.1	97.88	708	-	-	97.77	717	97.66	727	Uncultured *Glomus* clone 44 large subunit ribosomal RNA gene, partial sequence
denovo_6158	KC410882.1	-	-	-	-	-	-	95.76	707	Uncultured Glomerales clone H1_14 large subunit ribosomal RNA gene, partial sequence
denovo_622	KC411147.1	94.07	708	93.37	709	-	-	-	-	Uncultured Glomerales clone H19_29 large subunit ribosomal RNA gene, partial sequence
denovo_627	HF970216.1	98.42	634	98.34	604	-	-	98.55	619	Uncultured *Archaeospora* genomic DNA containing 18S rRNA gene, ITS1, 5.8S rRNA gene, ITS2 and 28S rRNA gene, clone CS030-02
denovo_628	AJ854608.1	-	-	96.77	711	96.75	647	95.49	709	*Glomus* sp. MUCL 43205 28S rRNA gene, strain MUCL 43205, clone 8
denovo_634	AJ854602.1	93.93	708	93.97	696	92.95	709	92.94	708	*Glomus* sp. MUCL 43205 28S rRNA gene, strain MUCL 43205, clone 2
denovo_6490	AJ854611.1	-	-	89.96	707	89.36	714	93.93	708	*Glomus* sp. MUCL 43205 28S rRNA gene, strain MUCL 43205, clone 11
denovo_6491	FR871386.1	-	-	96.08	383	96.35	383	96.35	384	Uncultured *Glomus* partial 28S rRNA gene, clone BROS3-13
denovo_6494	JN937243.1	-	-	91.29	712	-	-	-	-	Glomeromycota sp. OTU3 DJMC-2012 isolate spore 1_9_2 28S ribosomal RNA gene, partial sequence
denovo_6500	JN937243.1	-	-	96.76	678	-	-	-	-	Glomeromycota sp. OTU3 DJMC-2012 isolate spore 1_9_2 28S ribosomal RNA gene, partial sequence
denovo_6505	FR750081.1	-	-	88.78	713	-	-	-	-	*Rhizophagus irregularis* 18S rRNA gene (partial), ITS1, 5.8S rRNA gene, ITS2 and 28S rRNA gene (partial), isolate MUCL43195, clone pHS037-5
denovo_6508	FR750081.1	-	-	88.13	716	89.22	714	-	-	*Rhizophagus irregularis* 18S rRNA gene (partial), ITS1, 5.8S rRNA gene, ITS2 and 28S rRNA gene (partial), isolate MUCL43195, clone pHS037-5
denovo_6512	JN937243.1	-	-	97.18	710	97.13	663	96.14	700	Glomeromycota sp. OTU3 DJMC-2012 isolate spore 1_9_2 28S ribosomal RNA gene, partial sequence
denovo_6519	AJ854602.1	-	-	96.73	734	-	-	-	-	*Glomus* sp. MUCL 43205 28S rRNA gene, strain MUCL 43205, clone 2
denovo_6539	AJ854608.1	-	-	95.05	707	-	-	94.48	707	*Glomus* sp. MUCL 43205 28S rRNA gene, strain MUCL 43205, clone 8
denovo_6559	AJ854582.1	-	-	96.18	655	-	-	-	-	*Glomus intraradices* 28S rRNA gene, strain MUCL 43194, clone 9
denovo_660	FR750081.1	-	-	-	-	-	-	96.43	729	*Rhizophagus irregularis* 18S rRNA gene (partial), ITS1, 5.8S rRNA gene, ITS2 and 28S rRNA gene (partial), isolate MUCL43195, clone pHS037-5
denovo_677	HF970216.1	97.22	647	99.19	618	-	-	98.61	646	Uncultured *Archaeospora* genomic DNA containing 18S rRNA gene, ITS1, 5.8S rRNA gene, ITS2 and 28S rRNA gene, clone CS030-02
denovo_6794	AJ854588.1	-	-	96.58	585	-	-	96.81	659	*Glomus* sp. MUCL 43203 28S rRNA gene, strain MUCL 43203, clone 5
denovo_681	HF970216.1	97.84	648	98.27	637	-	-	-	-	Uncultured *Archaeospora* genomic DNA containing 18S rRNA gene, ITS1, 5.8S rRNA gene, ITS2 and 28S rRNA gene, clone CS030-02
denovo_6815	FR750106.1	-	-	94.77	707	94.90	706	94.31	703	*Rhizophagus* cf. *irregularis* MUCL 43205 18S rRNA gene (partial), ITS1, 5.8S rRNA gene, ITS2 and 28S rRNA gene (partial), isolate MUCL43205, clone pHS058-1
denovo_6828	HE775307.1	98.34	661	-	-	-	-	-	-	Uncultured *Rhizophagus* 18S rRNA gene (partial), ITS1, 5.8S rRNA gene, ITS2 and 28S rRNA gene (partial), tissue library 2/1R-2/1-4/1, clone library KRU, clone 13
denovo_6832	HE775307.1	98.19	661	-	-	-	-	-	-	Uncultured *Rhizophagus* 18S rRNA gene (partial), ITS1, 5.8S rRNA gene, ITS2 and 28S rRNA gene (partial), tissue library 2/1R-2/1-4/1, clone library KRU, clone 13
denovo_6833	FJ461841.1	97.20	715	-	-	-	-	-	-	*Glomus macrocarpum* isolate MD124 25S ribosomal RNA gene, partial sequence
denovo_6834	HE775307.1	98.34	662	-	-	-	-	-	-	Uncultured *Rhizophagus* 18S rRNA gene (partial), ITS1, 5.8S rRNA gene, ITS2 and 28S rRNA gene (partial), tissue library 2/1R-2/1-4/1, clone library KRU, clone 13
denovo_6835	HE775307.1	97.00	666	-	-	-	-	-	-	Uncultured *Rhizophagus* 18S rRNA gene (partial), ITS1, 5.8S rRNA gene, ITS2 and 28S rRNA gene (partial), tissue library 2/1R-2/1-4/1, clone library KRU, clone 13
denovo_684	KM208485.1	-	-	94.07	708	-	-	-	-	Uncultured Glomeromycota clone SLT3_39 18S ribosomal RNA gene, partial sequence; internal transcribed spacer 1, 5.8S ribosomal RNA gene, and internal transcribed spacer 2, complete sequence; and 28S ribosomal RNA gene, partial sequence

query	subject	Bs1S		Bs2S		Rs1S		Rs2S		Description
		% coverage	bp query	% coverage	bp query	% coverage	bp query	% coverage	bp query	
denovo_6851	HE775307.1	98.58	635	-	-	-	-	-	-	Uncultured *Rhizophagus* 18S rRNA gene (partial), ITS1, 5.8S rRNA gene, ITS2 and 28S rRNA gene (partial), tissue library 2/1R-2/1-4/1, clone library KRU, clone 13
denovo_6899	HE775307.1	98.13	643	-	-	-	-	-	-	Uncultured *Rhizophagus* 18S rRNA gene (partial), ITS1, 5.8S rRNA gene, ITS2 and 28S rRNA gene (partial), tissue library 2/1R-2/1-4/1, clone library KRU, clone 13
denovo_6955	HE775307.1	96.90	452	-	-	-	-	-	-	Uncultured *Rhizophagus* 18S rRNA gene (partial), ITS1, 5.8S rRNA gene, ITS2 and 28S rRNA gene (partial), tissue library 2/1R-2/1-4/1, clone library KRU, clone 13
denovo_711	HE858377.1	96.05	481	97.41	463	-	-	-	-	Uncultured *Glomus* partial 28S rRNA gene, clone WW2-2
denovo_7970	AJ854588.1	-	-	96.79	717	-	-	-	-	*Glomus* sp. MUCL 43203 28S rRNA gene, strain MUCL 43203, clone 5
denovo_7998	AJ854611.1	-	-	95.98	722	-	-	-	-	*Glomus* sp. MUCL 43205 28S rRNA gene, strain MUCL 43205, clone 11
denovo_8007	KP756522.1	-	-	96.15	650	96.97	694	96.61	648	*Glomus* sp. 2 SL-2017 isolate SR6 clone 1 18S ribosomal RNA gene, partial sequence; internal transcribed spacer 1, 5.8S ribosomal RNA gene, and internal transcribed spacer 2, complete sequence; and 28S ribosomal RNA gene, partial sequence
denovo_8017	FR871389.1	-	-	96.93	717	-	-	-	-	Uncultured *Glomus* partial 28S rRNA gene, clone BRAS3-30
denovo_804	KC411147.1	-	-	93.68	712	92.54	710	-	-	Uncultured Glomerales clone H19_29 large subunit ribosomal RNA gene, partial sequence
denovo_8143	JQ029749.1	-	-	91.83	710	92.25	710	91.70	711	Uncultured *Glomus* clone 44 large subunit ribosomal RNA gene, partial sequence
denovo_8251	KP756522.1	-	-	94.69	565	96.74	582	-	-	*Glomus* sp. 2 SL-2017 isolate SR6 clone 1 18S ribosomal RNA gene, partial sequence; internal transcribed spacer 1, 5.8S ribosomal RNA gene, and internal transcribed spacer 2, complete sequence; and 28S ribosomal RNA gene, partial sequence
denovo_843	JX096582.1	93.83	713	95.71	700	-	-	-	-	Uncultured Glomeromycota clone 4_16 18S ribosomal RNA gene, partial sequence; internal transcribed spacer 1, 5.8S ribosomal RNA gene, and internal transcribed spacer 2, complete sequence; and 28S ribosomal RNA gene, partial sequence
denovo_8534	AB643635.1	-	-	96.34	410	88.33	720	93.00	729	Uncultured *Glomus* gene for 28S ribosomal RNA, partial sequence, clone: KBM6-8
denovo_8579	KF849683.1	-	-	-	-	97.76	713	98.63	659	Uncultured *Glomus* clone AM203 18S ribosomal RNA gene, partial sequence; internal transcribed spacer 1, 5.8S ribosomal RNA gene, and internal transcribed spacer 2, complete sequence; and 28S ribosomal RNA gene, partial sequence
denovo_8715	KM208490.1	-	-	-	-	95.07	690	94.94	672	Uncultured Glomeromycota clone SHN2_137 18S ribosomal RNA gene, partial sequence; internal transcribed spacer 1, 5.8S ribosomal RNA gene, and internal transcribed spacer 2, complete sequence; and 28S ribosomal RNA gene, partial sequence
denovo_8728	KF849683.1	99.08	649	-	-	98.39	622	97.82	595	Uncultured *Glomus* clone AM203 18S ribosomal RNA gene, partial sequence; internal transcribed spacer 1, 5.8S ribosomal RNA gene, and internal transcribed spacer 2, complete sequence; and 28S ribosomal RNA gene, partial sequence
denovo_8743	JQ029749.1	-	-	-	-	96.63	178	96.22	715	Uncultured *Glomus* clone 44 large subunit ribosomal RNA gene, partial sequence
denovo_8921	KF849683.1	98.29	585	-	-	97.72	657	97.72	657	Uncultured *Glomus* clone AM203 18S ribosomal RNA gene, partial sequence; internal transcribed spacer 1, 5.8S ribosomal RNA gene, and internal transcribed spacer 2, complete sequence; and 28S ribosomal RNA gene, partial sequence
denovo_9000	KF849683.1	-	-	-	-	-	-	98.71	697	Uncultured *Glomus* clone AM203 18S ribosomal RNA gene, partial sequence; internal transcribed spacer 1, 5.8S ribosomal RNA gene, and internal transcribed spacer 2, complete sequence; and 28S ribosomal RNA gene, partial sequence
denovo_9002	KF849683.1	-	-	-	-	-	-	96.82	692	Uncultured *Glomus* clone AM203 18S ribosomal RNA gene, partial sequence; internal transcribed spacer 1, 5.8S ribosomal RNA gene, and internal transcribed spacer 2, complete sequence; and 28S ribosomal RNA gene, partial sequence
denovo_9003	KF849683.1	-	-	-	-	-	-	98.91	639	Uncultured *Glomus* clone AM203 18S ribosomal RNA gene, partial sequence; internal transcribed spacer 1, 5.8S ribosomal RNA gene, and internal transcribed spacer 2, complete sequence; and 28S ribosomal RNA gene, partial sequence
denovo_9011	KF849683.1	-	-	-	-	-	-	98.70	690	Uncultured *Glomus* clone AM203 18S ribosomal RNA gene, partial sequence; internal transcribed spacer 1, 5.8S ribosomal RNA gene, and internal transcribed spacer 2, complete sequence; and 28S ribosomal RNA gene, partial sequence
denovo_9050	KF849683.1	-	-	-	-	-	-	98.99	690	Uncultured *Glomus* clone AM203 18S ribosomal RNA gene, partial sequence; internal transcribed spacer 1, 5.8S ribosomal RNA gene, and internal transcribed spacer 2, complete sequence; and 28S ribosomal RNA gene, partial sequence
denovo_9089	KM208490.1	-	-	-	-	-	-	98.20	501	Uncultured Glomeromycota clone SHN2_137 18S ribosomal RNA gene, partial sequence; internal transcribed spacer 1, 5.8S ribosomal RNA gene, and internal transcribed spacer 2, complete sequence; and 28S ribosomal RNA gene, partial sequence
denovo_9129	KM208490.1	-	-	-	-	-	-	98.27	519	Uncultured Glomeromycota clone SHN2_137 18S ribosomal RNA gene, partial sequence; internal transcribed spacer 1, 5.8S ribosomal RNA gene, and internal transcribed spacer 2, complete sequence; and 28S ribosomal RNA gene, partial sequence
denovo_9162	KC411217.1	-	-	-	-	-	-	90.56	699	Uncultured Glomerales clone B04_50 large subunit ribosomal RNA gene, partial sequence
denovo_919	HF970216.1	98.40	625	98.64	589	-	-	-	-	Uncultured *Archaeospora* genomic DNA containing 18S rRNA gene, ITS1, 5.8S rRNA gene, ITS2 and 28S rRNA gene, clone CS030-02
denovo_9275	KM208489.1	-	-	-	-	-	-	90.31	712	Uncultured Glomeromycota clone SHN2_122 18S ribosomal RNA gene, partial sequence; internal transcribed spacer 1, 5.8S ribosomal RNA gene, and internal transcribed spacer 2, complete sequence; and 28S ribosomal RNA gene, partial sequence
denovo_930	KC411147.1	-	-	92.80	708	-	-	-	-	Uncultured Glomerales clone H19_29 large subunit ribosomal RNA gene, partial sequence
denovo_998	KC411147.1	-	-	93.99	682	94.26	610	-	-	Uncultured Glomerales clone H19_29 large subunit ribosomal RNA gene, partial sequence

Table S3. List of the IDs of "Known" taxa (identified by LSU rDNA database) and "*de novo*" taxa that were in common between different soils (Bs or Rs) and in different sampling times (1S or 2S).

41 common element in "Bs1S" and "Rs1S":	
**Taxa ID**	**Identification by LSU rDNA database (for "known" taxa) or by BLAST against NCBI (for "denovo" taxa)**
AJ854611	*Glomus* sp. MUCL 43205 28S rRNA gene, strain MUCL 43205, clone 11
AJ854619	*Glomus* sp. MUCL 43206 28S rRNA gene, strain MUCL 43206, clone 7
AJ854620	*Glomus* sp. MUCL 43206 28S rRNA gene, strain MUCL 43206, clone 8
AJ854623	*Glomus* sp. MUCL 43207 28S rRNA gene, strain MUCL 43207, clone 1
AJ854625	*Glomus* sp. MUCL 43207 28S rRNA gene, strain MUCL 43207, clone 3
AJ854629	*Glomus* sp. MUCL 43207 28S rRNA gene, strain MUCL 43207, clone 7
AY639205	*Rhizophagus* cf. *intraradices* HG-2010 clone 34.1 28S large subunit ribosomal RNA gene, partial sequence
AY639214	*Rhizophagus* cf. *intraradices* HG-2010 isolate 57 clone 57.7.1 28S large subunit ribosomal RNA gene, partial sequence
AY639218	*Rhizophagus* cf. *intraradices* HG-2010 isolate 107 clone 107.3.1 28S large subunit ribosomal RNA gene, partial sequence
AY639219	*Rhizophagus* cf. *intraradices* HG-2010 isolate 107 clone 107.3.3 28S large subunit ribosomal RNA gene, partial sequence
AY639294	*Rhizophagus* cf. *intraradices* HG-2010 clone 28.1 28S large subunit ribosomal RNA gene, partial sequence
AY639299	*Rhizophagus* cf. *intraradices* HG-2010 isolate 57 clone 57.6.3 28S large subunit ribosomal RNA gene, partial sequence
AY639300	*Rhizophagus* cf. *intraradices* HG-2010 isolate 57 clone 57.7.2 28S large subunit ribosomal RNA gene, partial sequence
AY639301	*Rhizophagus* cf. *intraradices* HG-2010 isolate 57 clone 57.7.3 28S large subunit ribosomal RNA gene, partial sequence
AY639303	*Rhizophagus* cf. *intraradices* HG-2010 isolate 58 clone 58.1.3 28S large subunit ribosomal RNA gene, partial sequence
Fi1cons#33	Uncultured *Rhizophagus* partial 28S RRNA gene , OTU AMF_C019Rhirr
FR750116	*Rhizophagus* cf. *irregularis* MUCL 43205 18S rRNA gene (partial), ITS1, 5.8S rRNA gene, ITS2 and 28S rRNA gene (partial), isolate MUCL43205, clone pHS058-6
Gintra691BEG144	*Glomus _intraradices* _691_BEG144
Gintx99640BEG141	*Glomus _intraradices* _691_BEG141
HF968919	*Rhizophagus irregularis* genomic DNA containing 18S rRNA gene, ITS1, 5.8S rRNA gene, ITS2 and 28S rRNA gene, strain DAOM181602, isolate spore 1, clone EI2_3_8
JF439167	*Glomus constrictum* isolate 08_48_12 18S ribosomal RNA gene, partial sequence; internal transcribed spacer 1, 5.8S ribosomal RNA gene, and internal transcribed spacer 2, complete sequence; and 28S ribosomal RNA gene, partial sequence
JN417518	*Rhizophagus irregularis* clone 2.4 18S ribosomal RNA gene, partial sequence; internal transcribed spacer 1, 5.8S ribosomal RNA gene, and internal transcribed spacer 2, complete sequence; and 28S ribosomal RNA gene, partial sequence
KC182036	*Septoglomus viscosum* clone HSp82-2 18S ribosomal RNA gene, partial sequence; internal transcribed spacer 1, 5.8S ribosomal RNA gene, and internal transcribed spacer 2, complete sequence; and 28S ribosomal RNA gene, partial sequence

KC182037	*Septoglomus viscosum* clone HSp82-9 18S ribosomal RNA gene, partial sequence; internal transcribed spacer 1, 5.8S ribosomal RNA gene, and internal transcribed spacer 2, complete sequence; and 28S ribosomal RNA gene, partial sequence
denovo_1	Uncultured *Glomus* clone AM203 18S ribosomal RNA gene, partial sequence; internal transcribed spacer 1, 5.8S ribosomal RNA gene, and internal transcribed spacer 2, complete sequence; and 28S ribosomal RNA gene, partial sequence
denovo_1440	Uncultured *Glomus* clone AM203 18S ribosomal RNA gene, partial sequence; internal transcribed spacer 1, 5.8S ribosomal RNA gene, and internal transcribed spacer 2, complete sequence; and 28S ribosomal RNA gene, partial sequence
denovo_1447	Uncultured *Rhizophagus* genomic DNA containing 18S rRNA gene, ITS1, 5.8S rRNA gene, ITS2 and 28S rRNA gene, clone JavorIX1
denovo_1448	Uncultured *Glomus* clone ZHwq2-238 18S ribosomal RNA gene, partial sequence; internal transcribed spacer 1, 5.8S ribosomal RNA gene, and internal transcribed spacer 2, complete sequence; and 28S ribosomal RNA gene, partial sequence
denovo_1452	Uncultured *Glomus* clone AM203 18S ribosomal RNA gene, partial sequence; internal transcribed spacer 1, 5.8S ribosomal RNA gene, and internal transcribed spacer 2, complete sequence; and 28S ribosomal RNA gene, partial sequence
denovo_1456	Uncultured *Glomus* clone AM203 18S ribosomal RNA gene, partial sequence; internal transcribed spacer 1, 5.8S ribosomal RNA gene, and internal transcribed spacer 2, complete sequence; and 28S ribosomal RNA gene, partial sequence
denovo_1460	*Glomus* sp. 2 SL-2017 isolate SR6 clone 1 18S ribosomal RNA gene, partial sequence; internal transcribed spacer 1, 5.8S ribosomal RNA gene, and internal transcribed spacer 2, complete sequence; and 28S ribosomal RNA gene, partial sequence
denovo_1482	Uncultured *Glomus* partial 28S rRNA gene, clone FW5-11
denovo_1489	Uncultured *Glomus* clone AM203 18S ribosomal RNA gene, partial sequence; internal transcribed spacer 1, 5.8S ribosomal RNA gene, and internal transcribed spacer 2, complete sequence; and 28S ribosomal RNA gene, partial sequence
denovo_1511	Uncultured *Glomus* partial 28S rRNA gene, clone FW5-11
denovo_1621	Uncultured Glomus partial 28S rRNA gene, clone BROS3-13
denovo_1633	Uncultured *Rhizophagus* genomic DNA containing 18S rRNA gene, ITS1, 5.8S rRNA gene, ITS2 and 28S rRNA gene, clone JavorIX1
denovo_2	Uncultured *Glomus* clone AM203 18S ribosomal RNA gene, partial sequence; internal transcribed spacer 1, 5.8S ribosomal RNA gene, and internal transcribed spacer 2, complete sequence; and 28S ribosomal RNA gene, partial sequence
denovo_27	Uncultured *Glomus* clone AM203 18S ribosomal RNA gene, partial sequence; internal transcribed spacer 1, 5.8S ribosomal RNA gene, and internal transcribed spacer 2, complete sequence; and 28S ribosomal RNA gene, partial sequence
denovo_479	Uncultured Glomerales clone H19_29 large subunit ribosomal RNA gene, partial sequence
denovo_500	Uncultured Glomerales clone H19_29 large subunit ribosomal RNA gene, partial sequence
denovo_552	Uncultured Glomerales clone H19_29 large subunit ribosomal RNA gene, partial sequence
**44 common elements in "Bs2S" and "Rs2S":**	
**Taxa ID**	**Identification by LSU rDNA database (for "known" taxa) or by BLAST against NCBI (for "denovo" taxa)**
AJ854594	*Glomus* sp. MUCL 43204 28S rRNA gene, strain MUCL 43204, clone 1
AJ854606	*Glomus* sp. MUCL 43205 28S rRNA gene, strain MUCL 43205, clone 6
AJ854611	*Glomus* sp. MUCL 43205 28S rRNA gene, strain MUCL 43205, clone 11

AJ854615	*Glomus* sp. MUCL 43206 28S rRNA gene, strain MUCL 43206, clone 3
AJ854619	*Glomus* sp. MUCL 43206 28S rRNA gene, strain MUCL 43206, clone 7
AJ854623	*Glomus* sp. MUCL 43207 28S rRNA gene, strain MUCL 43207, clone 1
AJ854625	*Glomus* sp. MUCL 43207 28S rRNA gene, strain MUCL 43207, clone 3
AJ854626	*Glomus* sp. MUCL 43207 28S rRNA gene, strain MUCL 43207, clone 4
AY639205	*Rhizophagus* cf. *intraradices* HG-2010 clone 34.1 28S large subunit ribosomal RNA gene, partial sequence
AY639208	*Rhizophagus* cf. *intraradices* HG-2010 clone 42.2 28S large subunit ribosomal RNA gene, partial sequence
AY639214	*Rhizophagus* cf. *intraradices* HG-2010 isolate 57 clone 57.7.1 28S large subunit ribosomal RNA gene, partial sequence
AY639216	*Rhizophagus* cf. *intraradices* HG-2010 isolate 58 clone 58.1.4 28S large subunit ribosomal RNA gene, partial sequence
AY639218	*Rhizophagus* cf. *intraradices* HG-2010 isolate 107 clone 107.3.1 28S large subunit ribosomal RNA gene, partial sequence
AY639219	*Rhizophagus* cf. *intraradices* HG-2010 isolate 107 clone 107.3.3 28S large subunit ribosomal RNA gene, partial sequence
AY639294	*Rhizophagus* cf. *intraradices* HG-2010 clone 28.1 28S large subunit ribosomal RNA gene, partial sequence
AY639299	*Rhizophagus* cf. *intraradices* HG-2010 isolate 57 clone 57.6.3 28S large subunit ribosomal RNA gene, partial sequence
AY639301	*Rhizophagus* cf. *intraradices* HG-2010 isolate 57 clone 57.7.3 28S large subunit ribosomal RNA gene, partial sequence
DQ469115	*Glomus* cf. *diaphanum* 589 clone 589.3 28S large subunit ribosomal RNA gene, partial sequence
Fi1cons#33	Uncultured *Rhizophagus* partial 28S RRNA gene , OTU AMF_C019Rhirr
FR750116	*Rhizophagus* cf. *irregularis* MUCL 43205 18S rRNA gene (partial), ITS1, 5.8S rRNA gene, ITS2 and 28S rRNA gene (partial), isolate MUCL43205, clone pHS058-6
FR750190	*Rhizophagus irregularis* 18S rRNA gene (partial), ITS1, 5.8S rRNA gene, ITS2 and 28S rRNA gene (partial), isolate Att857-12, clone pMK100-7
Gintra691BEG144	*Glomus* _*intraradices* _691_BEG144
HF968919	*Rhizophagus irregularis* genomic DNA containing 18S rRNA gene, ITS1, 5.8S rRNA gene, ITS2 and 28S rRNA gene, strain DAOM181602, isolate spore 1, clone EI2_3_8
HM625892	*Glomus intraradices* isolate intra6 28S ribosomal RNA gene, partial sequence
JF439161	*Glomus* sp. 9 SUN-2011 isolate 08_48_1 18S ribosomal RNA gene, partial sequence; internal transcribed spacer 1, 5.8S ribosomal RNA gene, and internal transcribed spacer 2, complete sequence; and 28S ribosomal RNA gene, partial sequence
JN417519	*Rhizophagus irregularis* clone 2.18 18S ribosomal RNA gene, partial sequence; internal transcribed spacer 1, 5.8S ribosomal RNA gene, and internal transcribed spacer 2, complete sequence; and 28S ribosomal RNA gene, partial sequence
denovo_1	Uncultured *Glomus* clone AM203 18S ribosomal RNA gene, partial sequence; internal transcribed spacer 1, 5.8S ribosomal RNA gene, and internal transcribed spacer 2, complete sequence; and 28S ribosomal RNA gene, partial sequence
denovo_2	Uncultured *Glomus* clone AM203 18S ribosomal RNA gene, partial sequence; internal transcribed spacer 1, 5.8S ribosomal RNA gene, and internal transcribed spacer 2, complete sequence; and 28S ribosomal RNA gene, partial sequence
denovo_27	Uncultured *Glomus* clone AM203 18S ribosomal RNA gene, partial sequence; internal transcribed spacer 1, 5.8S ribosomal RNA gene, and internal transcribed spacer 2, complete sequence; and 28S ribosomal RNA gene, partial sequence

denovo_344	Uncultured *Glomus* clone AM203 18S ribosomal RNA gene, partial sequence; internal transcribed spacer 1, 5.8S ribosomal RNA gene, and internal transcribed spacer 2, complete sequence; and 28S ribosomal RNA gene, partial sequence
denovo_381	Uncultured *Glomus* partial 28S rRNA gene, clone FW4-2
denovo_4138	Uncultured Glomerales clone H19_29 large subunit ribosomal RNA gene, partial sequence
denovo_4139	Uncultured Glomerales clone H19_29 large subunit ribosomal RNA gene, partial sequence
denovo_4298	Uncultured Glomeromycota clone 6.11 28S large subunit ribosomal RNA gene, partial sequence
denovo_4474	Uncultured Glomerales clone H19_29 large subunit ribosomal RNA gene, partial sequence
denovo_4480	Uncultured *Glomus* gene for 28S ribosomal RNA, partial sequence, clone: KBM6-8
denovo_4823	Uncultured *Glomus* gene for 28S ribosomal RNA, partial sequence, clone: KBM6-8
denovo_500	Uncultured Glomerales clone H19_29 large subunit ribosomal RNA gene, partial sequence
denovo_507	*Rhizophagus irregularis* 18S rRNA gene (partial), ITS1, 5.8S rRNA gene, ITS2 and 28S rRNA gene (partial), isolate MUCL43195, clone pHS037-4
denovo_552	Uncultured Glomerales clone H19_29 large subunit ribosomal RNA gene, partial sequence
denovo_634	*Glomus* sp. MUCL 43205 28S rRNA gene, strain MUCL 43205, clone 2
denovo_6490	*Glomus* sp. MUCL 43205 28S rRNA gene, strain MUCL 43205, clone 11
denovo_6512	Glomeromycota sp. OTU3 DJMC-2012 isolate spore 1_9_2 28S ribosomal RNA gene, partial sequence
denovo_8007	*Glomus* sp. 2 SL-2017 isolate SR6 clone 1 18S ribosomal RNA gene, partial sequence; internal transcribed spacer 1, 5.8S ribosomal RNA gene, and internal transcribed spacer 2, complete sequence; and 28S ribosomal RNA gene, partial sequence

43 common elements in "Bs1S" and "Bs2S":	
**Taxa ID**	**Identification by LSU rDNA database (for "known" taxa) or by BLAST against NCBI (for "denovo" taxa)**
AF145743	*Glomus geosporum* strain BEG 106 large subunit ribosomal RNA gene, partial sequence
AJ854611	*Glomus* sp. MUCL 43205 28S rRNA gene, strain MUCL 43205, clone 11
AJ854619	*Glomus* sp. MUCL 43206 28S rRNA gene, strain MUCL 43206, clone 7
AJ854623	*Glomus* sp. MUCL 43207 28S rRNA gene, strain MUCL 43207, clone 1
AJ854625	*Glomus* sp. MUCL 43207 28S rRNA gene, strain MUCL 43207, clone 3
AY639205	*Rhizophagus* cf. *intraradices* HG-2010 clone 34.1 28S large subunit ribosomal RNA gene, partial sequence
AY639214	*Rhizophagus* cf. *intraradices* HG-2010 isolate 57 clone 57.7.1 28S large subunit ribosomal RNA gene, partial sequence
AY639218	*Rhizophagus* cf. *intraradices* HG-2010 isolate 107 clone 107.3.1 28S large subunit ribosomal RNA gene, partial sequence
AY639219	*Rhizophagus* cf. *intraradices* HG-2010 isolate 107 clone 107.3.3 28S large subunit ribosomal RNA gene, partial sequence
AY639294	*Rhizophagus* cf. *intraradices* HG-2010 clone 28.1 28S large subunit ribosomal RNA gene, partial sequence
AY639299	*Rhizophagus* cf. *intraradices* HG-2010 isolate 57 clone 57.6.3 28S large subunit ribosomal RNA gene, partial sequence
AY639301	*Rhizophagus* cf. *intraradices* HG-2010 isolate 57 clone 57.7.3 28S large subunit ribosomal RNA gene, partial sequence
Fi1cons#33	Uncultured *Rhizophagus* partial 28S RRNA gene , OTU AMF_C019Rhirr

FR750116	*Rhizophagus* cf. *irregularis* MUCL 43205 18S rRNA gene (partial), ITS1, 5.8S rRNA gene, ITS2 and 28S rRNA gene (partial), isolate MUCL43205, clone pHS058-6
Gintra691BEG144	*Glomus _intraradices* _691_BEG144
HF968919	*Rhizophagus irregularis* genomic DNA containing 18S rRNA gene, ITS1, 5.8S rRNA gene, ITS2 and 28S rRNA gene, strain DAOM181602, isolate spore 1, clone EI2_3_8
JF439161	*Glomus* sp. 9 SUN-2011 isolate 08_48_1 18S ribosomal RNA gene, partial sequence; internal transcribed spacer 1, 5.8S ribosomal RNA gene, and internal transcribed spacer 2, complete sequence; and 28S ribosomal RNA gene, partial sequence
JQ048895	*Funneliformis geosporum* strain BEG11 clone Hsp40-13b 28S ribosomal RNA (LSU) gene, partial sequence
denovo_1	Uncultured *Glomus* clone AM203 18S ribosomal RNA gene, partial sequence; internal transcribed spacer 1, 5.8S ribosomal RNA gene, and internal transcribed spacer 2, complete sequence; and 28S ribosomal RNA gene, partial sequence
denovo_137	Uncultured *Glomus* clone AM203 18S ribosomal RNA gene, partial sequence; internal transcribed spacer 1, 5.8S ribosomal RNA gene, and internal transcribed spacer 2, complete sequence; and 28S ribosomal RNA gene, partial sequence
denovo_2	Uncultured *Glomus* clone AM203 18S ribosomal RNA gene, partial sequence; internal transcribed spacer 1, 5.8S ribosomal RNA gene, and internal transcribed spacer 2, complete sequence; and 28S ribosomal RNA gene, partial sequence
denovo_27	Uncultured *Glomus* clone AM203 18S ribosomal RNA gene, partial sequence; internal transcribed spacer 1, 5.8S ribosomal RNA gene, and internal transcribed spacer 2, complete sequence; and 28S ribosomal RNA gene, partial sequence
denovo_2749	*Glomus macrocarpum* isolate MD124 25S ribosomal RNA gene, partial sequence
denovo_3223	Uncultured *Archaeospora* genomic DNA containing 18S rRNA gene, ITS1, 5.8S rRNA gene, ITS2 and 28S rRNA gene, clone CS030-02
denovo_344	Uncultured *Glomus* clone AM203 18S ribosomal RNA gene, partial sequence; internal transcribed spacer 1, 5.8S ribosomal RNA gene, and internal transcribed spacer 2, complete sequence; and 28S ribosomal RNA gene, partial sequence
denovo_358	Uncultured *Archaeospora* genomic DNA containing 18S rRNA gene, ITS1, 5.8S rRNA gene, ITS2 and 28S rRNA gene, clone CS030-02
denovo_381	Uncultured *Glomus* partial 28S rRNA gene, clone FW4-2
denovo_4119	*Glomus macrocarpum* isolate MD124 25S ribosomal RNA gene, partial sequence
denovo_4138	Uncultured Glomerales clone H19_29 large subunit ribosomal RNA gene, partial sequence
denovo_4139	Uncultured Glomerales clone H19_29 large subunit ribosomal RNA gene, partial sequence
denovo_4298	Uncultured Glomeromycota clone 6.11 28S large subunit ribosomal RNA gene, partial sequence
denovo_4474	Uncultured Glomerales clone H19_29 large subunit ribosomal RNA gene, partial sequence
denovo_479	Uncultured Glomerales clone H19_29 large subunit ribosomal RNA gene, partial sequence
denovo_493	Uncultured Glomerales clone B04_41 large subunit ribosomal RNA gene, partial sequence
denovo_500	Uncultured Glomerales clone H19_29 large subunit ribosomal RNA gene, partial sequence
denovo_506	Uncultured *Glomus* partial 28S rRNA gene, clone BROS1-19
denovo_552	Uncultured Glomerales clone H19_29 large subunit ribosomal RNA gene, partial sequence
denovo_561	Uncultured *Archaeospora* genomic DNA containing 18S rRNA gene, ITS1, 5.8S rRNA gene, ITS2 and 28S rRNA gene, clone CS030-02

denovo_581	Uncultured *Archaeospora* genomic DNA containing 18S rRNA gene, ITS1, 5.8S rRNA gene, ITS2 and 28S rRNA gene, clone CS030-02
denovo_627	Uncultured *Archaeospora* genomic DNA containing 18S rRNA gene, ITS1, 5.8S rRNA gene, ITS2 and 28S rRNA gene, clone CS030-02
denovo_677	Uncultured *Archaeospora* genomic DNA containing 18S rRNA gene, ITS1, 5.8S rRNA gene, ITS2 and 28S rRNA gene, clone CS030-02
denovo_711	Uncultured *Glomus* partial 28S rRNA gene, clone WW2-2
denovo_843	Uncultured Glomeromycota clone 4_16 18S ribosomal RNA gene, partial sequence; internal transcribed spacer 1, 5.8S ribosomal RNA gene, and internal transcribed spacer 2, complete sequence; and 28S ribosomal RNA gene, partial sequence

57 common elements in "Rs1S" and "Rs2S":

**Taxa ID**	**Identification by LSU rDNA database (for "known" taxa) or by BLAST against NCBI (for "denovo" taxa)**
AJ854594	*Glomus* sp. MUCL 43204 28S rRNA gene, strain MUCL 43204, clone 1
AJ854602	*Glomus* sp. MUCL 43205 28S rRNA gene, strain MUCL 43205, clone 2
AJ854606	*Glomus* sp. MUCL 43205 28S rRNA gene, strain MUCL 43205, clone 6
AJ854611	*Glomus* sp. MUCL 43205 28S rRNA gene, strain MUCL 43205, clone 11
AJ854619	*Glomus* sp. MUCL 43206 28S rRNA gene, strain MUCL 43206, clone 7
AJ854620	*Glomus* sp. MUCL 43206 28S rRNA gene, strain MUCL 43206, clone 8
AJ854622	*Glomus* sp. MUCL 43206 28S rRNA gene, strain MUCL 43206, clone 10
AJ854623	*Glomus* sp. MUCL 43207 28S rRNA gene, strain MUCL 43207, clone 1
AJ854625	*Glomus* sp. MUCL 43207 28S rRNA gene, strain MUCL 43207, clone 3
AY639205	*Rhizophagus* cf. *intraradices* HG-2010 clone 34.1 28S large subunit ribosomal RNA gene, partial sequence
AY639208	*Rhizophagus* cf. *intraradices* HG-2010 clone 42.2 28S large subunit ribosomal RNA gene, partial sequence
AY639214	*Rhizophagus* cf. *intraradices* HG-2010 isolate 57 clone 57.7.1 28S large subunit ribosomal RNA gene, partial sequence
AY639216	*Rhizophagus* cf. *intraradices* HG-2010 isolate 58 clone 58.1.4 28S large subunit ribosomal RNA gene, partial sequence
AY639218	*Rhizophagus* cf. *intraradices* HG-2010 isolate 107 clone 107.3.1 28S large subunit ribosomal RNA gene, partial sequence
AY639219	*Rhizophagus* cf. *intraradices* HG-2010 isolate 107 clone 107.3.3 28S large subunit ribosomal RNA gene, partial sequence
AY639294	*Rhizophagus* cf. *intraradices* HG-2010 clone 28.1 28S large subunit ribosomal RNA gene, partial sequence
AY639299	*Rhizophagus* cf. *intraradices* HG-2010 isolate 57 clone 57.6.3 28S large subunit ribosomal RNA gene, partial sequence
AY639300	*Rhizophagus* cf. *intraradices* HG-2010 isolate 57 clone 57.7.2 28S large subunit ribosomal RNA gene, partial sequence
AY639301	*Rhizophagus* cf. *intraradices* HG-2010 isolate 57 clone 57.7.3 28S large subunit ribosomal RNA gene, partial sequence
AY639303	*Rhizophagus* cf. *intraradices* HG-2010 isolate 58 clone 58.1.3 28S large subunit ribosomal RNA gene, partial sequence
DQ469115	*Glomus* cf. *diaphanum* 589 clone 589.3 28S large subunit ribosomal RNA gene, partial sequence
Fi1cons#33	Uncultured *Rhizophagus* partial 28S RRNA gene , OTU AMF_C019Rhirr
FR750116	*Rhizophagus* cf. *irregularis* MUCL 43205 18S rRNA gene (partial), ITS1, 5.8S rRNA gene, ITS2 and 28S rRNA gene (partial), isolate MUCL43205, clone pHS058-6
FR750190	*Rhizophagus irregularis* 18S rRNA gene (partial), ITS1, 5.8S rRNA gene, ITS2 and 28S rRNA gene (partial), isolate Att857-12, clone pMK100-7

Gintra691BEG144	*Glomus* _*intraradices* _691_BEG144
HE794038	*Glomus* sp. PB genomic DNA containing 18S rRNA gene, ITS1, 5.8S rRNA gene, ITS2 and 28S rRNA gene, strain PB, clone pb1
HF968919	*Rhizophagus irregularis* genomic DNA containing 18S rRNA gene, ITS1, 5.8S rRNA gene, ITS2 and 28S rRNA gene, strain DAOM181602, isolate spore 1, clone EI2_3_8
JN417518	*Rhizophagus irregularis* clone 2.4 18S ribosomal RNA gene, partial sequence; internal transcribed spacer 1, 5.8S ribosomal RNA gene, and internal transcribed spacer 2, complete sequence; and 28S ribosomal RNA gene, partial sequence
JN417519	*Rhizophagus irregularis* clone 2.18 18S ribosomal RNA gene, partial sequence; internal transcribed spacer 1, 5.8S ribosomal RNA gene, and internal transcribed spacer 2, complete sequence; and 28S ribosomal RNA gene, partial sequence
denovo_1	Uncultured *Glomus* clone AM203 18S ribosomal RNA gene, partial sequence; internal transcribed spacer 1, 5.8S ribosomal RNA gene, and internal transcribed spacer 2, complete sequence; and 28S ribosomal RNA gene, partial sequence
denovo_10042	Uncultured *Glomus* clone 44 large subunit ribosomal RNA gene, partial sequence
denovo_10180	Uncultured *Glomus* clone 44 large subunit ribosomal RNA gene, partial sequence
denovo_13922	Uncultured *Glomus* clone 44 large subunit ribosomal RNA gene, partial sequence
denovo_1440	Uncultured *Glomus* clone AM203 18S ribosomal RNA gene, partial sequence; internal transcribed spacer 1, 5.8S ribosomal RNA gene, and internal transcribed spacer 2, complete sequence; and 28S ribosomal RNA gene, partial sequence
denovo_1447	Uncultured *Rhizophagus* genomic DNA containing 18S rRNA gene, ITS1, 5.8S rRNA gene, ITS2 and 28S rRNA gene, clone JavorIX1
denovo_1448	Uncultured *Glomus* clone ZHwq2-238 18S ribosomal RNA gene, partial sequence; internal transcribed spacer 1, 5.8S ribosomal RNA gene, and internal transcribed spacer 2, complete sequence; and 28S ribosomal RNA gene, partial sequence
denovo_1452	Uncultured *Glomus* clone AM203 18S ribosomal RNA gene, partial sequence; internal transcribed spacer 1, 5.8S ribosomal RNA gene, and internal transcribed spacer 2, complete sequence; and 28S ribosomal RNA gene, partial sequence
denovo_1456	Uncultured *Glomus* clone AM203 18S ribosomal RNA gene, partial sequence; internal transcribed spacer 1, 5.8S ribosomal RNA gene, and internal transcribed spacer 2, complete sequence; and 28S ribosomal RNA gene, partial sequence
denovo_1460	*Glomus* sp. 2 SL-2017 isolate SR6 clone 1 18S ribosomal RNA gene, partial sequence; internal transcribed spacer 1, 5.8S ribosomal RNA gene, and internal transcribed spacer 2, complete sequence; and 28S ribosomal RNA gene, partial sequence
denovo_1489	Uncultured *Glomus* clone AM203 18S ribosomal RNA gene, partial sequence; internal transcribed spacer 1, 5.8S ribosomal RNA gene, and internal transcribed spacer 2, complete sequence; and 28S ribosomal RNA gene, partial sequence
denovo_1627	Uncultured *Glomus* clone AM203 18S ribosomal RNA gene, partial sequence; internal transcribed spacer 1, 5.8S ribosomal RNA gene, and internal transcribed spacer 2, complete sequence; and 28S ribosomal RNA gene, partial sequence
denovo_1633	Uncultured *Rhizophagus* genomic DNA containing 18S rRNA gene, ITS1, 5.8S rRNA gene, ITS2 and 28S rRNA gene, clone JavorIX1
denovo_1827	Uncultured *Glomus* clone AM203 18S ribosomal RNA gene, partial sequence; internal transcribed spacer 1, 5.8S ribosomal RNA gene, and internal transcribed spacer 2, complete sequence; and 28S ribosomal RNA gene, partial sequence
denovo_1994	Uncultured *Glomus* clone 18 large subunit ribosomal RNA gene, partial sequence

denovo_2	Uncultured *Glomus* clone AM203 18S ribosomal RNA gene, partial sequence; internal transcribed spacer 1, 5.8S ribosomal RNA gene, and internal transcribed spacer 2, complete sequence; and 28S ribosomal RNA gene, partial sequence
denovo_27	Uncultured *Glomus* clone AM203 18S ribosomal RNA gene, partial sequence; internal transcribed spacer 1, 5.8S ribosomal RNA gene, and internal transcribed spacer 2, complete sequence; and 28S ribosomal RNA gene, partial sequence
denovo_4480	Uncultured *Glomus* gene for 28S ribosomal RNA, partial sequence, clone: KBM6-8
denovo_4823	Uncultured *Glomus* gene for 28S ribosomal RNA, partial sequence, clone: KBM6-8
denovo_500	Uncultured Glomerales clone H19_29 large subunit ribosomal RNA gene, partial sequence
denovo_552	Uncultured Glomerales clone H19_29 large subunit ribosomal RNA gene, partial sequence
denovo_6085	Uncultured *Glomus* gene for 28S ribosomal RNA, partial sequence, clone: H5-2
denovo_6151	Uncultured *Glomus* clone 44 large subunit ribosomal RNA gene, partial sequence
denovo_628	*Glomus* sp. MUCL 43205 28S rRNA gene, strain MUCL 43205, clone 8
denovo_634	*Glomus* sp. MUCL 43205 28S rRNA gene, strain MUCL 43205, clone 2
denovo_6490	*Glomus* sp. MUCL 43205 28S rRNA gene, strain MUCL 43205, clone 11
denovo_6512	Glomeromycota sp. OTU3 DJMC-2012 isolate spore 1_9_2 28S ribosomal RNA gene, partial sequence
denovo_8728	Uncultured *Glomus* clone AM203 18S ribosomal RNA gene, partial sequence; internal transcribed spacer 1, 5.8S ribosomal RNA gene, and internal transcribed spacer 2, complete sequence; and 28S ribosomal RNA gene, partial sequence

20 common element in "Rs1S", "Rs2S", "Bs1S" and "Bs2S":	
**Taxa ID**	**Identification by LSU rDNA database (for "known" taxa) or by BLAST against NCBI (for "denovo" taxa)**
AJ854611	*Glomus* sp. MUCL 43205 28S rRNA gene, strain MUCL 43205, clone 11
AJ854619	*Glomus* sp. MUCL 43206 28S rRNA gene, strain MUCL 43206, clone 7
AJ854623	*Glomus* sp. MUCL 43207 28S rRNA gene, strain MUCL 43207, clone 1
AJ854625	*Glomus* sp. MUCL 43207 28S rRNA gene, strain MUCL 43207, clone 3
AY639205	*Rhizophagus* cf. *intraradices* HG-2010 clone 34.1 28S large subunit ribosomal RNA gene, partial sequence
AY639214	*Rhizophagus* cf. *intraradices* HG-2010 isolate 57 clone 57.7.1 28S large subunit ribosomal RNA gene, partial sequence
AY639218	*Rhizophagus* cf. *intraradices* HG-2010 isolate 107 clone 107.3.1 28S large subunit ribosomal RNA gene, partial sequence
AY639219	*Rhizophagus* cf. *intraradices* HG-2010 isolate 107 clone 107.3.3 28S large subunit ribosomal RNA gene, partial sequence
AY639294	*Rhizophagus* cf. *intraradices* HG-2010 clone 28.1 28S large subunit ribosomal RNA gene, partial sequence
AY639299	*Rhizophagus* cf. *intraradices* HG-2010 isolate 57 clone 57.6.3 28S large subunit ribosomal RNA gene, partial sequence
AY639301	*Rhizophagus* cf. *intraradices* HG-2010 isolate 57 clone 57.7.3 28S large subunit ribosomal RNA gene, partial sequence
Fi1cons#33	Uncultured *Rhizophagus* partial 28S RRNA gene , OTU AMF_C019Rhirr
FR750116	*Rhizophagus* cf. *irregularis* MUCL 43205 18S rRNA gene (partial), ITS1, 5.8S rRNA gene, ITS2 and 28S rRNA gene (partial), isolate MUCL43205, clone pHS058-6
Gintra691BEG144	*Glomus* _*intraradices* _691_BEG144

HF968919	*Rhizophagus irregularis* genomic DNA containing 18S rRNA gene, ITS1, 5.8S rRNA gene, ITS2 and 28S rRNA gene, strain DAOM181602, isolate spore 1, clone EI2_3_8
denovo_1	Uncultured *Glomus* clone AM203 18S ribosomal RNA gene, partial sequence; internal transcribed spacer 1, 5.8S ribosomal RNA gene, and internal transcribed spacer 2, complete sequence; and 28S ribosomal RNA gene, partial sequence
denovo_2	Uncultured *Glomus* clone AM203 18S ribosomal RNA gene, partial sequence; internal transcribed spacer 1, 5.8S ribosomal RNA gene, and internal transcribed spacer 2, complete sequence; and 28S ribosomal RNA gene, partial sequence
denovo_27	Uncultured *Glomus* clone AM203 18S ribosomal RNA gene, partial sequence; internal transcribed spacer 1, 5.8S ribosomal RNA gene, and internal transcribed spacer 2, complete sequence; and 28S ribosomal RNA gene, partial sequence
denovo_500	Uncultured Glomerales clone H19_29 large subunit ribosomal RNA gene, partial sequence
denovo_552	Uncultured Glomerales clone H19_29 large subunit ribosomal RNA gene, partial sequence

Table S4. Heat tree difference table reporting the results (p-values) of the non-parametric Wilcoxon Rank Sum test used to depict taxonomic differences between AMF communities. Significant taxa are highlighted in yellow.

"Bs1S" vs. "Bs2S"				
tax_name	log2_median_ratio	median_diff	mean_diff	wilcox_p_value
Rhizophagus_irregularis	-7.686212502	-0.029855627	-0.059005202	0.036145142
Glomus_sp	-6.293161893	-0.270662179	-0.16745776	0.095238095
denovo_681	Inf	0.000983445	0.000768194	0.105997548
denovo_479	-Inf	-0.000517264	-0.053423377	0.118797498
denovo_1442	Inf	0.001019665	0.029528671	0.157939311
denovo_2	Inf	0.025760793	0.060605183	0.161237592
Glomus_invermaium	0	0	0.038820985	0.179712495
Glomus_aggregatum	0	0	0.007560387	0.179712495
denovo_1440	0	0	0.006369718	0.179712495
denovo_1482	0	0	0.000487133	0.179712495
denovo_1489	0	0	0.001416253	0.179712495
denovo_1511	0	0	0.001465614	0.179712495
denovo_1633	0	0	0.000718653	0.179712495
denovo_1647	0	0	0.002345433	0.179712495
denovo_1835	0	0	0.000692307	0.179712495
denovo_1882	0	0	0.000117159	0.179712495
denovo_2141	0	0	0.00028701	0.179712495
denovo_2381	0	0	0.000359326	0.179712495
denovo_2489	0	0	0.000298785	0.179712495
denovo_2674	0	0	-0.044680899	0.179712495
denovo_2869	0	0	-0.000285403	0.179712495
denovo_498	0	0	-0.000309812	0.179712495
denovo_507	0	0	-0.00098574	0.179712495
denovo_508	0	0	-0.000387744	0.179712495
denovo_540	0	0	-0.000207645	0.179712495
denovo_558	0	0	-0.00031016	0.179712495
denovo_570	0	0	-0.000361941	0.179712495
denovo_6490	0	0	-0.00240796	0.179712495
denovo_6828	0	0	0.086962053	0.179712495
denovo_6832	0	0	0.008122518	0.179712495
denovo_6833	0	0	0.001992376	0.179712495
denovo_6835	0	0	0.004123174	0.179712495
denovo_6851	0	0	0.001631779	0.179712495
denovo_6899	0	0	0.001402309	0.179712495
denovo_6955	0	0	0.001959594	0.179712495
denovo_804	0	0	-0.00075195	0.179712495
denovo_930	0	0	-0.001583053	0.179712495
denovo_1726	Inf	0.000566171	0.003118734	0.265205393
denovo_634	-Inf	-0.000656944	-0.001122239	0.265205393
Septoglomus_viscosum	Inf	0.001019665	-0.003587118	0.332111978
denovo_381	2.56329806	0.000635015	-0.01753565	0.397614752
Septoglomus_constrictum	0	0	0.004161359	0.423710797
denovo_1452	0	0	0.003510262	0.423710797
denovo_1453	0	0	0.002519462	0.423710797
denovo_1456	0	0	0.003510262	0.423710797
denovo_1460	0	0	0.006256192	0.423710797
denovo_1493	0	0	0.000113234	0.423710797
denovo_1503	0	0	8.74E-05	0.423710797
denovo_1627	0	0	2.83E-05	0.423710797
denovo_1827	0	0	0.000113234	0.423710797

denovo_1841	0	0	2.83E-05	0.423710797
denovo_1903	0	0	-0.001334729	0.423710797
denovo_1994	0	0	8.49E-05	0.423710797
denovo_2031	0	0	0.000113234	0.423710797
denovo_212	0	0	0.000152866	0.423710797
denovo_2396	0	0	2.83E-05	0.423710797
denovo_2958	0	0	-0.000155864	0.423710797
denovo_4121	0	0	0.001604359	0.423710797
denovo_4124	0	0	0.004389284	0.423710797
denovo_4147	0	0	0.004116846	0.423710797
denovo_4499	0	0	0.000151355	0.423710797
denovo_4642	0	0	9.08E-05	0.423710797
denovo_4823	0	0	-0.001681776	0.423710797
denovo_4847	0	0	6.05E-05	0.423710797
denovo_4972	0	0	-0.00056899	0.423710797
denovo_5122	0	0	-5.17E-05	0.423710797
denovo_531	0	0	-0.000387047	0.423710797
denovo_603	0	0	-0.000103212	0.423710797
denovo_6080	0	0	0.000116533	0.423710797
denovo_6085	0	0	5.83E-05	0.423710797
denovo_6151	0	0	2.91E-05	0.423710797
denovo_628	0	0	-2.58E-05	0.423710797
denovo_6491	0	0	-0.000340225	0.423710797
denovo_6512	0	0	-0.001596441	0.423710797
denovo_6539	0	0	-0.000157027	0.423710797
denovo_6794	0	0	-2.62E-05	0.423710797
denovo_6815	0	0	-5.23E-05	0.423710797
denovo_8007	0	0	-0.000683222	0.423710797
denovo_8143	0	0	-0.000420444	0.423710797
denovo_8251	0	0	-0.000315333	0.423710797
denovo_8534	0	0	-0.000105111	0.423710797
denovo_8728	0	0	8.49E-05	0.423710797
denovo_8921	0	0	2.83E-05	0.423710797
denovo_998	0	0	-0.000311729	0.423710797
denovo_1035	0	0	-0.000480346	0.440686016
denovo_1621	0	0	0.004472422	0.440686016
denovo_2351	0	0	0.001544924	0.440686016
denovo_2505	0	0	0.000484704	0.440686016
denovo_4119	0	0	0.016960602	0.440686016
denovo_4298	0	0	-0.006598104	0.440686016
denovo_4639	0	0	-0.003185321	0.440686016
denovo_500	0	0	-0.005110572	0.440686016
denovo_525	0	0	-0.002566717	0.440686016
denovo_552	0	0	-0.003315551	0.440686016
denovo_553	0	0	-0.00022399	0.440686016
denovo_344	4.650760901	0.006238183	0.003287783	0.463343883
denovo_506	Inf	0.000582666	0.001122969	0.48017689
denovo_843	-Inf	-0.000516062	8.88E-05	0.48017689
Rhizophagus_sp	-Inf	-0.000129316	-0.005338627	0.503812429
Glomus_cf	0	0	0.00275315	0.607235544
denovo_3167	0	0	-0.000436518	0.607235544
denovo_4228	0	0	-0.000267205	0.607235544
denovo_561	-2.178225234	-0.002311729	-0.027207977	0.666430034
denovo_627	1.718655393	0.000295613	-0.005895141	0.672335808
denovo_1447	0	0	0.03437387	0.724081661
Glomus_intraradices_691_BI	0	0	-0.019856193	0.797169693
denovo_137	0	0	5.46E-05	0.797169693

denovo_1517	0	0	-0.000105351	0.797169693
denovo_3223	0	0	-7.16E-05	0.797169693
denovo_4210	0	0	-0.001125895	0.797169693
denovo_4932	0	0	-0.000514548	0.797169693
Rhizophagus_intraradices	-Inf	-0.000258031	0.125605669	0.823664389
denovo_1	Inf	0.011351597	0.086514392	0.823664389
denovo_919	0.175122732	1.67E-05	-0.000596274	0.829356961
denovo_358	-1.841936251	-0.048513492	-0.034213278	0.841269841
denovo_2749	0	0	0.000300945	0.906329391
denovo_581	1.189745123	0.002975131	-0.003400868	0.916562645
Rhizophagus_cf	1.046237611	0.000826435	-0.02620479	1
Funneliformis_geosporum	0	0	0.010729632	1
Sclerocystis_sinuosa_MD12(	0	0	0.00639507	1
Glomus_intraradices_x99640	0	0	0.005069264	1
Claroideoglomus_drummond	0	0	-0.001465473	1
denovo_1448	0	0	0.002156342	1
denovo_1988	0	0	-0.001356625	1
denovo_209	0	0	0.000381666	1
denovo_27	1.715301153	0.000590625	-0.001662705	1
denovo_4138	0	0	-0.001286198	1
denovo_4139	0	0	-0.000216148	1
denovo_4162	0	0	0.004682221	1
denovo_4184	0	0	-0.000927335	1
denovo_4199	0	0	-0.000318007	1
denovo_4418	0	0	3.44E-05	1
denovo_4474	0	0	-0.001792169	1
denovo_4480	0	0	-0.083767818	1
denovo_49	0	0	7.45E-05	1
denovo_493	0	0	0.004073727	1
denovo_4961	0	0	-0.000226228	1
denovo_677	1.220675726	0.000172822	-0.004143271	1
denovo_711	0	0	0.002845014	1

"Rs1S" vs. "Rs2S"				
tax_name	log2_median_ratio	median_diff	mean_diff	wilcox_p_value
denovo_660	-Inf	-0.000518874	-0.000754763	0.007494958
Septoglomus_viscosum	Inf	0.00089172	0.047781219	0.02536986
denovo_10711	-Inf	-0.000639468	-0.001247872	0.02536986
denovo_570	-Inf	-0.000639468	-0.001140808	0.04490859
denovo_10732	-Inf	-0.000521853	-0.001169496	0.072005658
denovo_11975	-Inf	-0.000767362	-0.000646804	0.072005658
denovo_358	Inf	0.000126695	0.000153197	0.072005658
denovo_4162	-Inf	-0.000780031	-0.000779847	0.072005658
denovo_531	-Inf	-0.000129719	-0.000334944	0.072005658
denovo_4639	1.565444973	0.000254208	0.000967544	0.090688366
denovo_1903	Inf	0.001773724	0.00130634	0.105997548
Glomus_sp	0.592468097	0.100780374	0.113135024	0.150793651
denovo_1621	Inf	0.000255951	0.001911879	0.157939311
denovo_1647	-Inf	-0.000780031	-0.00045781	0.157939311
denovo_553	-Inf	-0.000390016	-0.000575779	0.157939311
denovo_10703	0	0	-0.001225758	0.179712495
denovo_10772	0	0	-0.00044283	0.179712495
denovo_10871	0	0	-0.000416454	0.179712495
denovo_11492	0	0	-0.000415959	0.179712495
denovo_11699	0	0	-0.000805172	0.179712495
denovo_13084	0	0	-0.000797685	0.179712495
denovo_1726	0	0	-0.014898481	0.179712495

denovo_4121	0	0	-0.000128738	0.179712495
denovo_4138	0	0	-0.030219312	0.179712495
denovo_4139	0	0	-0.000784733	0.179712495
denovo_4298	0	0	-0.005206355	0.179712495
denovo_4474	0	0	-0.000678675	0.179712495
denovo_4499	0	0	-0.002548282	0.179712495
denovo_4847	0	0	-0.001046458	0.179712495
denovo_498	0	0	-0.000181835	0.179712495
denovo_558	0	0	-0.000597737	0.179712495
denovo_9089	0	0	-0.000444818	0.179712495
Rhizophagus_sp	-Inf	-0.004305284	-0.001070204	0.265205393
denovo_6815	-Inf	-0.001023149	-0.000961287	0.265205393
Glomus_intraradices_691_BI	Inf	0.000511902	0.025377766	0.332111978
denovo_1447	-Inf	-0.004940198	-0.067382841	0.346521712
denovo_4480	Inf	0.000383926	0.011624286	0.346521712
denovo_634	-Inf	-0.001037748	-0.000504785	0.346521712
denovo_344	-2.734469739	-0.001432952	-0.04863242	0.388629493
Glomus_intraradices_x99640	0	0	0.005390539	0.423710797
Glomus_invermaium	0	0	-5.12E-05	0.423710797
Septoglomus_constrictum	0	0	0.003657866	0.423710797
denovo_1453	0	0	0.000247525	0.423710797
denovo_1482	0	0	0.001919631	0.423710797
denovo_1493	0	0	0.000412541	0.423710797
denovo_1503	0	0	-0.003198128	0.423710797
denovo_1511	0	0	0.000332736	0.423710797
denovo_2031	0	0	0.000330033	0.423710797
denovo_2381	0	0	0.001182618	0.423710797
denovo_2674	0	0	0.000819043	0.423710797
denovo_2958	0	0	-0.000518874	0.423710797
denovo_4124	0	0	-0.000181606	0.423710797
denovo_4147	0	0	0.000110011	0.423710797
denovo_4418	0	0	-0.000389156	0.423710797
denovo_479	0	0	0.041375796	0.423710797
denovo_49	0	0	-0.001712284	0.423710797
denovo_4972	0	0	2.55E-05	0.423710797
denovo_506	0	0	-5.20E-05	0.423710797
denovo_525	0	0	0.001528662	0.423710797
denovo_540	0	0	-0.000985861	0.423710797
denovo_581	0	0	2.56E-05	0.423710797
denovo_6080	0	0	0.002145215	0.423710797
denovo_627	0	0	-5.20E-05	0.423710797
denovo_6539	0	0	-0.00049293	0.423710797
denovo_677	0	0	-2.56E-05	0.423710797
denovo_6794	0	0	-0.000337268	0.423710797
denovo_804	0	0	0.000356688	0.423710797
denovo_8251	0	0	0.000202711	0.423710797
denovo_998	0	0	0.000203822	0.423710797
denovo_11548	0	0	-0.000442426	0.440686016
denovo_11692	0	0	-0.000598031	0.440686016
denovo_2141	0	0	-0.000616345	0.440686016
denovo_2396	0	0	-0.00091769	0.440686016
denovo_2505	0	0	-0.000859085	0.440686016
Glomus_cf	-0.76203795	-0.002938852	-0.016075199	0.547619048
Sclerocystis_sinuosa_MD12(	0	0	0.010620338	0.607235544
denovo_1452	0	0	-0.040277919	0.607235544
denovo_1456	0	0	-0.006412999	0.607235544
denovo_1827	0	0	-0.002351539	0.607235544

denovo_2351	0	0	-0.000600583	0.607235544
denovo_507	0	0	-0.000237506	0.607235544
denovo_6085	0	0	-0.007549134	0.607235544
denovo_6490	0	0	-8.80E-05	0.607235544
denovo_8534	0	0	0.00041169	0.607235544
denovo_8715	0	0	-0.000710426	0.607235544
denovo_8728	0	0	-0.001166182	0.607235544
denovo_381	-1.037214987	-0.000133316	-0.035021505	0.666430034
denovo_1440	0	0	0.001896664	0.797169693
denovo_1448	0	0	0.008085355	0.797169693
denovo_1489	0	0	0.016387194	0.797169693
denovo_1633	0	0	-0.00013099	0.797169693
denovo_603	0	0	0.000122753	0.797169693
denovo_6491	0	0	-5.42E-05	0.797169693
denovo_8143	0	0	-5.37E-05	0.797169693
denovo_8743	0	0	-0.001024229	0.797169693
denovo_1460	-Inf	-0.001534723	-0.000953755	0.823664389
denovo_628	Inf	0.000137514	-0.003435825	0.823664389
denovo_6512	Inf	0.000253389	-0.000423693	0.823664389
Rhizophagus_cf	0.764103051	0.086971479	0.038230208	0.841269841
denovo_1994	0	0	-0.000589026	0.906329391
denovo_2	0	0	-0.01490697	0.906329391
denovo_27	0	0	-0.00388974	0.906329391
denovo_4184	0	0	-3.16E-05	0.906329391
denovo_6151	0	0	-0.00539106	0.906329391
Rhizophagus_intraradices	1.557187037	0.000506926	-0.005600593	0.916562645
Rhizophagus_irregularis	0.093280836	0.002161092	0.028668096	1
Funneliformis_geosporum	0	0	-4.06E-07	1
Glomus_aggregatum	0	0	0.000869735	1
denovo_1	0	0	0.004866087	1
denovo_10042	0	0	-0.000143276	1
denovo_10180	0	0	-0.000483988	1
denovo_1035	0	0	0.0001524	1
denovo_10384	0	0	-0.000236341	1
denovo_10746	0	0	-0.001605052	1
denovo_12631	0	0	0.000457667	1
denovo_137	0	0	-2.73E-05	1
denovo_13922	0	0	0.00052391	1
denovo_13955	0	0	-0.000248091	1
denovo_1442	Inf	0.000126695	-0.022288845	1
denovo_1517	0	0	-5.27E-05	1
denovo_1627	0	0	-0.002272091	1
denovo_1835	0	0	-0.001040448	1
denovo_1841	0	0	-0.000442424	1
denovo_1882	0	0	0.000424085	1
denovo_1988	0	0	-1.16E-06	1
denovo_212	0	0	-0.000340449	1
denovo_2348	0	0	-0.001285167	1
denovo_2489	0	0	-0.000525254	1
denovo_2869	0	0	-0.001324527	1
denovo_4642	0	0	-0.000884441	1
denovo_4823	0	0	0.001836259	1
denovo_500	0	0	0.003916577	1
denovo_508	0	0	-7.85E-05	1
denovo_5122	0	0	-0.000620228	1
denovo_552	0	0	0.000719748	1
denovo_8007	0	0	-0.000211756	1

denovo_8579	0	0	-0.000608243	1
denovo_8622	0	0	-0.000262266	1
denovo_8721	0	0	0.000148742	1
denovo_8921	0	0	-0.000972507	1

"Bs1S" vs. "Rs1S"				
tax_name	log2_median_ratio	median_diff	mean_diff	wilcox_p_value
denovo_358	7.210774715	0.018641279	0.049531625	0.011925234
Glomus_sp	-6.419435564	-0.295739675	-0.249712609	0.015873016
denovo_1903	-Inf	-0.001773724	-0.001514709	0.02536986
denovo_627	Inf	0.000424628	0.000803935	0.02536986
denovo_681	Inf	0.000983445	0.000845898	0.02536986
Rhizophagus_irregularis	-7.888451075	-0.034370285	-0.056347176	0.036145142
denovo_4639	-Inf	-0.000383926	-0.001011129	0.04490859
denovo_581	Inf	0.005297412	0.004985555	0.04490859
denovo_1726	Inf	0.000566171	0.003196318	0.072005658
denovo_506	Inf	0.000582666	0.00336784	0.072005658
denovo_561	Inf	0.00065563	0.001786926	0.072005658
denovo_628	-Inf	-0.000137514	-0.000637812	0.072005658
denovo_6512	-Inf	-0.000253389	-0.000484932	0.072005658
denovo_677	Inf	0.000302709	0.00079617	0.072005658
denovo_919	Inf	0.000145666	0.000155238	0.072005658
denovo_344	4.680306437	0.006243426	0.006225768	0.090688366
Rhizophagus_cf	-7.044454236	-0.209914131	-0.155784535	0.093692619
Glomus_intraradices_691_BI	-Inf	-0.000511902	-0.026192071	0.161237592
Glomus_invermaium	0	0	0.038820985	0.179712495
denovo_2749	0	0	0.001001307	0.179712495
denovo_4119	0	0	0.017426139	0.179712495
denovo_4210	0	0	8.97E-05	0.179712495
denovo_4823	0	0	-0.002384021	0.179712495
denovo_4932	0	0	0.000209621	0.179712495
denovo_6491	0	0	-0.000101772	0.179712495
denovo_6815	0	0	-0.000101868	0.179712495
denovo_6828	0	0	0.086962053	0.179712495
denovo_6832	0	0	0.008122518	0.179712495
denovo_6833	0	0	0.001992376	0.179712495
denovo_6835	0	0	0.004123174	0.179712495
denovo_6851	0	0	0.001631779	0.179712495
denovo_6899	0	0	0.001402309	0.179712495
denovo_6955	0	0	0.001959594	0.179712495
denovo_8534	0	0	-0.000516061	0.179712495
denovo_8743	0	0	-0.000228218	0.179712495
denovo_381	2.592843596	0.000637637	0.032939045	0.204500533
denovo_4480	-Inf	-0.000383926	-0.01785309	0.239316541
Glomus_cf	-Inf	-0.004223189	-0.000454671	0.290346834
denovo_2	Inf	0.025760793	0.057933491	0.332111978
denovo_1442	3.008668951	0.00089297	0.029327868	0.388629493
Claroideoglomus_drummond	0	0	0.000163908	0.423710797
denovo_10042	0	0	-0.000430761	0.423710797
denovo_10180	0	0	-0.000481439	0.423710797
denovo_10384	0	0	-5.07E-05	0.423710797
denovo_10746	0	0	-0.000247525	0.423710797
denovo_11548	0	0	-2.55E-05	0.423710797
denovo_11692	0	0	-2.55E-05	0.423710797
denovo_12631	0	0	-0.000509554	0.423710797
denovo_13922	0	0	-0.000907591	0.423710797
denovo_13955	0	0	-0.000110011	0.423710797

denovo_1503	0	0	8.74E-05	0.423710797
denovo_209	0	0	0.000407643	0.423710797
denovo_2348	0	0	-7.63E-05	0.423710797
denovo_2674	0	0	-0.000819043	0.423710797
denovo_2869	0	0	-7.64E-05	0.423710797
denovo_3167	0	0	2.91E-05	0.423710797
denovo_3223	0	0	0.000524399	0.423710797
denovo_4121	0	0	0.001604359	0.423710797
denovo_4124	0	0	0.004389284	0.423710797
denovo_4138	0	0	0.007779628	0.423710797
denovo_4139	0	0	0.001150295	0.423710797
denovo_4162	0	0	0.004813077	0.423710797
denovo_4199	0	0	0.000302709	0.423710797
denovo_4228	0	0	0.000302709	0.423710797
denovo_4298	0	0	0.000605418	0.423710797
denovo_4418	0	0	6.05E-05	0.423710797
denovo_4474	0	0	0.000756773	0.423710797
denovo_4499	0	0	0.000151355	0.423710797
denovo_4847	0	0	6.05E-05	0.423710797
denovo_49	0	0	0.000229299	0.423710797
denovo_493	0	0	0.009002123	0.423710797
denovo_4961	0	0	5.83E-05	0.423710797
denovo_4972	0	0	-2.55E-05	0.423710797
denovo_507	0	0	-7.64E-05	0.423710797
denovo_508	0	0	-2.55E-05	0.423710797
denovo_5122	0	0	-0.000275028	0.423710797
denovo_570	0	0	-8.25E-05	0.423710797
denovo_603	0	0	-0.000305072	0.423710797
denovo_6490	0	0	-0.00030253	0.423710797
denovo_711	0	0	0.003541698	0.423710797
denovo_8007	0	0	-0.000101356	0.423710797
denovo_804	0	0	-0.000356688	0.423710797
denovo_8143	0	0	-0.000101356	0.423710797
denovo_8251	0	0	-0.000202711	0.423710797
denovo_843	0	0	0.004720538	0.423710797
denovo_8579	0	0	-0.000203381	0.423710797
denovo_8622	0	0	-5.08E-05	0.423710797
denovo_8715	0	0	-0.000127113	0.423710797
denovo_8721	0	0	-0.000279649	0.423710797
denovo_998	0	0	-0.000203822	0.423710797
Funneliformis_geosporum	0	0	0.024927595	0.440686016
Glomus_aggregatum	0	0	0.006664559	0.440686016
denovo_1448	0	0	-0.019766531	0.440686016
denovo_1517	0	0	0.000207017	0.440686016
denovo_1647	0	0	0.002235422	0.440686016
denovo_1835	0	0	0.000666711	0.440686016
denovo_2141	0	0	0.000259508	0.440686016
denovo_2351	0	0	0.001495467	0.440686016
denovo_6151	0	0	-0.028656048	0.440686016
Rhizophagus_intraradices	-Inf	-0.000767853	0.160230183	0.450643728
Sclerocystis_sinuosa_MD12(	0	0	-0.010692299	0.607235544
denovo_1440	0	0	0.002991167	0.607235544
denovo_1460	0	0	-0.006975986	0.607235544
denovo_1511	0	0	0.001132878	0.607235544
denovo_1994	0	0	-0.000500033	0.607235544
denovo_2505	0	0	0.000408301	0.607235544
denovo_1	Inf	0.011351597	0.128132345	0.655836497

denovo_27	Inf	0.000849257	-0.001697426	0.655836497
Rhizophagus_sp	0	0	-0.003269769	0.797169693
denovo_1447	0	0	0.004351578	0.797169693
denovo_1482	0	0	-0.001432499	0.797169693
denovo_1489	0	0	-0.020828567	0.797169693
denovo_1633	0	0	-0.000573977	0.797169693
denovo_1882	0	0	-0.000460399	0.797169693
denovo_2381	0	0	-0.000823292	0.797169693
denovo_2489	0	0	4.40E-05	0.797169693
denovo_4184	0	0	4.86E-05	0.797169693
denovo_634	0	0	-0.000284823	0.797169693
Septoglomus_viscosum	0.193432934	0.000127945	-0.033421163	0.916562645
Glomus_intraradices_x99640	0	0	-0.000294998	1
Septoglomus_constrictum	0	0	0.000503493	1
denovo_1035	0	0	-3.68E-05	1
denovo_137	0	0	0.001146497	1
denovo_1452	0	0	-0.034166108	1
denovo_1453	0	0	0.002271937	1
denovo_1456	0	0	-0.007065561	1
denovo_1493	0	0	-0.000299307	1
denovo_1621	-Inf	-0.000255951	0.002560533	1
denovo_1627	0	0	-0.000658103	1
denovo_1827	0	0	-0.000674868	1
denovo_1841	0	0	2.71E-06	1
denovo_1988	0	0	5.77E-06	1
denovo_2031	0	0	-0.000216799	1
denovo_212	0	0	-7.04E-07	1
denovo_2396	0	0	-2.25E-05	1
denovo_4147	0	0	0.004006835	1
denovo_4642	0	0	6.52E-05	1
denovo_479	0	0	-0.034836518	1
denovo_500	0	0	-0.00277707	1
denovo_525	0	0	-0.001302194	1
denovo_552	0	0	-0.001415428	1
denovo_553	0	0	9.70E-06	1
denovo_6080	0	0	-0.002028681	1
denovo_6085	0	0	-0.005241096	1
denovo_8728	0	0	-0.000423527	1
denovo_8921	0	0	-9.88E-05	1

"Bs2S" vs. "Rs2S"

tax_name	log2_median_ratio	median_diff	mean_diff	wilcox_p_value
denovo_660	-Inf	-0.000518874	-0.000754763	0.007494958
denovo_10711	-Inf	-0.000639468	-0.001247872	0.02536986
denovo_358	Inf	0.067281465	0.083898099	0.02536986
denovo_479	Inf	0.000517264	0.059962655	0.02536986
denovo_581	Inf	0.002322281	0.008412018	0.02536986
denovo_10732	-Inf	-0.000521853	-0.001169496	0.072005658
denovo_11975	-Inf	-0.000767362	-0.000646804	0.072005658
denovo_1460	-Inf	-0.001534723	-0.014185933	0.072005658
denovo_1647	-Inf	-0.000780031	-0.000567821	0.072005658
denovo_561	Inf	0.002967359	0.028994902	0.072005658
denovo_843	Inf	0.000516062	0.004631782	0.072005658
denovo_919	Inf	0.000129016	0.000751512	0.072005658
Glomus_cf	-Inf	-0.007162041	-0.01928302	0.074639183
denovo_4162	-Inf	-0.000780031	-0.000648991	0.157939311
denovo_677	Inf	0.000129887	0.004913863	0.157939311

denovo_6815	-Inf	-0.001023149	-0.001010812	0.157939311
Septoglomus_viscosum	0	0	0.017947174	0.179712495
denovo_10703	0	0	-0.001225758	0.179712495
denovo_10772	0	0	-0.00044283	0.179712495
denovo_10871	0	0	-0.000416454	0.179712495
denovo_11492	0	0	-0.000415959	0.179712495
denovo_11548	0	0	-0.000467904	0.179712495
denovo_11692	0	0	-0.000623509	0.179712495
denovo_11699	0	0	-0.000805172	0.179712495
denovo_13084	0	0	-0.000797685	0.179712495
denovo_1440	0	0	-0.001481887	0.179712495
denovo_1452	0	0	-0.077954289	0.179712495
denovo_1456	0	0	-0.016988822	0.179712495
denovo_1489	0	0	-0.005857626	0.179712495
denovo_1633	0	0	-0.001423619	0.179712495
denovo_1827	0	0	-0.003139641	0.179712495
denovo_1994	0	0	-0.001173985	0.179712495
denovo_2141	0	0	-0.000643848	0.179712495
denovo_2396	0	0	-0.000968536	0.179712495
denovo_2674	0	0	0.044680899	0.179712495
denovo_2749	0	0	0.000700362	0.179712495
denovo_3167	0	0	0.000465652	0.179712495
denovo_3223	0	0	0.000595995	0.179712495
denovo_4121	0	0	-0.000128738	0.179712495
denovo_4228	0	0	0.000569914	0.179712495
denovo_4499	0	0	-0.002548282	0.179712495
denovo_4847	0	0	-0.001046458	0.179712495
denovo_525	0	0	0.002793185	0.179712495
denovo_6085	0	0	-0.012848497	0.179712495
denovo_6151	0	0	-0.034076241	0.179712495
denovo_681	0	0	7.77E-05	0.179712495
denovo_804	0	0	0.00075195	0.179712495
denovo_8715	0	0	-0.00083754	0.179712495
denovo_8728	0	0	-0.001674635	0.179712495
denovo_9089	0	0	-0.000444818	0.179712495
denovo_930	0	0	0.001583053	0.179712495
denovo_570	-Inf	-0.000639468	-0.000861375	0.23586131
denovo_627	Inf	0.000129016	0.006647074	0.239316541
denovo_1447	-Inf	-0.004940198	-0.097405133	0.265205393
Rhizophagus_cf	-7.326588795	-0.123769086	-0.091349537	0.30952381
Glomus_intraradices_x99640	0	0	2.63E-05	0.423710797
Claroideoglomus_drummond	0	0	0.001629381	0.423710797
Glomus_invermaium	0	0	-5.12E-05	0.423710797
Glomus_aggregatum	0	0	-2.61E-05	0.423710797
denovo_10042	0	0	-0.000574038	0.423710797
denovo_10180	0	0	-0.000965427	0.423710797
denovo_10384	0	0	-0.000287019	0.423710797
denovo_10746	0	0	-0.001852577	0.423710797
denovo_12631	0	0	-5.19E-05	0.423710797
denovo_13922	0	0	-0.000383681	0.423710797
denovo_13955	0	0	-0.000358102	0.423710797
denovo_1503	0	0	-0.003198128	0.423710797
denovo_1627	0	0	-0.002958502	0.423710797
denovo_1835	0	0	-0.001066043	0.423710797
denovo_1841	0	0	-0.000468019	0.423710797
denovo_1882	0	0	-0.000153472	0.423710797
denovo_209	0	0	2.60E-05	0.423710797

denovo_212	0	0	-0.00049402	0.423710797
denovo_2348	0	0	-0.001361435	0.423710797
denovo_2489	0	0	-0.000780031	0.423710797
denovo_4119	0	0	0.000465537	0.423710797
denovo_4124	0	0	-0.000181606	0.423710797
denovo_4199	0	0	0.000620716	0.423710797
denovo_4210	0	0	0.00121557	0.423710797
denovo_4642	0	0	-0.000910036	0.423710797
denovo_493	0	0	0.004928396	0.423710797
denovo_4932	0	0	0.000724169	0.423710797
denovo_4961	0	0	0.000284495	0.423710797
denovo_4972	0	0	0.00056899	0.423710797
denovo_711	0	0	0.000696684	0.423710797
denovo_8251	0	0	0.000315333	0.423710797
denovo_8579	0	0	-0.000811625	0.423710797
denovo_8622	0	0	-0.000313112	0.423710797
denovo_8721	0	0	-0.000130907	0.423710797
denovo_8743	0	0	-0.001252446	0.423710797
denovo_8921	0	0	-0.00109962	0.423710797
denovo_998	0	0	0.000311729	0.423710797
Funneliformis_geosporum	0	0	0.014197557	0.440686016
denovo_1035	0	0	0.000595945	0.440686016
denovo_1442	0	0	-0.022489648	0.440686016
denovo_2505	0	0	-0.000935488	0.440686016
denovo_500	0	0	0.006250079	0.440686016
denovo_628	0	0	-0.004047834	0.440686016
denovo_531	-Inf	-0.000129719	5.21E-05	0.48017689
denovo_137	0	0	0.001064511	0.607235544
denovo_2351	0	0	-0.00065004	0.607235544
denovo_4138	0	0	-0.021153486	0.607235544
denovo_508	0	0	0.00028374	0.607235544
denovo_552	0	0	0.002619871	0.607235544
denovo_603	0	0	-7.91E-05	0.607235544
denovo_553	-Inf	-0.000390016	-0.000342093	0.655836497
Rhizophagus_sp	-5.057136541	-0.004175968	0.000998654	0.672335808
Glomus_sp	0.466194427	0.075702878	0.030880175	0.69047619
denovo_1726	0	0	-0.014820898	0.724081661
denovo_2	0	0	-0.017578661	0.724081661
denovo_507	0	0	0.0006718	0.724081661
Glomus_intraradices_691_BI	0	0	0.019041888	0.797169693
denovo_1903	0	0	0.00112636	0.797169693
denovo_2869	0	0	-0.001115557	0.797169693
denovo_4139	0	0	0.000581711	0.797169693
denovo_4184	0	0	0.000944262	0.797169693
denovo_4474	0	0	0.001870268	0.797169693
denovo_4823	0	0	0.001134014	0.797169693
denovo_540	0	0	-0.000778216	0.797169693
denovo_6512	0	0	0.000687815	0.797169693
denovo_8143	0	0	0.00026543	0.797169693
denovo_634	-0.659614197	-0.000380804	0.000332631	0.829356961
Rhizophagus_irregularis	-0.108957736	-0.002353567	0.031326122	0.841269841
denovo_1	0	0	0.04648404	0.906329391
denovo_558	0	0	-0.000287577	0.906329391
Rhizophagus_intraradices	-0.016096661	-2.90E-06	0.029023921	1
Sclerocystis_sinuosa_MD12(	0	0	-0.00646703	1
denovo_1448	0	0	-0.013837518	1
denovo_1517	0	0	0.000259703	1

denovo_1621	0	0	-1.03E-08	1
denovo_1988	0	0	0.001361241	1
denovo_27	Inf	0.000258632	-0.003924462	1
denovo_2958	0	0	-0.00036301	1
denovo_344	-2.704924203	-0.001427709	-0.045694435	1
denovo_381	-1.00766945	-0.000130694	0.015453191	1
denovo_4298	0	0	0.001997167	1
denovo_4418	0	0	-0.000362984	1
denovo_4480	0	0	0.077539014	1
denovo_4639	-Inf	-0.000129719	0.003141736	1
denovo_49	0	0	-0.001557466	1
denovo_498	0	0	0.000127976	1
denovo_506	0	0	0.00219287	1
denovo_5122	0	0	-0.000843529	1
denovo_6490	0	0	0.002017403	1
denovo_6491	0	0	0.000184219	1
denovo_6539	0	0	-0.000335903	1
denovo_6794	0	0	-0.000311097	1
denovo_8007	0	0	0.00037011	1
denovo_8534	0	0	7.41E-07	1

Table S5. LEfSe analysis results using non-parametric factorial Kruskal-Wallis (KW) sum-rank test. Adjusted p-value cut-off=0.05 and LDA score = 1.0. Significant taxa are highlighted in yellow.

Taxa	P-values	FDR	Bs1S	Rs1S	Bs2S	Rs2S	LDAscore
denovo_660	0.00034576	0.05705	0	0	0	7547.6	3.58
denovo_10711	0.0028636	0.23624	0	0	0	12479	3.8
denovo_358	0.0061044	0.33574	496850	1532	838980	0	5.62
denovo_681	0.01314	0.44155	8459	0	777.04	0	3.63
denovo_581	0.014419	0.44155	50112	255.95	84120	0	4.62
denovo_11975	0.018732	0.44155	0	0	0	6468	3.51
denovo_10732	0.018732	0.44155	0	0	0	11695	3.77
Glomus_sp	0.033341	0.45831	433300	2930400	2107900	1799100	6.1
denovo_570	0.037711	0.45831	0	825.08	3619.4	12233	3.79
denovo_627	0.03886	0.45831	8039.4	0	66991	520.02	4.52
Rhizophagus_irregularis	0.04438	0.45831	51312	614780	641360	328100	5.47
denovo_561	0.047572	0.45831	17869	0	289950	0	5.16
denovo_919	0.049078	0.45831	1552.4	0	7515.1	0	3.58
denovo_479	0.062191	0.45831	65393	413760	599630	0	5.48
Septoglomus_viscosum	0.066111	0.45831	143600	477810	179470	0	5.38
denovo_1903	0.074121	0.45831	0	15147	13347	2083.7	3.88
Glomus_cf	0.086392	0.45831	35383	39930	7851.3	200680	4.98
denovo_531	0.09508	0.45831	0	0	3870.5	3349.4	3.29
denovo_843	0.09508	0.45831	47205	0	46318	0	4.37
denovo_677	0.09515	0.45831	7961.7	0	49394	255.79	4.39
denovo_10871	0.097217	0.45831	0	0	0	4164.5	3.32
denovo_11492	0.097217	0.45831	0	0	0	4159.6	3.32
denovo_10772	0.097217	0.45831	0	0	0	4428.3	3.35
denovo_9089	0.097217	0.45831	0	0	0	4448.2	3.35
denovo_11699	0.097217	0.45831	0	0	0	8051.7	3.6
denovo_13084	0.097217	0.45831	0	0	0	7976.9	3.6
denovo_10703	0.097217	0.45831	0	0	0	12258	3.79
denovo_6899	0.097217	0.45831	14023	0	0	0	3.85
denovo_930	0.097217	0.45831	0	0	15831	0	3.9
denovo_6851	0.097217	0.45831	16318	0	0	0	3.91
denovo_6955	0.097217	0.45831	19596	0	0	0	3.99
denovo_6833	0.097217	0.45831	19924	0	0	0	4
denovo_6835	0.097217	0.45831	41232	0	0	0	4.31
denovo_6832	0.097217	0.45831	81225	0	0	0	4.61
denovo_6828	0.097217	0.45831	869620	0	0	0	5.64
denovo_6815	0.13695	0.62767	0	1018.7	523.42	10632	3.73
denovo_628	0.17511	0.68591	0	6378.1	258.03	40736	4.31
denovo_4639	0.18259	0.68591	605.42	10717	32459	1041.3	4.2
denovo_1647	0.18508	0.68591	23454	1100.1	0	5678.2	4.07
denovo_506	0.18965	0.68591	33678	0	22449	520.02	4.23
denovo_4162	0.18965	0.68591	48131	0	1308.6	7798.5	4.38
denovo_558	0.1951	0.68591	0	0	3101.6	5977.4	3.48
denovo_2749	0.1951	0.68591	10013	0	7003.6	0	3.7
denovo_498	0.19704	0.68591	0	0	3098.1	1818.4	3.19
denovo_1726	0.21157	0.68591	31963	0	775.84	148980	4.87
denovo_11548	0.22218	0.68591	0	254.78	0	4679	3.37
denovo_11692	0.22218	0.68591	0	254.78	0	6235.1	3.49
denovo_4847	0.22218	0.68591	605.42	0	0	10465	3.72
denovo_4119	0.22218	0.68591	174260	0	4655.4	0	4.94
Glomus_invermaium	0.22218	0.68591	388210	0	0	511.57	5.29
Rhizophagus_cf	0.22822	0.68591	657410	2215300	919460	1833000	5.89
denovo_3167	0.25441	0.68591	291.33	0	4656.5	0	3.37
denovo_4228	0.25441	0.68591	3027.1	0	5699.1	0	3.45
denovo_804	0.25441	0.68591	0	3566.9	7519.5	0	3.58
denovo_8715	0.25441	0.68591	0	1271.1	0	8375.4	3.62
denovo_1511	0.25441	0.68591	14656	3327.4	0	0	3.87
denovo_4499	0.25441	0.68591	1513.5	0	0	25483	4.11
denovo_2674	0.25441	0.68591	0	8190.4	446810	0	5.35
denovo_1460	0.25869	0.68591	62562	132320	0	141860	4.85
denovo_3223	0.28389	0.68591	5244	0	5960	0	3.47

Taxa	P-values	FDR	Bs1S	Rs1S	Bs2S	Rs2S	LDAscore
denovo_4932	0.28389	0.68591	2096.2	0	7241.7	0	3.56
denovo_540	0.28389	0.68591	0	0	2076.4	9858.6	3.69
denovo_2381	0.28389	0.68591	3593.3	11826	0	0	3.77
denovo_4210	0.28389	0.68591	896.75	0	12156	0	3.78
denovo_8743	0.28389	0.68591	0	2282.2	0	12524	3.8
denovo_4121	0.28389	0.68591	16044	0	0	1287.4	3.9
denovo_1482	0.28389	0.68591	4871.3	19196	0	0	3.98
denovo_553	0.30467	0.68591	605.42	508.45	2845.3	6266.2	3.46
denovo_6512	0.31017	0.68591	0	4849.3	15964	9086.3	3.9
Rhizophagus_sp	0.33633	0.68591	11307	44004	64693	54706	4.43
denovo_1621	0.34161	0.68591	44986	19381	261.71	261.81	4.35
Glomus_intraradices_691_BEG144	0.35434	0.68591	41448	303370	240010	49591	5.12
denovo_2141	0.35505	0.68591	2870.1	275.03	0	6438.5	3.51
denovo_6151	0.36353	0.68591	291.33	286850	0	340760	5.23
denovo_4298	0.3664	0.68591	6054.2	0	72035	52064	4.56
denovo_634	0.37827	0.68591	1737.8	4586	12960	9633.8	3.75
denovo_507	0.384	0.68591	0	764.33	9857.4	3139.4	3.69
denovo_344	0.39771	0.68591	65063	2805.6	32185	489130	5.39
denovo_2396	0.41015	0.68591	283.09	508.45	0	9685.4	3.69
Glomus_aggregatum	0.41015	0.68591	75604	8958.3	0	260.93	4.58
denovo_1994	0.43448	0.68591	849.26	5849.6	0	11740	3.77
denovo_6490	0.43781	0.68591	0	3025.3	24080	3905.6	4.08
denovo_1442	0.43946	0.68591	296600	3316.6	1308.6	226210	5.17
denovo_2	0.44455	0.68591	637430	58094	31377	207160	5.48
denovo_508	0.45517	0.68591	0	254.78	3877.4	1040	3.29
denovo_8728	0.45517	0.68591	849.26	5084.5	0	16746	3.92
denovo_525	0.45517	0.68591	2264.7	15287	27932	0	4.15
denovo_1827	0.45517	0.68591	1132.3	7881	0	31396	4.2
denovo_6085	0.45517	0.68591	582.67	52994	0	128480	4.81
denovo_1452	0.45517	0.68591	35103	376760	0	779540	5.59
denovo_1440	0.46169	0.68591	63697	33786	0	14819	4.5
denovo_4823	0.46871	0.68591	0	23840	16818	5477.6	4.08
denovo_1489	0.48299	0.68591	14163	222450	0	58576	5.05
denovo_1633	0.49392	0.68591	7186.5	12926	0	14236	3.85
denovo_1835	0.4953	0.68591	6923.1	255.95	0	10660	3.73
denovo_8534	0.50409	0.68591	0	5160.6	1051.1	1043.7	3.41
denovo_1456	0.50409	0.68591	35103	105760	0	169890	4.93
denovo_4138	0.50409	0.68591	77796	0	90658	302190	5.18
denovo_4480	0.53588	0.68591	2421.7	180950	840100	64710	5.62
denovo_603	0.54276	0.68591	0	3050.7	1032.1	1823.2	3.18
denovo_2869	0.54276	0.68591	0	764.33	2854	14010	3.85
denovo_4961	0.54873	0.68591	582.67	0	2845	0	3.15
denovo_8721	0.54873	0.68591	0	2796.5	0	1309.1	3.15
denovo_10384	0.54873	0.68591	0	506.78	0	2870.2	3.16
denovo_8622	0.54873	0.68591	0	508.45	0	3131.1	3.19
denovo_998	0.54873	0.68591	0	2038.2	3117.3	0	3.19
denovo_8251	0.54873	0.68591	0	2027.1	3153.3	0	3.2
denovo_2031	0.54873	0.68591	1132.3	3300.3	0	0	3.22
denovo_6794	0.54873	0.68591	0	0	261.71	3372.7	3.23
denovo_13955	0.54873	0.68591	0	1100.1	0	3581	3.25
denovo_1493	0.54873	0.68591	1132.3	4125.4	0	0	3.31
denovo_209	0.54873	0.68591	4076.4	0	259.77	0	3.31
denovo_6539	0.54873	0.68591	0	0	1570.3	4929.3	3.39
denovo_12631	0.54873	0.68591	0	5095.5	0	518.87	3.41
denovo_2958	0.54873	0.68591	0	0	1558.6	5188.7	3.41
denovo_4972	0.54873	0.68591	0	254.78	5689.9	0	3.45
denovo_10042	0.54873	0.68591	0	4307.6	0	5740.4	3.46
denovo_4199	0.54873	0.68591	3027.1	0	6207.2	0	3.49
denovo_8579	0.54873	0.68591	0	2033.8	0	8116.2	3.61
denovo_13922	0.54873	0.68591	0	9075.9	0	3836.8	3.66
denovo_10180	0.54873	0.68591	0	4814.4	0	9654.3	3.68
denovo_2348	0.54873	0.68591	0	762.68	0	13614	3.83
Claroideoglomus_drummondii	0.54873	0.68591	1639.1	0	16294	0	3.91

Taxa	P-values	FDR	Bs1S	Rs1S	Bs2S	Rs2S	LDAscore
denovo_10746	0.54873	0.68591	0	2475.2	0	18526	3.97
denovo_6080	0.54873	0.68591	1165.3	21452	0	0	4.03
denovo_1453	0.54873	0.68591	25195	2475.2	0	0	4.1
denovo_1503	0.54873	0.68591	874	0	0	31981	4.2
denovo_711	0.54873	0.68591	35417	0	6966.8	0	4.25
denovo_4147	0.54873	0.68591	41168	1100.1	0	0	4.31
Septoglomus_constrictum	0.54873	0.68591	41614	36579	0	0	4.32
denovo_4124	0.54873	0.68591	43893	0	0	1816.1	4.34
denovo_493	0.54873	0.68591	90021	0	49284	0	4.65
denovo_1447	0.57276	0.69268	346600	303080	2857.4	976910	5.69
denovo_6491	0.58353	0.69268	0	1017.7	3402.3	1560.1	3.23
denovo_8143	0.58353	0.69268	0	1013.6	4204.4	1550.1	3.32
denovo_1882	0.58353	0.69268	1171.6	5775.6	0	1534.7	3.46
denovo_2489	0.58353	0.69268	2987.8	2547.8	0	7800.3	3.59
denovo_4139	0.58353	0.69268	11503	0	13664	7847.3	3.83
denovo_4474	0.58353	0.69268	7567.7	0	25489	6786.7	4.11
denovo_381	0.61071	0.71976	332200	2810.6	507560	353030	5.4
Funneliformis_geosporum	0.63544	0.7436	249530	255.95	142240	260.01	5.1
denovo_2505	0.67666	0.78626	5106.8	1023.8	259.77	9614.7	3.67
denovo_1035	0.68906	0.79507	1415.4	1783.4	6218.9	259.44	3.47
denovo_2351	0.72331	0.81732	16229	1273.9	779.32	7279.7	3.89
denovo_500	0.74492	0.81732	15287	43057	66392	3891.6	4.49
denovo_4418	0.76778	0.81732	605.42	0	261.71	3891.6	3.29
denovo_1841	0.76778	0.81732	283.09	255.95	0	4680.2	3.37
denovo_212	0.76778	0.81732	1528.7	1535.7	0	4940.2	3.39
denovo_8007	0.76778	0.81732	0	1013.6	6832.2	3131.1	3.53
denovo_5122	0.76778	0.81732	0	2750.3	517.26	8952.6	3.65
denovo_4642	0.76778	0.81732	908.13	255.95	0	9100.4	3.66
denovo_8921	0.76778	0.81732	283.09	1271.1	0	10996	3.74
denovo_49	0.76778	0.81732	2293	0	1548.2	17123	3.93
denovo_1627	0.76778	0.81732	283.09	6864.1	0	29585	4.17
Glomus_intraradices_x99640_BEG141	0.76778	0.81732	50955	53905	262.78	0	4.43
denovo_1517	0.79245	0.83227	2323.6	253.39	3377.1	780.03	3.19
denovo_1448	0.79245	0.83227	22081	219750	517.26	138890	5.04
denovo_552	0.80201	0.83227	3680.1	17834	36836	10637	4.22
Sclerocystis_sinuosa_MD126	0.80201	0.83227	66808	173730	2857.5	67528	4.93
denovo_137	0.84486	0.87127	12229	764.33	11683	1037.7	3.76
Rhizophagus_intraradices	0.91205	0.93471	1626000	23650	369890	79655	5.9
denovo_1	0.92786	0.94504	1566200	284880	701060	236210	5.82
denovo_4184	0.95343	0.96513	3027.1	2541.6	12300	2857.8	3.69
denovo_27	0.97454	0.98048	15013	31987	31640	70884	4.45
denovo_1988	0.99631	0.99631	566.17	508.45	14132	520.02	3.83
